# Exploration of the Tertiary Amide Chemical Space of Dolastatin 15 Analogs Reveals New Insights into the Structure–Anticancer Activity Relationship

**DOI:** 10.1002/cmdc.202500580

**Published:** 2025-08-22

**Authors:** Dayana Alonso, Leslie Reguera, Robert Rennert, Ibrahim Morgan, Mohammed Saoud, Manuel G. Ricardo, Leslie Valdés, Julieta Coro‐Bermello, Ludger A. Wessjohann, Daniel G. Rivera

**Affiliations:** ^1^ Department of Bioorganic Chemistry Leibniz Institute of Plant Biochemistry Weinberg 3 D‐06120 Halle (Saale) Germany; ^2^ Laboratory of Synthetic and Biomolecular Chemistry Faculty of Chemistry University of Havana Zapata & G Havana 10400 Cuba; ^3^ Institute of Pharmacy and Food Chemistry University of Havana 222 Street.Nr.2317 Havana 10600 Cuba

**Keywords:** anticancer compounds, dolastatins, multicomponent reactions, peptides, solid‐phase synthesis

## Abstract

Dolastatins are a class of naturally occurring antimitotic peptides that have inspired the development of some of the most active and widely used anticancer agents. Here, we report on the development of synthetic methodologies for the preparation of parallel libraries of small peptides inspired by dolastatin 15 and its analogs cemadotin and tasidotin. The approaches rely on the use of either one or multiple Ugi‐multicomponent reactions to generate amide *N*‐substituted dolastatin‐like skeletons, which allow the exploration of tertiary amide chemical spaces that have not been assessed previously. Evaluation of the anticancer activity in a variety of cancer cells showed that introducing a tertiary amide at the *C*‐terminal fragment or by replacement of a proline residue does not lead to an increment in the anti‐proliferative activity. The microtubule‐disrupting capacity of the new dolastatin analogs was studied and compared with other potent antimitotic agents, thereby shedding light on mechanistic details of their anti‐proliferative activity.

## Graphical abstract

GA: Parallel libraries of novel dolastatin‐like compounds with different *N*‐substituted backbones were produced using multicomponent reactions as diversification steps. Evaluation of the antiproliferative effect in a variety of cancer cells proved that either the replacement of one proline by a functionalized tertiary amide or introducing a backbone *N*‐substituent at the C‐terminal fragment of cemadotin does not result in an improved anticancer activity but may in some cases alter the primary mode of action.

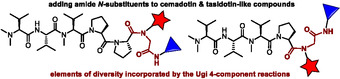

## 1 Introduction

**Figure 1:**
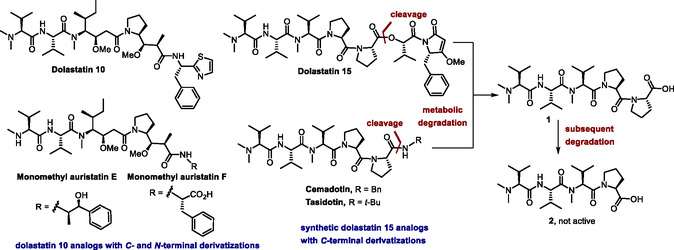
Structures of the natural anticancer peptides dolastatin 10 and dolastatin 15 and some of their synthetic analogs that have advanced to clinical evaluation as anticancer chemotherapeutics. The steps involved in the metabolic inactivation of dolastatin 15 and its analogs via proteolytic cleavage of the peptide backbone are illustrated.

Dolastatins are a family of peptides isolated for the first time by Pettit and co‐workers from the marine mollusk *Dolabella auricularia*.^[^ ^1,^, ^2^ ^]^ The small *pseudo*‐peptides dolastatin 10 and dolastatin 15 (**Figure** **1**) have been the most extensively studied members of this family due to their ability to induce apoptosis in a variety of cancer cells at low nanomolar concentrations.^[^ ^3,^, ^4^ ^]^ As a result, these peptides have been the subject of extensive synthetic programs, initially aimed at enhancing their biological and pharmacological properties^[^ ^1^, ^5^, ^6^, ^7^, ^8^ ^]^ and subsequently to functionalize them for targeting conjugation, e.g., to monoclonal antibodies.^[^ ^9,^, ^10^ ^]^ The derivatization of dolastatin 10 has been highly successful, leading to the development of a class of optimized antimitotic compounds, known as auristatins.^[^ ^9,^, ^10^ ^]^ Members of this compound class, such as monomethyl auristatin E and monomethyl auristatin F, are the cytotoxic warheads of many antibody‐drug conjugates currently approved or in clinical trials.^[^ ^9,^, ^10^ ^]^ Auristatins’ success is a proof that the scaffold of dolastatin 10 accepts certain modifications at both the N‐ and C‐terminus without losing cytotoxicity.^[^ ^7^, ^8^, ^9^, ^10^ ^]^

However, the development of dolastatin 15 and its synthetic analogs has not been equally successful. The *C*‐terminal derivatization of this natural product led to the development of cemadotin^[^ ^11,^, ^12^ ^]^ and tasidotin^[^ ^13,^, ^14^ ^]^ (Figure 1), in which the (*S*)‐2‐hydroxy‐isovaleryl‐(*S*)‐dolapyrrolidone ester moiety of dolastatin 15 was replaced by a benzylamide and a *tert*‐butylamide, respectively, maintaining the potent antimitotic activity. However, both compounds have failed in clinical trials due to insufficient efficacy at tolerable doses.^[^ ^15^, ^16^, ^17^, ^18^, ^19^ ^]^ A common disadvantage of dolastatin 15 and its synthetic analogs cemadotin and tasidotin is their low intracellular stability, as they are rapidly degraded in the lysosomes of targeted cells. Thus, the ester bond in dolastatin 15 and the terminal amide in cemadotin and tasidotin are cleaved by esterases and proteases, respectively, resulting in the formation of peptide **1** (also known as tasidotin C‐carboxylate).^[^ ^20,^, ^21^ ^]^ This peptide metabolite demonstrates a significant level of cytotoxic activity, which is even higher than that of tasidotin. However, it is subject to proteolytic cleavage as well, resulting in the loss of the C‐terminal proline (Pro) residue, leading to the formation of metabolite **2** (Figure 1) that exhibits a reduced effectiveness as an antimitotic agent. The metabolic inactivation of the derivatives of the dolastatin 15 family so far is a key factor contributing to the limited potential of these compounds as anticancer chemotherapeutics. This limitation poses a significant challenge for the clinical applications of antibody‐drug conjugates based on this class of cytotoxic warheads,^[^ ^22^, ^23^, ^24^ ^]^ which is in sharp contrast to the success of dolastatin 10 analogs.

In general, the optimization of anticancer lead compounds and drugs that are already potent does not focus on enhancing drug efficacy. Instead, the objective is to improve synthetic accessibility and ADMET profiles, with a strong focus on reducing toxicity to healthy cells and tissues.^[^ ^25^ ^]^ While the issue of selectivity can be addressed by conjugation to a targeting molecule, the metabolic instability remains a significant challenge in the case of anticancer peptide drugs. It is well‐known that the proteolytic stability is significantly increased when specific backbone nitrogen atoms are alkylated to form tertiary amide bonds.^[^ ^26^, ^27^, ^28^ ^]^ Previously, backbone‐modified analogs of dolastatin 15 have been produced featuring a peptoid structure, but the *N*‐alkylated oligoglycine skeletons did not show cytotoxic activity.^[^ ^29^ ^]^

Our approach does not focus on a fully peptoid skeleton but on exploring a previously uncharted dolastatin 15 chemical space, specifically *N*‐alkyl substituents introduced by either transforming a C‐terminal secondary amide into a tertiary one or replacing a Pro by another *N*‐substituted amino acid residue. It is widely acknowledged that Pro plays a pivotal role in specific peptide conformations, particularly by facilitating access to bent or turn structures. Moreover, the conversion of a secondary amide into a tertiary one, e.g., a straightforward *N*‐methylation, can both result in conformational changes and enhance metabolic stability by circumventing proteolytic cleavage. Accordingly, herein we report on the construction of a library of dolastatin 15 analogs bearing backbone *N‐*modifications that have not been incorporated nor biologically tested in this compound family. The library was designed with the objective of populating the tertiary amide chemical spaces with lipophilic and polar moieties. Specifically, the focus was on replacing either the benzyl and *t*‐butyl secondary amides of cemadotin and tasidotin, respectively, or the preceding Pro—all of which are susceptible to metabolic cleavage—with an *N*‐substituted peptide fragment derived from an Ugi multicomponent reaction (Ugi‐MCR). In addition to the solution‐phase combinatorial chemistry strategy, we present novel solid‐phase approaches that use two consecutive MCRs and orthogonal resin and linker cleavage systems for the synthesis of *N*‐functionalized dolastatin 15 skeletons. Evaluation of the antiproliferative activity of these newly synthesized compounds in various cancer cell lines allows us to assess the impact of these modifications on the anticancer efficacy.

## 2 Results and Discussion

### 2.1 Solution‐Phase Multicomponent Synthesis of C‐Terminally N‐Functionalized Dolastatin 15 Analogs

Considering that the initial step of the metabolic inactivation of dolastatin 15 and its synthetic analogs is the formation of peptide **1**, we sought to carry out a combinatorial derivatization of this pentapeptide using the Ugi‐MCR. This reaction is the condensation of a primary amine, a carboxylic acid, an aldehyde, and an isonitrile to form a dipeptide‐based scaffold,^[^ ^30^ ^]^ in which one of the two amides is a tertiary one. The Ugi‐MCR is particularly well‐suited for the combinatorial functionalization of the peptide backbone, as it enables the incorporation of three additional moieties at the peptide C‐terminus when this latter reacts as the carboxylic acid component. Pentapeptide **1** was produced by using solid‐phase peptide synthesis (SPPS) and then cleaved from the solid support and used in the multicomponent diversification of the C‐terminal backbone fragment (**Table** **1**). The use of paraformaldehyde as the oxo‐component is intended to prevent the formation of diastereomers that would require an extra separation step later.

**Table 1:** Solution‐phase combinatorial synthesis and anticancer activity of dolastatin 15 analogs based on a N‐substituted cemadotin‐like backbone.

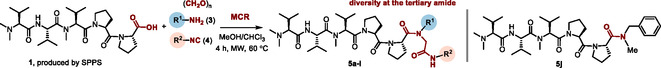						
Peptide	R^1^	R^2^	Yield [%]	PC‐3 IC_50_ [μM]a)	HCT‐116 IC_50_ [μM]a)	MDA‐MB‐468 IC_50_ [μM]a)
5a	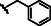	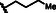	68	1.5	2.2	0.82
5b	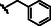	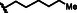	59	≈5	7.2	2.1
5c	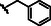	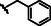	55	1.9	2.3	1.1
5d	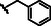	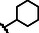	64	1.2	1.9	1.4
5e	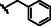	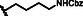	63	1.6	4.3	1.2
5f	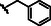	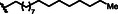	60	2.1	2.7	2.1
5g	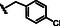	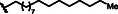	52	8.9	8.3	≈5
5h	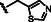	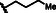	66	0.03	0.05	0.024
5i	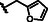	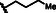	52	3.7	>10	1.2
5jb) (*N*‐methyl‐cemadotin—comparable reference)			62	>10	>10	>10
cemadotinb,c)			46	0.0013	0.001	0.0025

^a)^ The IC_50_ values were calculated based on dose–response curves that are shown along with the calculated errors (95% confidence intervals) in Figure S83 of the Supporting Information;

b) *N*‐Methyl‐cemadotin (**5j**) and cemadotin were synthesized by coupling peptide **1** with *N*‐benzylmethyl amine and benzyl amine, respectively;

c) The structure of cemadotin is shown in Figure 1.

As shown in Table 1, the amine component **3** was initially fixed to benzyl amine to achieve the highest resemblance to cemadotin. Additionally, *para*‐chloro‐benzyl amine, thiazol‐5‐ylmethanamine, and furfuryl amine were utilized as amino components to broaden the diversity of this residue. The protocol involved the preformation of the imine by stirring the amine and the aldehyde, followed by the reaction with peptide **1** and the isonitrile under microwave irradiation at 60 °C. The reactions were completed within 4 h, providing the dolastatin analogs in good to excellent yields after chromatographic purification. Another key source of diversity is the isonitrile component **4**, which introduces an *N*‐substituent bearing a cyclic or linear aliphatic chain of different length, an additional aromatic group or a protected amine for further derivatization or conjugation. It is noteworthy that compounds **5a**‐**f** have the same peptide backbone as cemadotin, however, with the secondary benzylamide replaced by a tertiary one that bears additional functionalities.

The anticancer activity of the dolastatin 15 analogs **5a**‐**i** was tested in comparison with cemadotin and the *N*‐methylated cemadotin derivative **5j** against three human cancer cell lines, namely prostate adenocarcinoma PC‐3 cells, colorectal carcinoma HCT‐116 cells, and triple‐negative breast cancer MDA‐MB‐468 cells. In vitro cell viability and cytotoxicity assays were conducted and IC_50_ curves, and values were determined using the resazurin assay and fluorometric read‐out after 48 h cells’ treatment with the compounds in concentrations covering the micro‐ and nanomolar range (Figure S83, Supporting Information, SI). As shown in Table 1, the majority of these backbone *N*‐modified dolastatin 15 analogs showed IC_50_ values in the low micromolar range. The exception was peptide **5h**—bearing a thiazole moiety instead of a phenyl—which showed nanomolar IC_50_ values just one order of magnitude higher than the IC_50_ values of dolastatin 15.^[^ ^31^ ^]^ Similarly, cemadotin also proved to be one order of magnitude more potent than **5h** in all three cancer cell lines tested (Table 1).

Noteworthy, the compounds **5f** and **5g** are of particular interest as they seem, to our knowledge, to be the first lipidic dolastatins produced and evaluated for their anticancer activity. Our group has been at the forefront of the field of peptide lipidation and assembly using multicomponent strategies. ^[^ 32, 33, 34 ^]^ This approach enables the incorporation of the desired peptide building block (e.g., amino acid) simultaneously with a lipidic tail, typically yielding an *N*‐lipidated tertiary amide as replacement of the original secondary one. In this instance, the benzyl and lipidic (C16) components were incorporated in one pot, arising from the amine and isonitrile, respectively. The rationale behind this approach was a) to increase the compound's lipophilicity and facilitate cell permeability due to an enhanced interaction with the cell membrane and b) to further explore the potential of liposomal formulations specifically designed to either promote cell internalization or improve the compounds’ pharmacological properties. Unfortunately, the lipodolastatins **5f** and **5g** did not demonstrate an enhanced but very similar antiproliferative activity compared to the analogs with shorter aliphatic chains, such as **5a** (C4) and **5b** (C6). However, the cytotoxic profile of these lipodolastatins was somehow different from the other analogs produced by this method. In this sense, most of the compounds with IC_50_ < 10 µM showed a similar trend, acting cytostatic rather than cytotoxic in the three cancer cell lines, with the dose–response curves reaching the lower plateau at ≈20–40% of cell viability (Figure S83 in the Supporting Information). This effect can be interpreted as preventing further cell proliferation, however, without killing all already existing cancer cells. Contrarily, the two lipodolastatins **5f** and **5g** appeared to induce complete cell death at higher concentrations, as their IC_50_ curves indeed reached approximately 0% of cancer cell viability. The underlying reason for this different behavior needs to be investigated in more detail. Nevertheless, we hypothesize that it could be due to a higher in vitro cellular bioavailability resulting from the higher lipophilicity of the long‐chain derivatives, even when it is not obviously reflected in their IC_50_ values.

Dolastatins 10 and 15 are known to disrupt microtubule assembly and tubulin polymerization,^[^ ^4^, ^5^, ^6^, ^7^, ^8^, ^9^, ^10^, ^11^, ^12^, ^13^ ^]^ thus inhibiting mitotic cell division and, hence, cell proliferation. To investigate a possible structural reason for the decreased anticancer activity of the analogs bearing an *N*‐substituted backbone at the *C*‐terminal fragment, we also prepared an *N*‐methylated analog (**5j**) of cemadotin. Remarkably, the simple incorporation of an *N*‐methyl group at the *C*‐terminal amide of cemadotin resulted in a strong loss of its antiproliferative potency. Converting a secondary amide into a tertiary one introduces conformational changes associated with the *s‐cis* and *s‐trans* isomerization, which could affect the binding to the tubulin proteins and, consequently, result in an inefficient inhibition of the microtubule assembly.

On the other hand, it was found that analog **5h** showed a notable anticancer activity, being two orders of magnitude more potent than the other analogs derived from the Ugi‐MCR. This compound incorporates a thiazole moiety at the *C*‐terminus, a structural element that is also present in the highly potent dolastatin 10 (Figure 1). Given that all other combinations led to less active analogs, this suggests that the thiazole moiety has a positive impact on the binding to the tubulin targets, despite the presence of the tertiary amide. Further computational and crystallographic studies may be necessary to gain deeper insights into this protein–ligand interaction.

### 2.2 On‐Resin Synthesis of Internal Backbone Amide N‐Functionalized Dolastatins 15 Analogs

In addition to replacing the *C‐*terminal secondary amide of cemadotin by a tertiary one, we explored a new chemical space arising from the replacement of the Pro residue more adjacent to the *C*‐terminus by an Ugi‐MCR‐derived tertiary amide. As part of a previous study dedicated to simplifying the structure of dolastatin 15,^[^ ^35^ ^]^ this Pro was replaced by a simple *N*‐methyl‐alanine residue, but the cytotoxic activity dropped significantly. In contrast, our strategy here was to consider this amide *N*‐substituent as a position for the introduction of additional structural diversity, rather than just as a small *N*‐substituent intended to maintain the tertiary nature of the amide.

Due to the lack of a *C*‐terminal functionality that could initially be attached to a resin for the subsequent growth of the peptide skeleton, previous syntheses of dolastatin 15 and its synthetic analogs cemadotin and tasidotin have been performed either using solution‐phase approaches or a combination of solution‐ and solid‐phase peptide syntheses (SPPS).^[^ ^36^ ^]^ These previous approaches are analogous to the method illustrated in Table 1, in which a peptide precursor is synthesized by SPPS and then derivatized at the C‐terminus. In contrast, we sought to develop an innovative strategy enabling the all‐on‐resin synthesis of analogs with backbone *N*‐derivatizations.

As shown in **Table** **2**, we developed an on‐resin approach that enables the parallel construction of novel dolastatin‐like compounds with diversity elements at three distinct positions. The novelty lies at the implementation of two consecutive Ugi‐MCRs for the assembly and diversification of the tertiary amide‐based scaffold and the concurrent integration of a photo‐cleavable linker that allows the final release of the dolastatin‐like peptides.^[^ ^37^ ^]^ The approach begins with an on‐resin Ugi‐MCR of a Fmoc‐amino acid (AA, reacting as the carboxylic acid), an amine bearing the variable substituent R^1^, 4,5‐dimethoxy‐2‐nitrobenzaldehyde and the resin‐linked isocyanoacetate **6**. This latter compound was prepared based on commercially available Fmoc‐Gly‐Wang resin by Fmoc removal, formylation of the resulting glycine‐linked resin,^[^ ^38^ ^]^ and final dehydration with POCl_3_ under basic conditions.^[^ ^39^ ^]^ Isonitrile formation on the resin was confirmed by infrared analysis, whereby the band around 2100 cm^−1^ unequivocally confirmed the formation of the triple bond functional group (Figure S46 in the Supporting Information). The incorporation of the 4,5‐dimethoxy‐2‐nitrobenzene moiety in the first Ugi‐MCR—arising from the carbonyl component—aimed at enabling a final photo‐cleavage step for the release of the desired dolastatin‐like scaffold.

**Table 2:** All‐on‐resin synthesis and anticancer activity of backbone amide N‐substituted dolastatin 15 analogs featuring three elements of diversity.

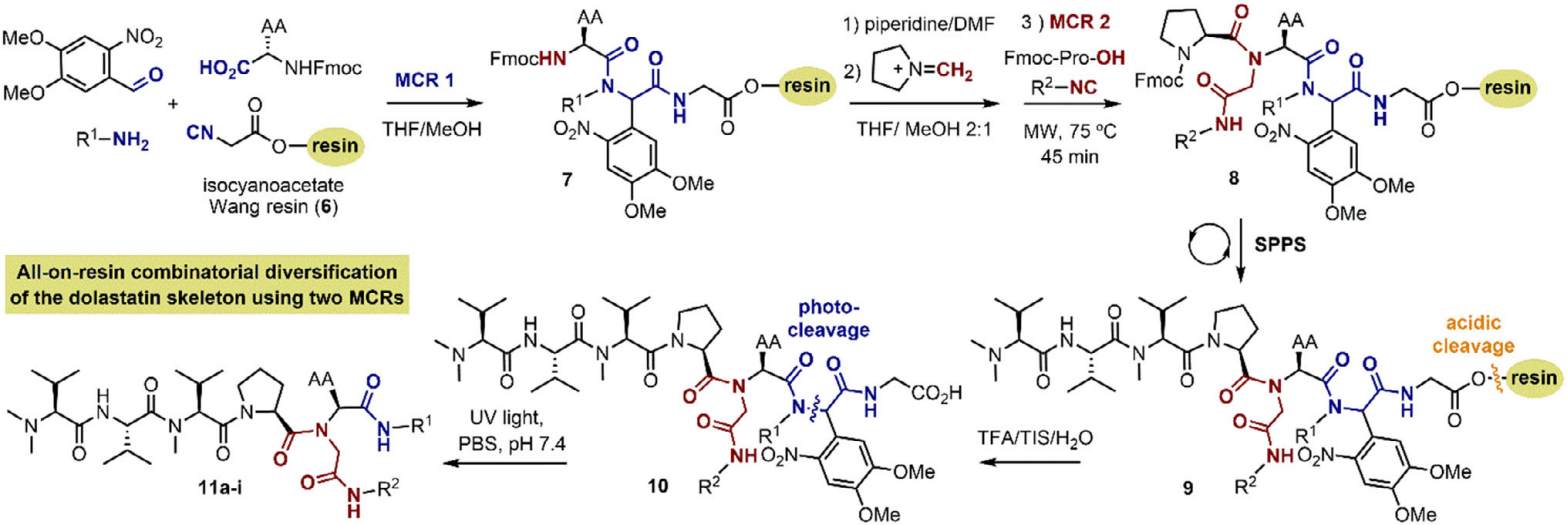							
Peptide	Amino acid (AA)	R^1^	R^2^	Yield [%]b)	PC‐3 IC_50_ [μM]a)	HCT‐116 IC_50_ [μM]a)	MDA‐MB‐468 IC_50_ [μM]a)
11a	Leu	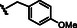	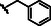	25	>10	>10	>10
11b	Leu	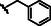	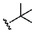	30	>10	>10	>10
11c	Leu	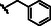	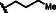	23	>10	7.9	≈5
11d	Phe	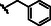	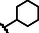	28	>10	>10	>10
11e	Phe	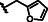	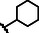	19	9.3	>10	>10
11f	Phe	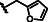	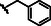	21	>10	>10	>10
11g	Ala	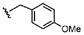	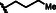	22	>10	>10	>10
11h	Ala	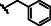	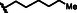	24	>10	ND	ND
11i	*β*‐Ala	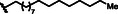	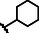	18	2.3	6.4	3.4

^a)^ The IC_50_ values were calculated based on dose–response curves that are shown in the Supporting Information.

^b)^ Overall yields were calculated based on the initial amount of isocyano‐resin (0.1 mmol) after the full on‐resin protocol, the photo‐cleavage step and the RP‐HPLC purification.

To seek a structural similarity with cemadotin, the amine introduced in the first Ugi‐MCR included in most cases an aromatic residue, except for a lipidic amine (C16) used to produce analog **11i**. Four different amino acids were considered as carboxylic acid components of the first Ugi‐MCR, providing further diversity in the library. The third diversity element, arising from the isonitrile component, was introduced with the second Ugi‐MCR, which again allows to incorporate aliphatic or aromatic residues.

Unfortunately, this type of dolastatin‐like scaffold, in which the second Pro residue is replaced by a linear *N*‐substituted amino acid, did not show potent antiproliferative or cytotoxic effects in the panel of cancer cells tested. The only promising compound was the lipodolastatin **11i** derived from the flexible *β*‐Ala residue. As before, this lipidic analog showed a cytotoxic profile inducing the death of all cancer cells tested, albeit its IC_50_ value remains in the low micromolar range (Table 2 and Figure S84 in the Supporting Information). A limitation of this study is that both libraries (Tables 1 and 2) were produced simultaneously and subsequently screened for anticancer activity. Therefore, we could not consider the biological results of one library when designing the other. Accordingly, the incorporation of a thiazole moiety was not originally considered for the construction of the second library, and given the rather poor result obtained, we preferred to focus on a more comprehensive biological study of compounds of library one rather than expanding library two.

### 2.3 Effect of the Dolastatin Analogs on the Tubulin Polymerization

**Figure 2:**
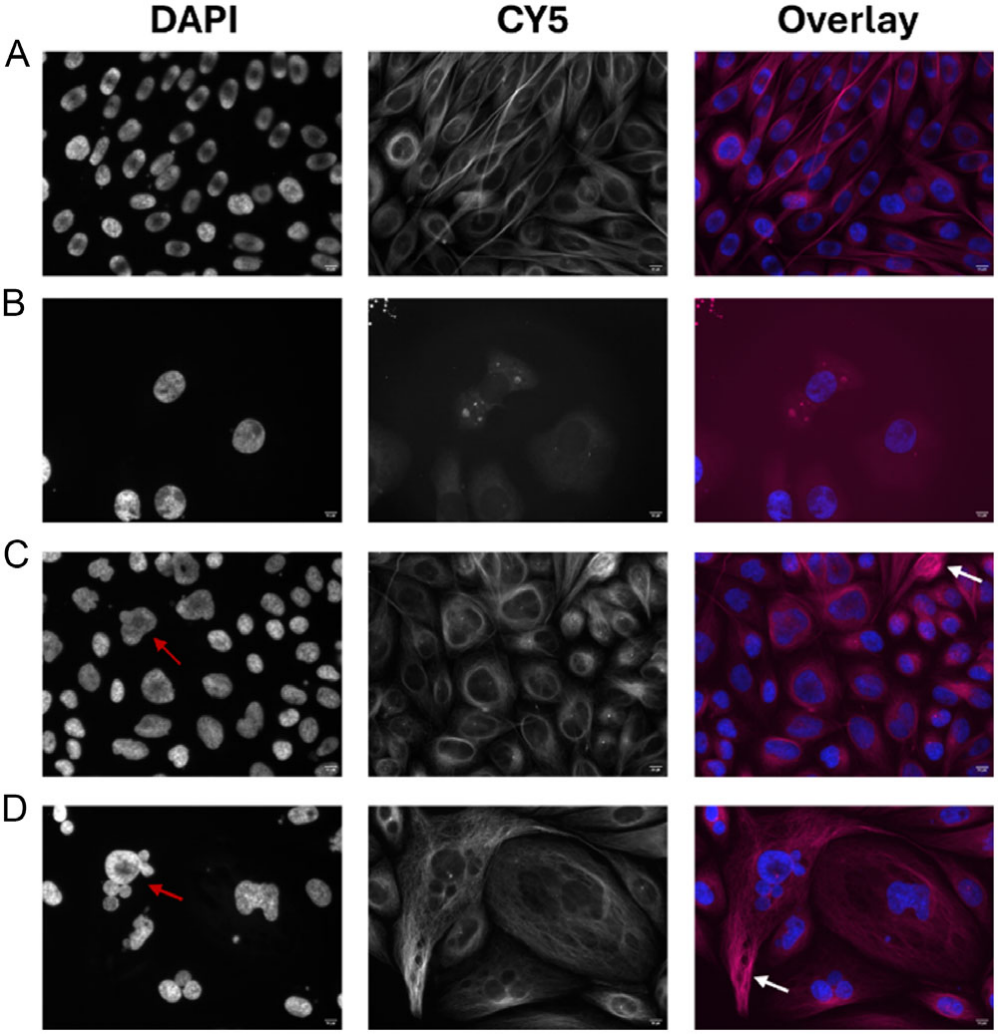
Microscopic analyses of the effect of the dolastatin analogs on microtubule structure. PC‐3 cells were A) left untreated as negative control or were treated for 48 h with IC_50_ concentrations of B) tubugi‐4 (positive control, 0.5 nM), C) compound **5a** (0.03 µM) and D) compound **5h** (1.5 µM). The microtubules are visualized by using the fluorogenic SiR‐tubulin staining that is based on a silicon‐rhodamine‐docetaxel derivative. The tubulin was imaged with Cy5 filter settings and illustrated in magenta in the overlay and the cells’ nuclei were stained with DAPI (blue in the overlay). White arrows highlight areas of denser microtubule filaments. Red arrows highlight enlarged cells with strongly fragmented nuclei.

To study the proposed inhibitory effect of the novel dolastatin analogs on the microtubule structure, we carried out a microscopic investigation using PC‐3 prostate cancer cells with fluorescently labeled tubulin (SiR‐tubulin) treated with the dolastatin analogs **5a** and **5h**. As microtubule‐disrupting positive control, we used a potent tubulysin analog developed by our group, called tubugi‐4^[^ ^40,^, ^41^ ^]^ (see structure in the Supporting Information). **Figure** **2**A shows untreated PC‐3 cells as control sample, with the cells’ nuclei (DAPI, blue in the overlay) and the intact microtubule network (Cy5, magenta in the overlay), in which the tubulin filaments are spread throughout the cytoplasm of the cells. In contrast, the microtubules network disappeared entirely following treatment with the tubulysin analog tubugi‐4 (Figure 2B). This finding is consistent with both the published fluorescent microscopic images of the naturally occurring tubulysin A and tubugi‐4^′^s high antiproliferative activity (IC_50_ = 0.46 nM).^[^ ^40^ ^]^

Analysis of Figure 2C,D confirms that the novel dolastatin analogs **5a** and **5h** also affect the microtubule structures, although their effect on the microtubules appears different from each other. Although compound **5a** does not reach the potency of reference compound tubugi‐4, the micrographs in Figure 2C resemble those published for tubulysins^[^ ^42^ ^]^ and other microtubule disruptors such as vinblastine and disorazole C_1_.^[^ ^43^ ^]^ Accordingly, compound **5a** acts in the initial phase of microtubule network disruption, as previously stated in the cited studies, which is characterized by the retraction of the microtubules from the cellular periphery, resulting in shorter and denser filaments, abnormal accumulation of the microtubules around the periphery of the nuclei (white arrows), and rounded cell morphology. Interestingly, the effect of dolastatin analog **5h** (Figure 2D) appears to be different to that of the parent compound **5a**. Thus, whereas areas of denser microtubule filaments also appear in Figure 2D (white arrow), the cells’ microtubule networks appear to be stabilized rather than destabilized. This looks more similar to those published for paclitaxel and other microtubule‐stabilizing agents, ^[^ 44, 45, 46 ^]^ like the recently discovered *Nerium oleander* monoglycoside action. Noteworthy, after treatment with compound **5h**, the cells were enlarged and showed strongly fragmented nuclei (red arrow), the latter being an indicator of apoptosis induction.

Further studies are required to elucidate the apparently different modes of action of **5a** and **5h** and the structural basis for this distinction. However, it is conceivable that these compounds may exhibit a concentration‐dependent behavior in both directions, akin to vinca alkaloids. The antiproliferative mode of action of these the vinca compopunds has recently been described as being strongly concentration‐dependent. Thus, the microtubule‐destabilizing effect (antipolymerization) has been observed to occur only at high concentrations, while at low concentrations, they acted as microtubule‐stabilizers, also inhibiting microtubule dynamics and thereby exerting an antiproliferative activity.^[^ ^47^ ^]^ The observed discrepancy in the mode of action of these two dolastatin analogs could be partly attributed to the divergent cellular concentrations resulting from their different cell bioavailability. Nonetheless, either scenario leads to the disruption of microtubule dynamics, thereby inducing not only a suppression of cell mitosis but also a substantial impact on various microtubule‐associated cellular functions, including intracellular transport, cellular secretion, and structural integrity.

## 3 Conclusions

We have developed solution‐ and solid‐phase approaches that enable exploring a novel chemical space of dolastatins. Initially, the focus was on replacing the C‐terminal secondary amide of cemadotin—a potent dolastatin 15 analog—by a tertiary amide scaffold derived from an Ugi‐multicomponent derivatization approach to improve biostability. Unfortunately, the new *N*‐substituted cemadotin‐like skeletons did not show enhanced or at least equal activity in comparison with the original parent compound. Interestingly, an analog bearing a thiazole moiety at the *N*‐substituted skeleton showed the best antiproliferative activity, somehow illustrating the positive role of this moiety that is also found in dolastatin 10. The results showed that replacing the C‐terminal secondary amide of cemadotin with a tertiary one—even with a small *N*‐methyl substituent—regularly resulted in a drop in the anticancer activity. Subsequently, a novel on‐resin strategy combining coupling protocols, two Ugi‐multicomponent reactions and a photo‐cleavage step was developed to produce another set of analogs. In this second library, an internal Pro was replaced by a tertiary amide, which allows the introduction of various diversity elements, but led at the same time to a significant decrease of the antiproliferative effect. Since none of the novel compounds demonstrated an improved anticancer activity, further studies regarding the metabolic stability of these new compounds were not considered.

Our results suggest that new studies might be required to better understand the role of the dolastatin amides *cis‐trans* isomerization with respect to their tubulin binding capacity and, ultimately, for the microtubule‐disrupting effect of this compound class. A simple substitution of a proline (tertiary) amide by a linear tertiary amide did not suffice to allow a rational design of novel inhibitors.

## Supporting Information

Synthetic procedures, NMR and MS data and spectra, HPLC chromatograms of the novel compounds and anti‐proliferative assays.

## Conflict of Interest

The authors declare no conflict of interest.

## Supporting information

Supplementary Material

## Data Availability

The data that support the findings of this study are available from the corresponding author upon reasonable request.
