# Supplementary material for: Exploration of the Tertiary Amide Chemical Space of Dolastatin 15 Analogs Reveals New Insights into the Structure–Anticancer Activity Relationship
Source: ChemMedChem. 2025 Aug 22;20(19):e202500580. doi: 10.1002/cmdc.202500580 (PMC12503912; doi:10.1002/cmdc.202500580)
Supplement: Supplementary file 1 — Supplementary Material [file CMDC-20-e202500580-s001.pdf]

Table of Contents

Abbreviations 2

General Information 2

Synthesis of the building blocks 3

General procedure for solid phase peptide synthesis (SPPS) 3

Synthesis of pentapeptide acid NMe_2Val-Val-NMe-Val-Pro-Pro 4

Synthesis of cemadotin 5

General procedure for the multicomponent synthesis of dolastatin 15 analogs 6

Synthesis of *N*-methylcemadotin (5j) 25

Synthesis of isocyanide modified resin 26

All-on-resin synthesis of internal backbone amide *N*-substituted dolastatin 15 analogs 27

Material and methods for cell-based assays 47

Results of cell viability assays 49

Abbreviations

CI, confidence interval; DCM, dichloromethane; DIPEA, diisopropylethylamine; DMF, dimethylformamide; DMSO, dimethylsulfoxide; ESI-MS, electrospray ionization mass spectrometry; FA, formic acid; Fmoc, fluorenylmethyloxycarbonyl; FCS, fetal calf serum; HATU, hexafluorophosphate azabenzotriazole tetramethyl uronium; HOAt, 1-hydroxy-7-azabenzotriazol ; HR-MS, high resolution mass spectrometry; IC_{50}, inhibitory concentration 50% (50% inhibitory effect); IR, infrared; MeOH, methanol; LC/MS, Liquid chromatography/mass spectrometry; NMM, *N*-methylmorpholine; RP-HPLC, reserved-phase high performance liquid chromatography; PBS, phosphate buffer saline; PDA, photo diode array; RT, room temperature; SPPS, solid phase peptide synthesis; THF, tetrahydrofuran; TFA, trifluoroacetic acid; THF, tetrahydrofuran; TIS, triisopropylsilane; TLC, thin layer chromatography; TMS, tetramethyl silane; TNBC, triple-negative breast cancer; Trt, triphenylmethyl.

General Information

All starting materials were purchased from commercial sources and used without further purification. ^{1}H NMR and ^{13}C NMR spectra were recorded either in a Varian Mercury 400 NMR spectrometer at 399.94 MHz and 100.57 MHz, respectively or in an Agilent (Varian) VNMRS 600 NMR spectrometer at 599.83 MHz and 150.83 MHz, respectively. Chemical shifts (δ) are reported in ppm relative to the TMS (^{1}H NMR) and to the solvent signal (^{13}C NMR). A TripleToF 6600-1 mass spectrometer (Sciex) was used for high-resolution mass spectrometry, which was equipped with an ESI-DuoSpray-Ion-Source (it operated in positive ion mode) and was controlled by Analyst 1.7.1 TF software (Sciex). The ESI source operation parameters were as follows: ion spray voltage: 5,500 V, nebulizing gas: 60 p.s.i., source temperature: 450 °C, drying gas: 70 p.s.i., curtain gas: 35 p.s.i. Data acquisition was performed in the MS1-ToF mode, scanned from 100 to 1500 Da with an accumulation time of 50 ms. Analytical RP-HPLC analysis was performed with an Agilent 1100 system in a reverse-phase C18 YMC-ODS-A column (4.6 × 150 mm, 5 µm particle size) with a PDA detector. A linear gradient from 5% to 60 % of solvent B in solvent A over 20 min at a flow rate of 0.8 mL/min was used. Semi and Preparative RP-HPLC was carried out with an Agilent 1260 Infinity series system coupled to a MWD detector and an Agilent 6120 Quadrupole LC/MS detector using API-ES as ion source. Separation was achieved using an RP C18 YMC-ODS-A (150 × 20 mm I.D., 5 µm particle size). A linear gradient from 15% to up 50% or 70% of solvent B in solvent

A over 20 min at a flow rate of 3.8 mL/min or 16 mL/min was used. Solvent A: 0.1% (*v*/*v*) FA in water. Solvent B: 0.1 % (*v*/*v*) FA in acetonitrile.

Synthesis of the building blocks

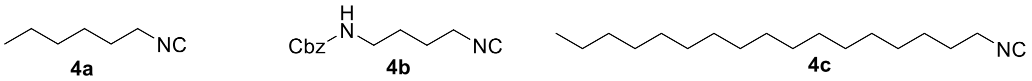

Building blocks **4a**, **4b** and **4c** were used from our laboratory stock or were synthesized according to reported procedures.[1,2]

General procedure for solid phase peptide synthesis (SPPS)

Assembly of the dolastatin sequence by SPPS: The syntheses were carried out manually on different pre-loaded resins by a stepwise Fmoc/*t*Bu strategy at 0.1 mmol scale.

Swelling: The resin is stirred for 20 min in DCM.

Fmoc removal: The resin is treated with a solution of 20% piperidine in DMF (2×10 min), then washed with DCM (3×1 min) and DMF (2×1 min).

HATU/NMM coupling: The Fmoc-protected amino acid (0.4 mmol), HATU (152 mg, 0.4 mmol) and HOAt (27 mg, 0.2 mmol) are dissolved in DMF (3 mL), then NMM (89 µL, 0.8 mmol) is added. The mixture is pre-activated for 4 min, added to the free amino-containing resin (0.1 mmol), and stirred at room temperature until completion or almost completion as indicated by ESI-MS and RP-HPLC monitoring after mini-cleavages. If after 3 h of reaction, more than 95% of conversion is not achieved, the coupling is repeated once again and then the peptide is capped by acetylation. The *N*-terminal Val was dimethylated as follows.

Reductive amination: The peptide-bound resin is mixed with a solution of $NaBH_3CN$ (40 mg, 0.6 mmol) in THF/MeOH containing one drop of acetic acid. A formalin solution (51 µL, 0.8 mmol) is added and the suspension is stirred at room temperature for 90 min and then washed with THF (3×1 min) and DCM (2×1 min). ESI-MS monitoring after mini-cleavages confirmed full conversion.

---

[1] Henze, M.; Kreye, O.; Brauch, S.; Nitsche, C.; Naumann, K.; Wessjohann, L. A.; Westermann, B. Photoaffinity-labeled peptoids and depsipeptides by multicomponent reactions. *Synthesis*, **2010**, 17, 2997 – 3003.

[2] Pérez-Labrada, K.; Brouard. I.; Méndez, I.; G. Rivera, D. Multicomponent Synthesis of Ugi-Type Ceramide Analogues and Neoglycolipids from Lipidic Isocyanides. *J. Org. Chem.* **2012**, 77, 10, 4660–4670.

Cleavage: The resin-linked peptide is treated with the cocktail TFA/TIS/H_2O (95:2.5:2.5) for 1 h and the resin is filtered and washed with the cleavage cocktail. The TFA is evaporated under nitrogen stream and the peptide is redissolved in acetonitrile/water 1:2 (*v/v*) and lyophilized. Purification by preparative RP-HPLC produced the pure dolastatin analogs.

Synthesis of pentapeptide acid NMe_2Val-Val-NMe-Val-Pro-Pro

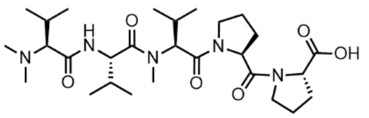

Peptide **1** was assembled from pre-loaded H-Pro-2-chlorotrityl resin (0.6 mmol) according to the general SPPS procedure described above. The peptide was assembled coupling Fmoc-Pro-OH, Fmoc-NMeVal-OH and two times Fmoc-Val-OH. A capping reaction was performed after Fmoc-NMeVal-OH coupling. Finally, a reductive amination allowed the dimethylation at the *N*-terminus. The product was used without further purification in the next step. NMe_2Val-Val-NMeVal-Pro-Pro-OH (295 mg, 89% crude yield, 81% purity. R_t: 7.9 min. ESI-MS: calcd for $C_{28}H_{50}N_5O_6$ ($[M+H]^+$) m/z 552.3761, found 552.3789.

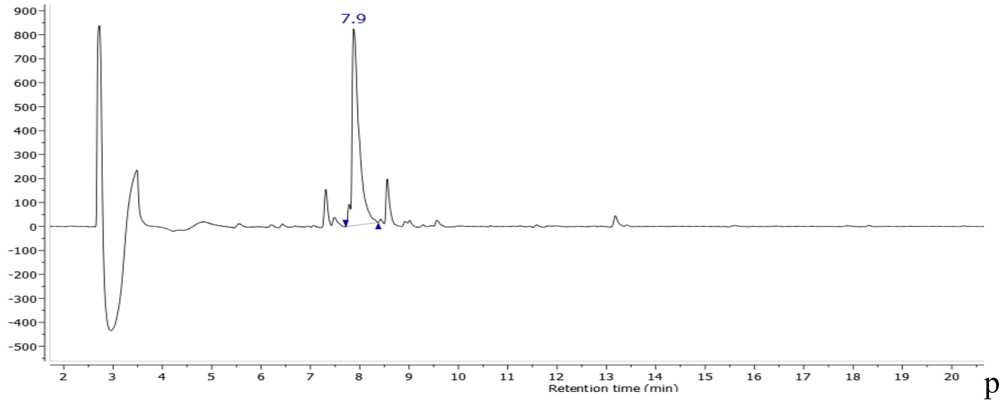

p’

Figure S1. RP-HPLC traces the crude of peptide **1**.

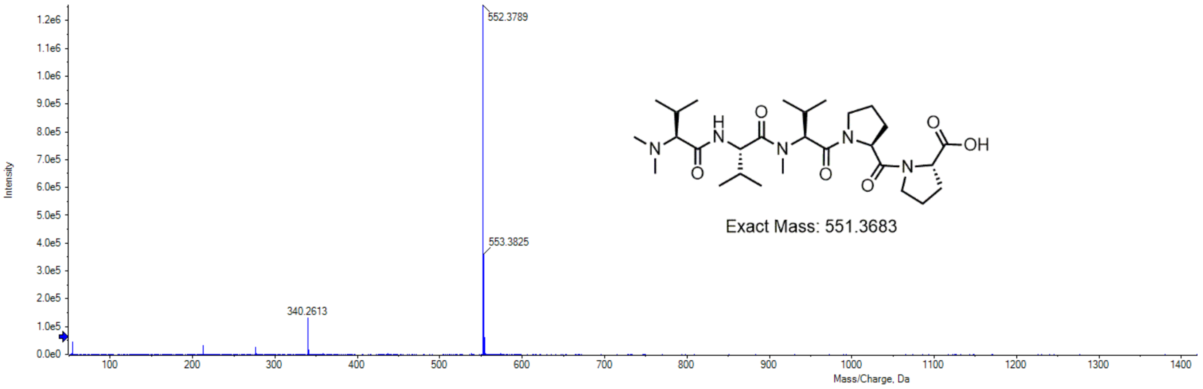

Figure S2. ESI-HRMS of peptide **1**.

Synthesis of Cemadotin

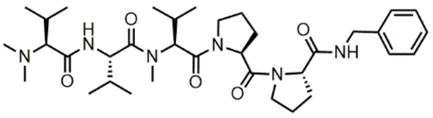

Cemadotin was prepared by coupling benzylamine (5.5 µL, 0.05 mmol) to the crude peptide **1** (13.4 mg, 0.024 mmol) using HATU (19 mg, 0.05 mmol) and NMM (11.1 µL, 0.05 mmol) in DMF for 1 h. Pure cemadotin (7.1 mg, 46% isolated yield, >95% purity) was obtained as the formate salt by RP-HPLC purification. R_t = 13.0 min. ^{1}H NMR (400 MHz, DMSO-d_6): δ 9.49 (s, 1H), 8.89 (s, 1H), 8.27 (t, J = 6.0 Hz, 1H), 8.13 (s, 1H), 7.33 – 7.18 (m, 6H), 4.98 (d, J = 10.9 Hz, 1H), 4.54 (dt, J = 8.5, 4.7 Hz, 2H), 4.37 – 4.29 (m, 2H), 4.20 (d, J = 5.7 Hz, 1H), 4.16 (d, J = 5.8 Hz, 1H), 3.76 – 3.68 (m, 1H), 3.67 – 3.62 (m, 1H), 3.60 – 3.49 (m, 2H), 3.39 (dd, J = 9.2, 5.0 Hz, 1H), 3.07 (s, 3H), 3.05 – 2.98 (m, 1H), 2.94 (s, 1H), 2.85 – 2.60 (m, 5H), 2.20 – 2.04 (m, 4H), 2.03 – 1.86 (m, 4H), 1.86 – 1.76 (m, 2H), 1.76 – 1.67 (m, 1H), 0.94 (d, J = 6.7 Hz, 6H), 0.91 – 0.76 (m, 9H), 0.69 (t, J = 6.4 Hz, 3H). HR-MS m/z: 641.4431 $[M+H]^+$, calcd for $C_{35}H_{56}N_6O_5$: 641.4390.

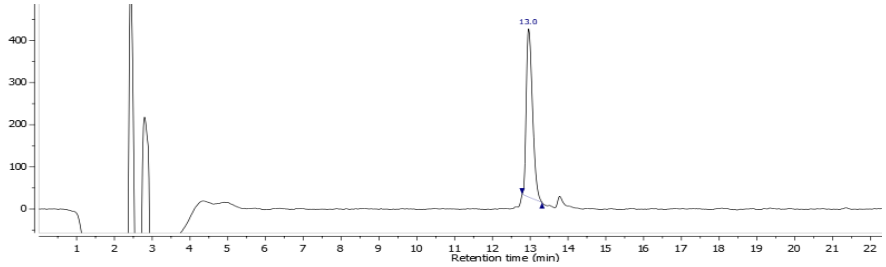

Figure S3. RP-HPLC traces the pure cemadotin.

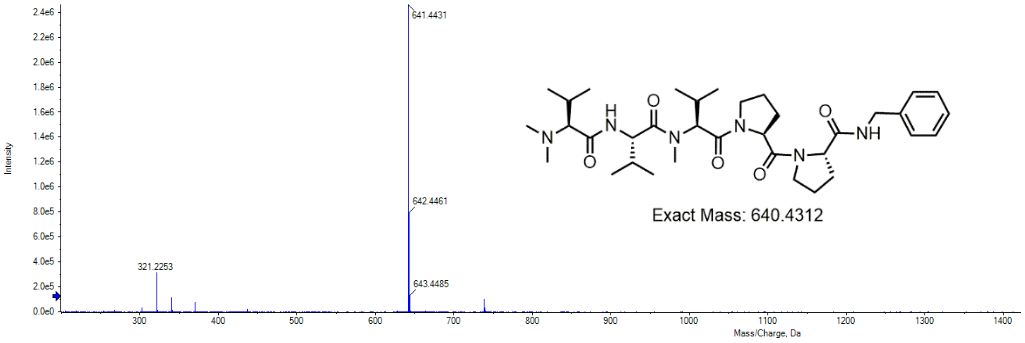

Figure S4. ESI-HRMS of cemadotin.

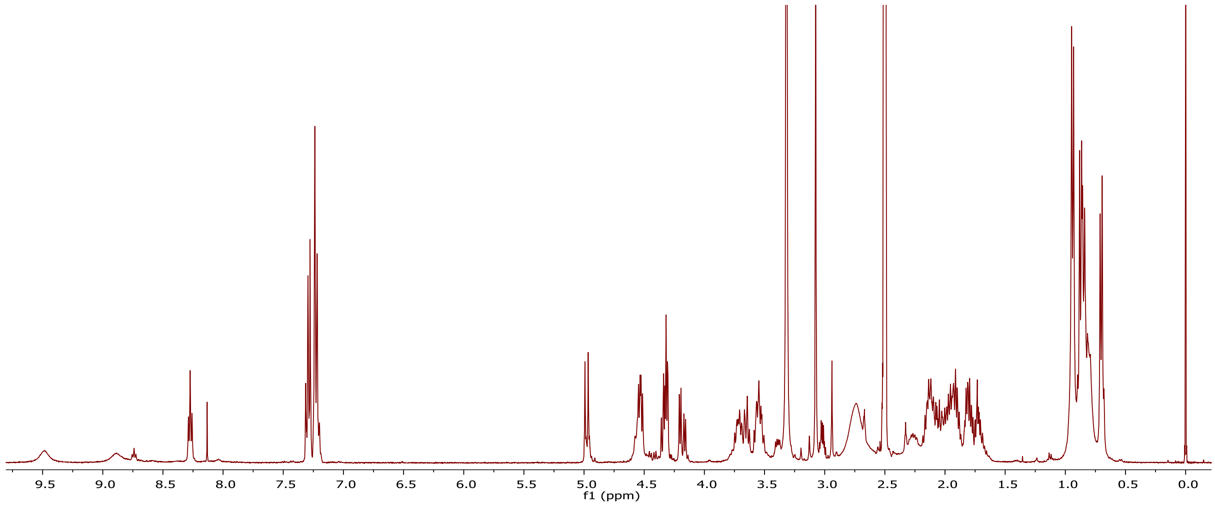

Figure S5. ^{1}H NMR spectrum of cemadotin in DMSO-*d*6.

General procedure for the multicomponent synthesis of dolastatin 15 analogs

A suspension of paraformaldehyde (7 mg, 0.22 mmol, 2 equiv) and the amine (0.22 mmol, 2 equiv.) in 2 mL of MeOH/$CHCl_3$ 4:1 (*v/v*) was stirred for 2 h at room temperature. The pentapeptide acid **1** (73 mg, 0.11 mmol) and the isocyanide (0.13 mmol, 1.2 equiv.) then, were added and the reaction mixture was stirred under microwave irradiation at 60 °C for 4 h. The product formation was checked by TLC and ESI-MS and then the volatiles were removed in a rotavap under reduced pressure. The resulting crude product was purified by preparative RP-HPLC using increasing gradients of acetonitrile or methanol in water to obtain the pure dolastatin analogs. The purity was checked by analytical RP-HPLC.

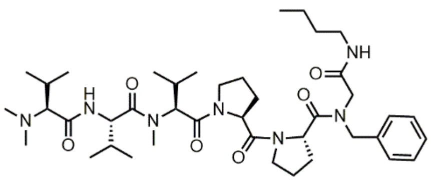

Benzylamine (23.5 mg, 0.22 mmol), paraformaldehyde (7 mg, 0.22 mmol), peptide **1** (73 mg, 0.11 mmol) and *n*-butyl isocyanide (13.8 µL, 10.9 mg, 0.13 mmol) were reacted according to the general procedure described above. HPLC purification rendered the pure dolastatin analog **5a** as an amorphous white solid (56.4 mg, 68% isolated yield, 98 % purity, FA salt). R_t = 16.1 min. ^{1}H NMR (600 MHz, DMSO-d_6): mixture of rotamers, δ 8.25 (s, 1H), 8.07 (d, *J* = 8.4 Hz, 1H), 8.01; 7.45 (t, *J* = 5.7 Hz, 1H), 7.41; 7.18 (d, *J* = 6.9 Hz, 2H), 7.32 – 7.26 (m, 3H), 7.23 (t, *J* = 7.2 Hz, 1H), 4.99 (d, *J* = 10.9 Hz, 1H), 4.84 – 4.78 (m, 2H), 4.66 (dd, *J* = 8.2, 4.9 Hz, 1H), 4.64 (d, *J* = 15.2 Hz, 1H), 4.61 – 4.56 (m, 1H), 4.51 (d, *J* = 8.7 Hz, 1H), 4.47 (d, *J* = 16.5 Hz, 1H), 4.28 (d, *J* = 15.2 Hz, 1H), 4.07; 3.91 (d, *J* = 17.5 Hz, 2H), 3.79 – 3.69 (m, 4H), 3.57 – 3.45 (m, 3H), 3.06 (s, 4H), 3.01 – 2.94 (m, 1H), 2.94 – 2.87 (m, 1H), 2.63 (d, *J* = 10.1 Hz, 1H), 2.19 (s, 6H), 2.16 – 2.08 (m, 3H), 2.08 – 1.99 (m, 1H), 1.99 – 1.92 (m, 2H), 1.92 – 1.85 (m, 2H), 1.83 – 1.69 (m, 3H), 1.41 – 1.29 (m,

2H), 1.23 (m, 2H), 0.93 (t, J = 6.2 Hz, 3H), 0.89 – 0.79 (m, 12H), 0.69 (d, J = 6.6 Hz, 6H). ^{13}C NMR (150 MHz, DMSO): δ 172.7, 172.3, 172.1, 170.0, 169.5, 167.7, 167.6, 167.3, 137.1, 137.0, 128.4, 128.3, 128.2, 127.5, 127.4, 127.3, 126.9, 72.7, 58.2, 57.5, 56.5, 56.1, 53.8, 51.4, 50.0, 49.4, 48.8, 48.5, 47.1, 46.7, 42.5, 41.3, 40.0, 38.2, 31.0, 30.9, 30.1, 29.7, 28.5, 28.3, 27.6, 27.5, 26.7, 26.5, 24.8, 24.3, 24.1, 19.6, 19.5, 19.4, 19.1, 19.0, 18.9, 18.8, 18.7, 18.6, 18.1, 13.6. ESI-MS m/z: 754.5255 [M+H]$^+$, calcd. for $C_{41}H_{68}N_7O_6^+$: 754.5153.

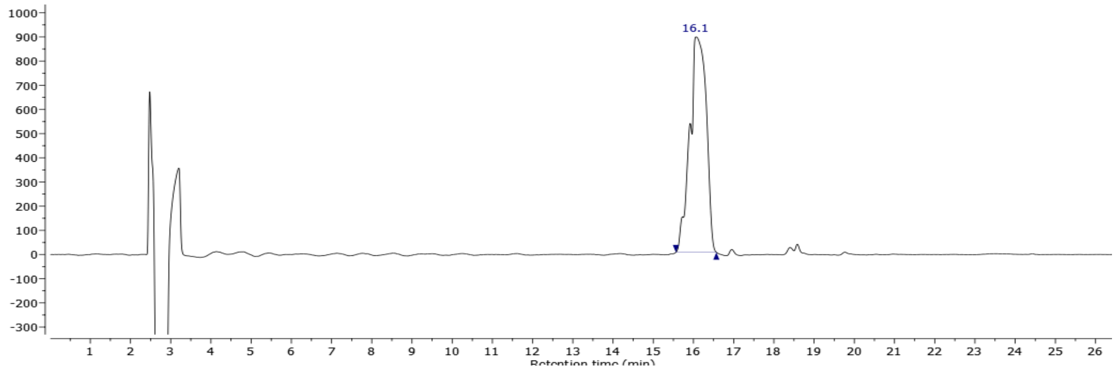

Figure S6. RP-HPLC chromatogram of pure dolastatin analog **5a.**

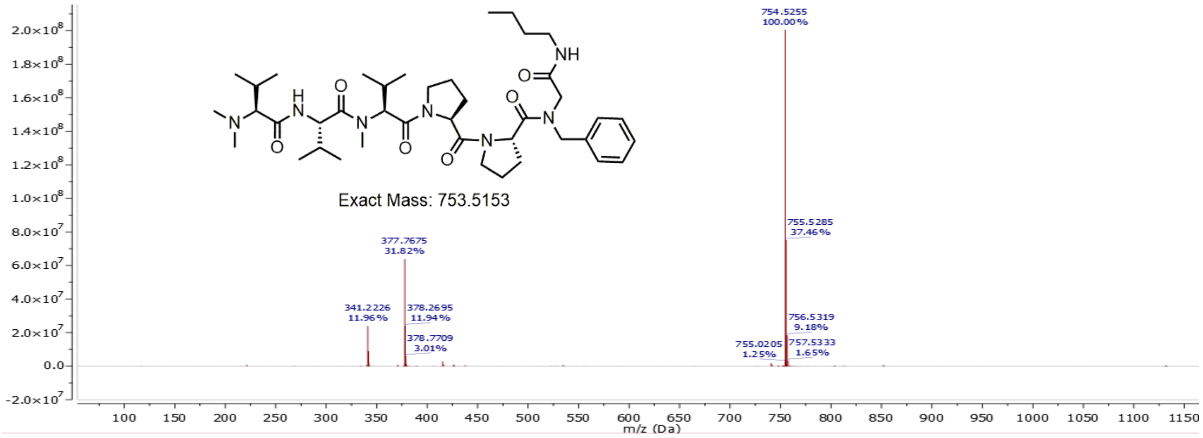

Figure S7. ESI-HRMS of dolastatin analog **5a.**

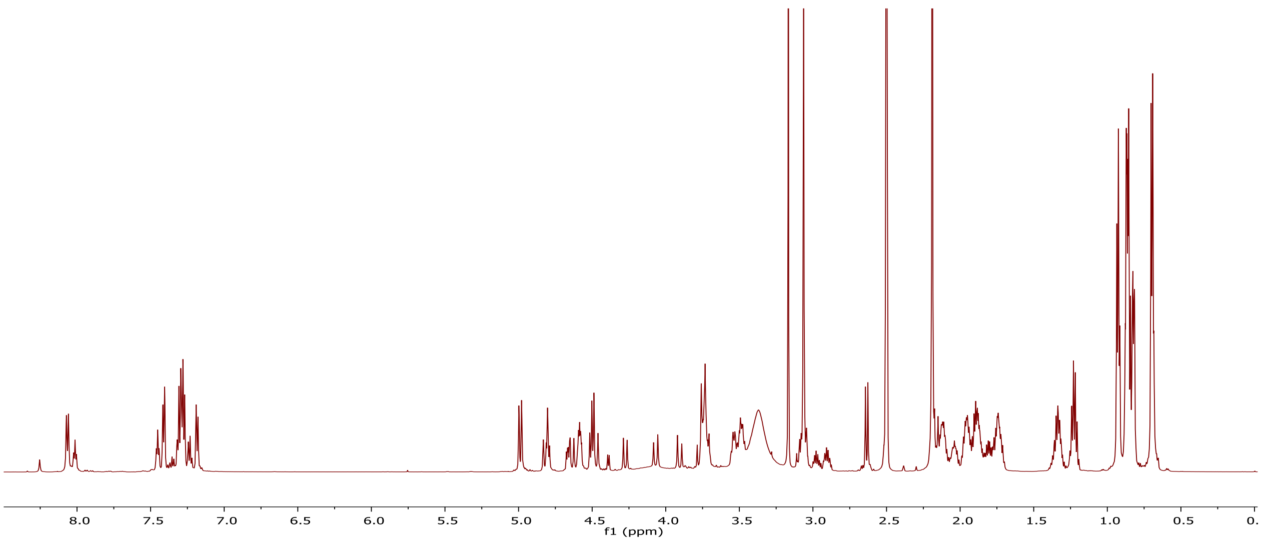

Figure S8. ^{1}H NMR spectrum in DMSO-d_6 of dolastatin analog **5a**.

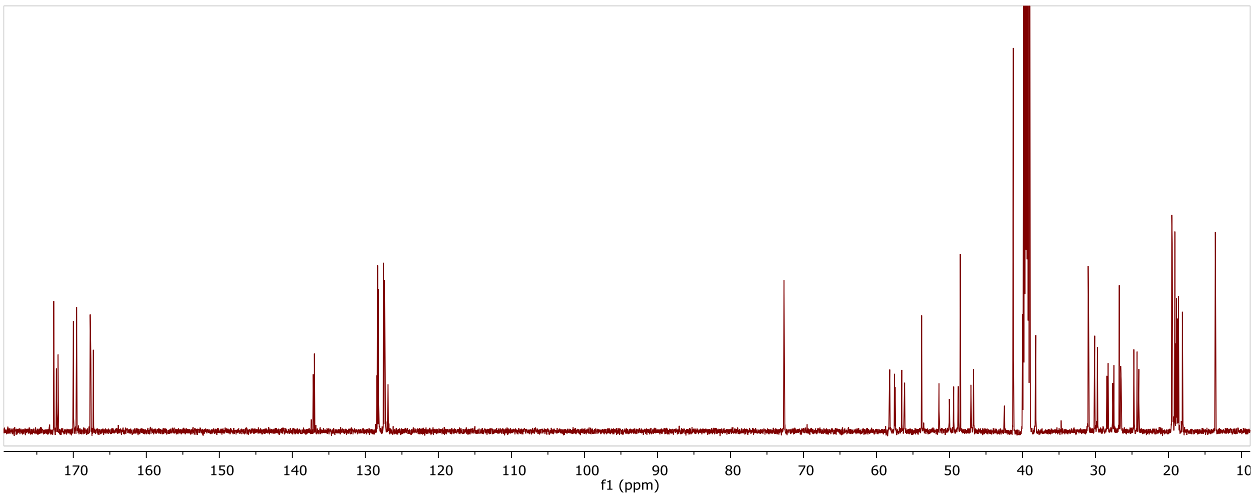

Figure S9. ^{13}C NMR spectrum in DMSO-d_6 of dolastatin analog **5a.**

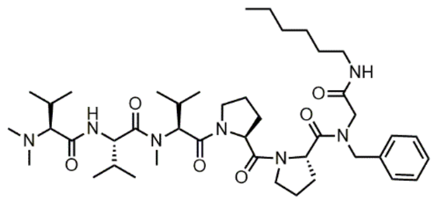

Benzylamine (23.5 mg, 0.22 mmol), paraformaldehyde (7 mg, 0.22 mmol), peptide **1** (73 mg, 0.11 mmol) and n-hexyl isocyanide (16.0 µL, 14.4 mg, 0.13 mmol) were reacted according to the general procedure described above. HPLC purification rendered the pure dolastatin analog **5b** as an amorphous white solid (50.7 mg, 59% isolated yield, 95% purity, FA salt). R_t = 19.8 min. ^{1}H NMR (600 MHz, DMSO-d_6) : mixture of rotamers, δ 8.25 (s, 1H), 8.08 (d, J = 8.3 Hz, 1H), 8.03; 7.46 (t, J = 5.7 Hz, 1H), 7.42 (d, J = 7.1 Hz, 1H), 7.30 (m, 2H), 7.24 (t, J = 7.1 Hz, 1H), 7.19 (d, J = 7.5 Hz, 1H), 5.00 (dd, J = 10.9, 3.3 Hz, 1H), 4.82 (dd, J = 16.9, 12.7 Hz, 2H), 4.67 (dd, J = 8.1, 4.9 Hz, 1H), 4.64; 4.28 (d, J = 15.3 Hz, 1H), 4.60 (dd, J = 8.6, 4.8 Hz, 1H), 4.53 – 4.46 (m, 2H), 4.08 (br.s), 3.92 (d, J = 17.4 Hz, 1H), 3.88 (d, J = 9.7 Hz, 1H), 3.79 – 3.70 (m, 2H), 3.58 – 3.46 (m, 3H), 3.07 (s, 4H), 2.99-2.86 (m, 1H), 2.64 (d, J = 10.1 Hz, 1H), 2.62; 2.39 (t, J = 1.9 Hz, 2H), 2.20 (s, 6H), 2.17 – 2.10 (m, 2H), 2.08 – 2.01 (m, 2H), 2.01 – 1.93 (m, 2H), 1.94 – 1.86 (m, 2H), 1.85 – 1.70 (m, 3H), 1.41 – 1.31 (m, 3H), 1.31 – 1.18 (m, 6H), 0.93 (t, J = 6.6 Hz, 3H), 0.90 – 0.80 (m, 9H), 0.71 (d, J = 6.3 Hz, 3H). ^{13}C NMR (150 MHz, DMSO) δ 172.75, 172.4, 172.2, 170.1, 169.6, 167.8, 167.65, 167.3, 163.7, 137.2, 137.05, 128.9, 128.4, 128.3, 128.2, 127.85, 127.6, 127.5, 127.4, 127.0, 127.8, 72.7, 59.3, 59.1, 58.3, 57.6, 57.5, 56.6, 56.2, 53.9, 51.5, 50.1, 49.5, 48.9, 48.6, 47.1, 46.8, 41.3, 40.0, 38.6, 31.0, 30.95, 30.2, 29.8, 28.9, 28.5, 28.3, 27.7, 27.5, 26.8, 26.6, 26.1, 26.0, 24.8, 24.4, 24.15, 22.1, 22.0, 19.6, 19.2, 19.1, 19.0, 18.8, 18.7, 18.2, 13.9. ESI-MS m/z: 782.5555 [M+H]$^+$, calcd. for $C_{43}H_{72}N_7O_6^+$: 782.5466.

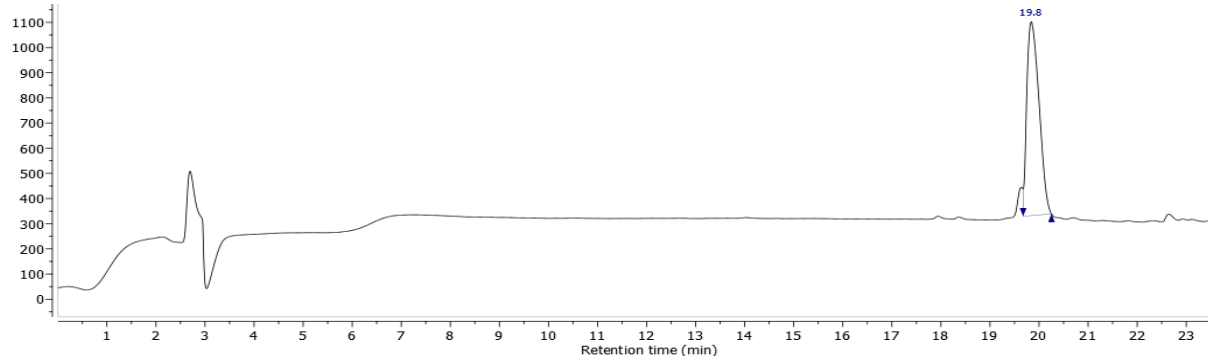

Figure S10. RP-HPLC chromatogram of pure dolastatin analog **5b**.

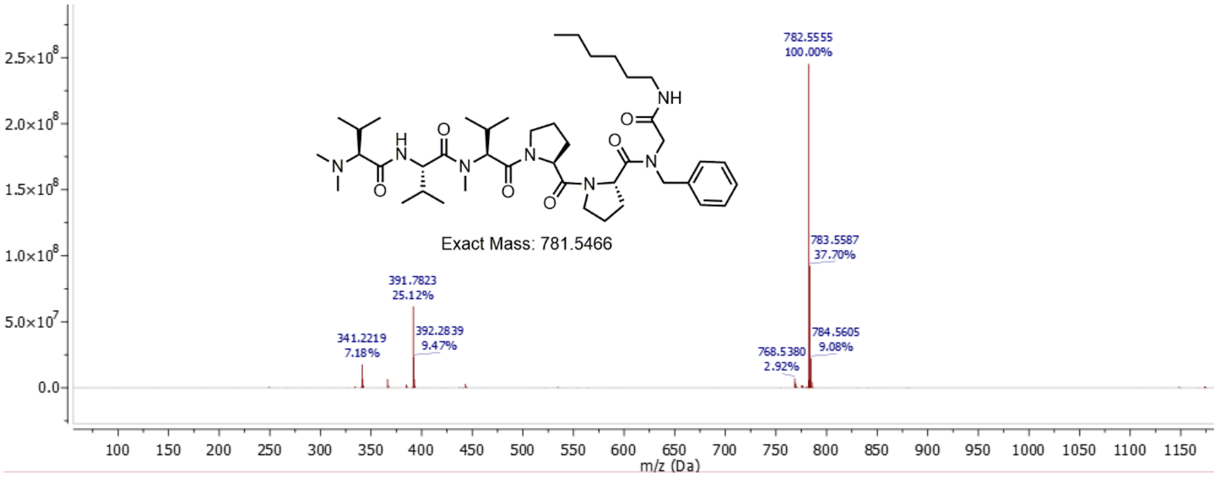

Figure S11. ESI-HRMS dolastatin analog **5b**.

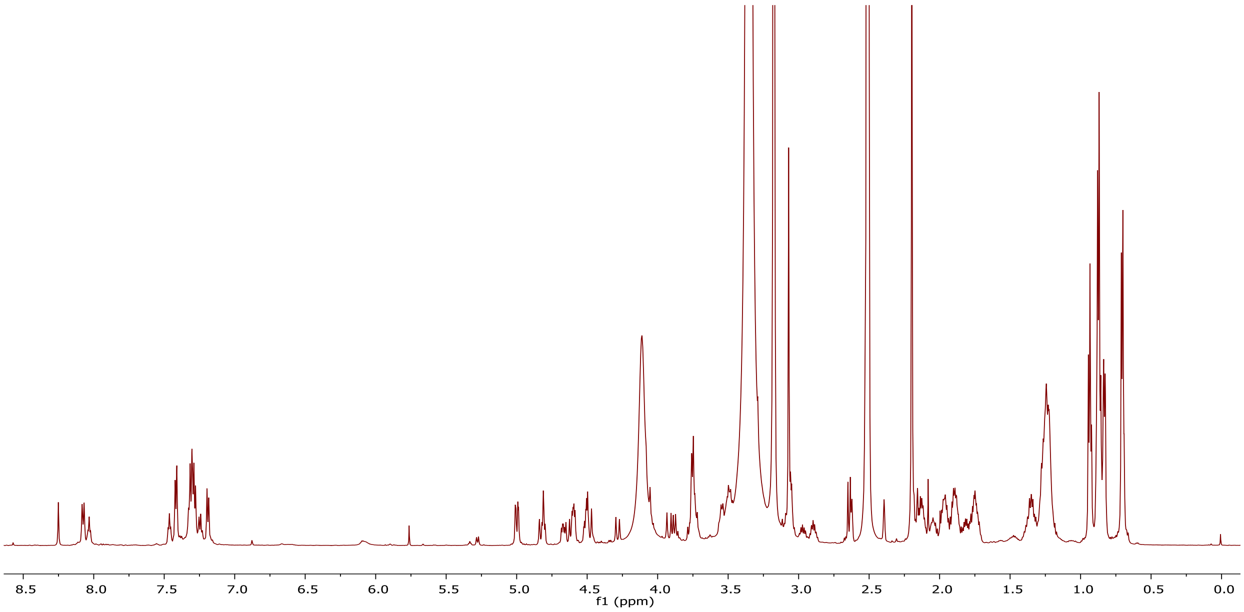

Figure S12. ^{1}H NMR spectrum in DMSO-d_6 of dolastatin analog **5b**.

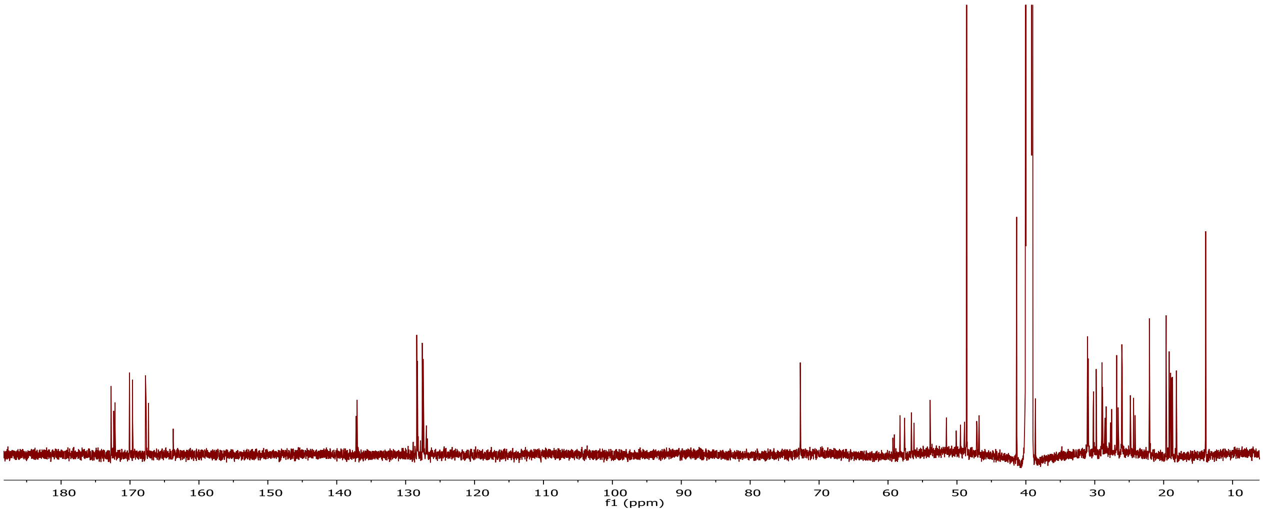

Figure S13. ^{13}C NMR spectrum in DMSO-d_6 of dolastatin analog **5b**.

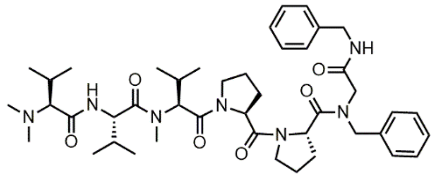

Benzylamine (23.5 mg, 0.22 mmol), paraformaldehyde (7 mg, 0.22 mmol), peptide **1** (73 mg, 0.11 mmol) and benzylisocyanide (16.4 µL, 17.5 mg, 0.13 mmol) were reacted according to the general procedure described above. HPLC purification rendered the pure dolastatin analog **5c** as an amorphous white solid (47.6 mg, 55% isolated yield, 96% purity, FA salt). R_t = 15.7 min. ^{1}H NMR (600 MHz, DMSO-d_6) : mixture of rotamers, δ 8.58 (t, *J* = 6.0 Hz, 1H), 8.23 (s, 1H), 8.15 (t, *J* = 6.1 Hz, 1H), 8.08-8.03 (m, 1H), 7.44 (d, *J* = 7.0 Hz, 1H), 7.35 – 7.27 (m, 5H), 7.22 (dd, *J* = 18.3, 7.3 Hz, 4H), 4.99; 4.94 (d, *J* = 10.9/11.0 Hz, 1H), 4.89 (d, *J* = 16.6 Hz, 1H), 4.82; 4.71 (dd/dd, *J* = 8.2/7.6, 5.6/4.7 Hz, 1H), 4.75 (d, *J* = 15.4 Hz, 1H), 4.60; 4.55 (dd/dd, *J* = 8.5/8.6, 4.6/5.7 Hz, 1H), 4.54 – 4.49 (m, 2H), 4.34 (dd, *J* = 15.1, 6.1 Hz, 1H), 4.24 (m, 3H), 3.94 (dd, *J* = 16.9, 9.1 Hz, 2H), 3.78 – 3.66 (m, 3H), 3.57 – 3.46 (m, 2H), 3.27 (dd, *J* = 9.9, 6.4 Hz, 1H), 3.07 (d, *J* = 7.7 Hz, 3H), 2.64 (d, *J* = 10.0 Hz, 1H), 2.20 (s, 6H), 2.15 – 2.02 (m, 3H), 2.00 – 1.84 (m, 4H), 1.82 – 1.59 (m, 4H), 0.93 (t, *J* = 6.4 Hz, 3H), 0.91 – 0.78 (m, 9H), 0.73 – 0.65 (m, 6H). ^{13}C NMR (150 MHz, DMSO) δ 172.7, 172.5, 172.2, 170.0, 169.6, 168.1, 167.8, 167.7, 163.7, 139.2, 139.0, 137.2, 137.1, 128.5, 128.4, 128.3, 128.2, 127.55, 127.4, 127.3, 127.25, 126.95, 126.8, 126.6, 72.7, 58.3, 58.2, 57.6, 56.6, 56.15, 53.9, 51.5, 49.9, 49.6, 48.75, 48.6, 47.0, 46.7, 42.25, 41.9, 41.3, 30.2, 29.8, 28.6, 28.3, 27.7, 27.5, 26.8, 26.6, 26.55, 24.8, 24.4, 24.1, 19.6, 19.2, 19.1, 19.0, 18.7, 18.2, 18.1. ESI-MS m/z: 788.5103 [M+H]$^{+}$, calcd. for $C_{44}H_{66}N_7O_6^{+}$: 788.4996.

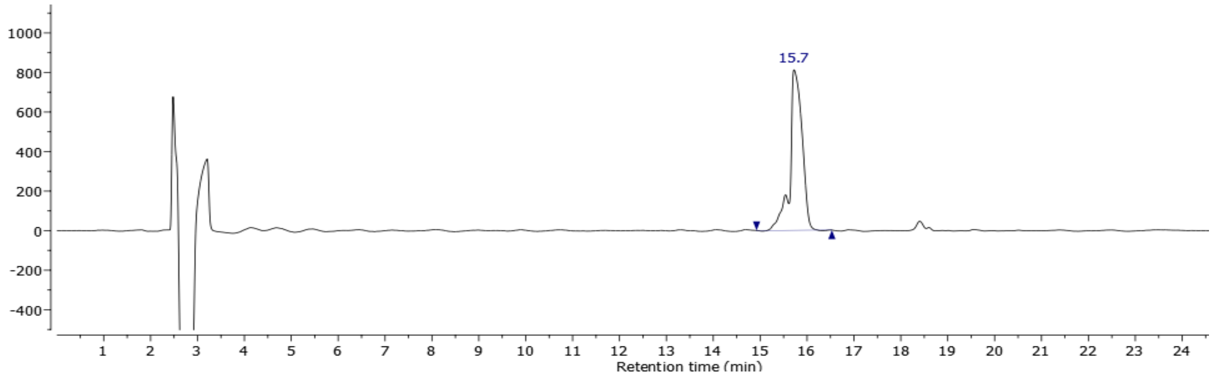

Figure S14. RP-HPLC chromatogram of pure dolastatin analog **5c.**

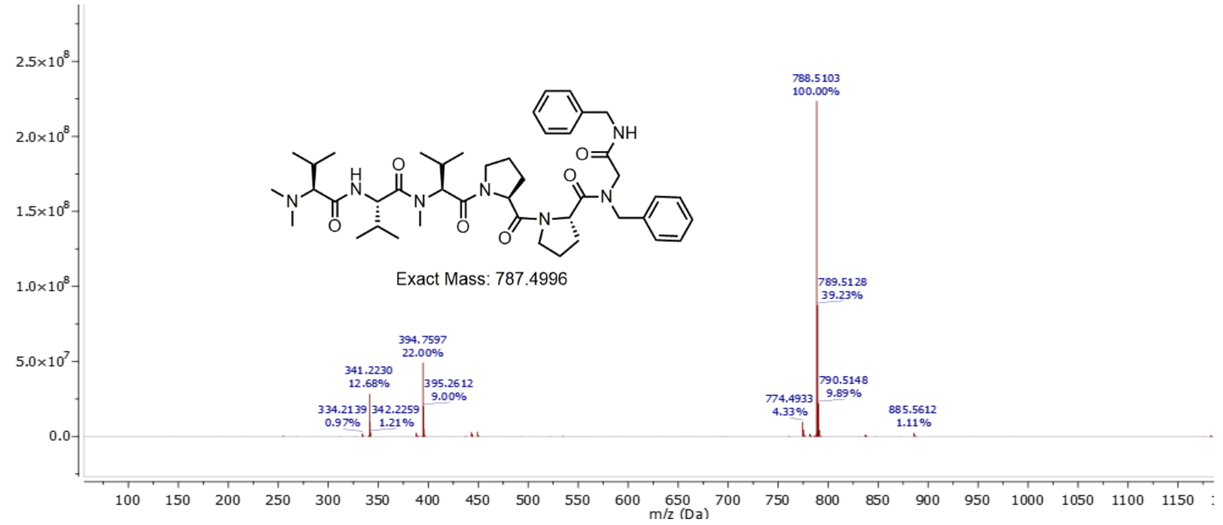

Figure S15. ESI-HRMS of dolastatin analog **5c.**

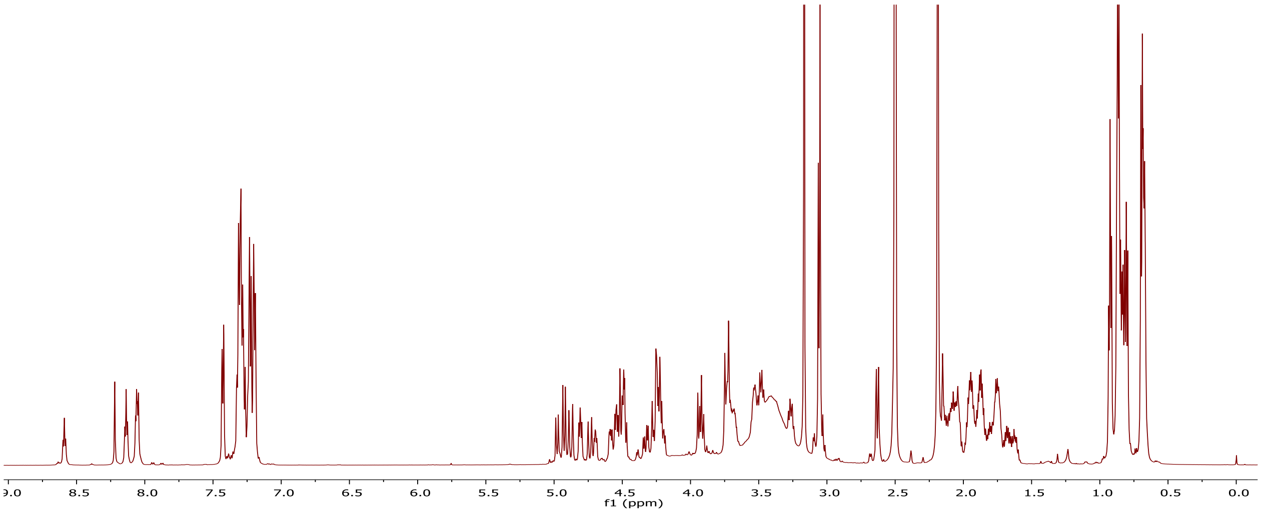

Figure S16. ^{1}H NMR spectrum in DMSO-d_6 of dolastatin analog **5c.**

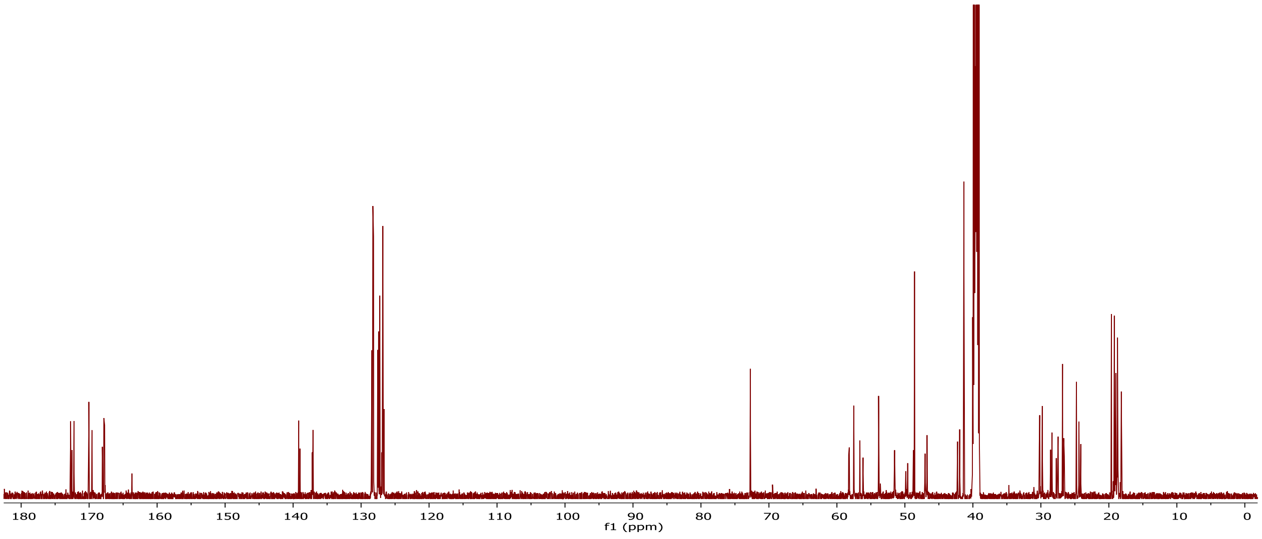

Figure S17. ^{13}C NMR spectrum in DMSO-d_6 of dolastatin analog **5c.**

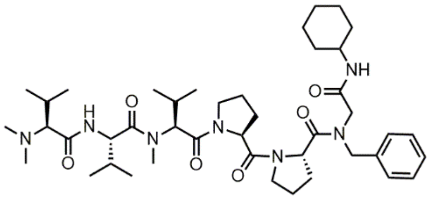

Benzylamine (23.5 mg, 0.22 mmol), paraformaldehyde (7 mg, 0.22 mmol), peptide **1** (73 mg, 0.11 mmol) and cyclohexyl isocyanide (16.4 µL, 14 mg, 0.13 mmol) were reacted according to the general procedure described above. HPLC purification rendered the pure dolastatin analog **5d** as an amorphous white solid (54.8 mg, 64 % isolated yield, 95% purity, FA salt). R_t = 16.2 min. ^{1}H NMR (600 MHz, DMSO-d_6) : mixture of rotamers, δ 8.39 (s, 1H), 8.06; 7.84 (d, *J* = 8.4 Hz, 7.8 Hz, 1H), 7.43 – 7.39 (m, 1H), 7.33 – 7.21 (m, 3H), 7.18 (dd, *J* = 7.7, 4.9 Hz, 1H), 4.98 (dd, *J* = 11.0, 1.9 Hz, 1H), 4.83 – 4.78 (m, 2H), 4.71 (d, *J* = 15.4 Hz, 1H), 4.68 (dd, *J* = 8.1, 4.6 Hz, 1H), 4.60-4.56 (m, 1H), 4.53 – 4.45 (m, 2H), 4.22 (d, *J* = 15.5 Hz, 1H), 4.10 (br, 1H), 3.91 – 3.81 (m, 2H), 3.79-3.69 (m, 2H), 3.66 (d, *J* = 16.4 Hz, 1H), 3.57 – 3.46 (m, 2H), 3.08 – 3.03 (m, 3H), 2.63 (d, *J* = 10.1 Hz, 1H), 2.61; 2.39 (t, *J* = 1.9 Hz, 1H), 2.19 (s, 7H), 2.13–1.99 (m, 4H), 1.99 – 1.49 (m, 14H), 1.23 (brs, 3H), 1.17 – 1.03 (m, 3H), 0.93 (d, *J* = 6.4 Hz, 6H), 0.87 (d, *J* = 6.3 Hz, 6H), 0.82 (d, *J* = 6.7 Hz, 3H), 0.71 (d, *J* = 6.4 Hz, 6H). ^{13}C NMR (150 MHz, DMSO) δ 172.7, 172.5, 172.1, 170.05, 169.55, 169.5, 167.7, 166.8, 166.4, 137.3, 137.0, 128.4, 128.3, 127.7, 127.45, 126.9, 72.7, 58.3, 57.7, 57.5, 56.6, 56.1 53.9, 51.6, 49.6, 49.1, 48.6, 47.75, 47.6, 47.1, 46.8, 41.3, 32.3, 32.2, 32.1, 30.2, 29.8, 28.6, 28.4, 27.8, 27.6, 26.8, 26.6, 26.5, 25.2, 25.1, 24.9, 24.7, 24.6, 24.5, 24.1, 19.6, 19.2, 19.15, 19.0, 18.9, 18.7, 18.2, 18.1. ESI-MS m/z: 780.5400 $[M+H]^+$, calcd. for $C_{43}H_{70}N_7O_6^+$: 780.5309.

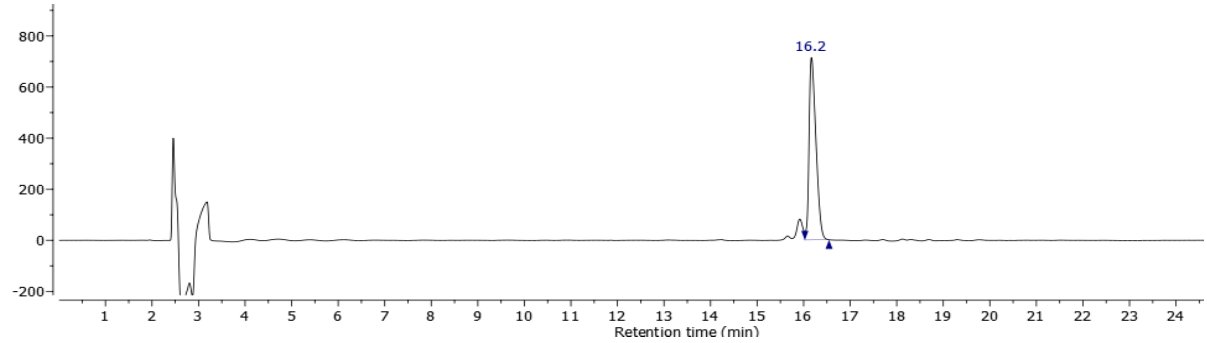

Figure S18. RP-HPLC chromatogram of pure dolastatin analog **5d.**

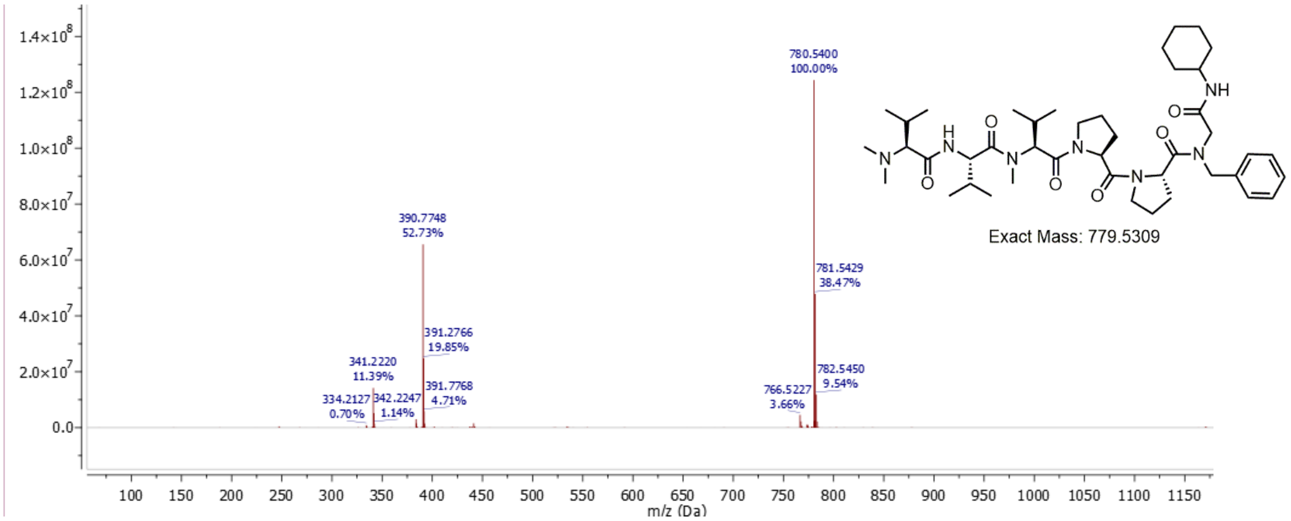

Figure S19. ESI-HRMS of dolastatin analog **5d.**

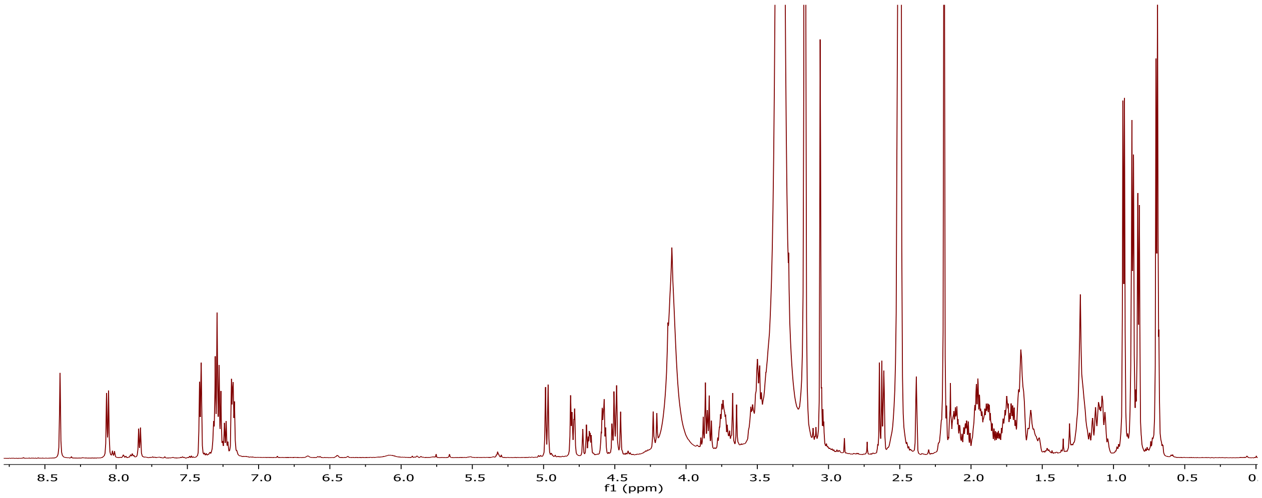

Figure S20. ^{1}H NMR spectrum in DMSO-d_6 of dolastatin analog **5d.**

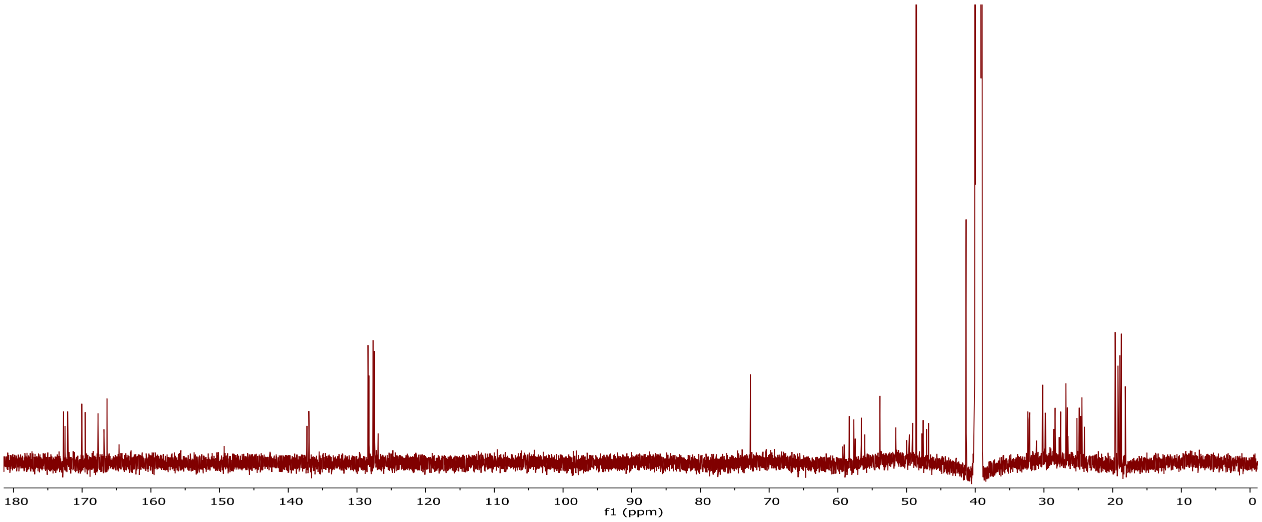

Figure S21. ^{13}C NMR spectrum in DMSO-d_6 of dolastatin analog **5d.**

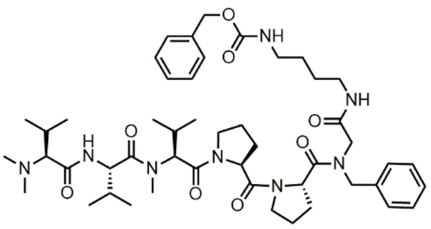

Benzylamine (23.5 mg, 0.22 mmol), paraformaldehyde (7 mg, 0.22 mmol), peptide **1** (73 mg, 0.11 mmol) and benzyl (4-isocyanobutyl) carbamate (30.5 mg, 0.13 mmol) were reacted according to the general procedure described above. HPLC purification rendered the pure dolastatin analog **5e** as an amorphous white solid (62.5 mg, 63% isolated yield, 97% purity, FA salt). R_t = 16.8 min. ^{1}H NMR (600 MHz, DMSO-d_6) : mixture of rotamers, δ 8.17 (s, 1H), 8.08 (d, *J* = 8.6 Hz, 1H)**,** 8.05; 7.54 (t, *J* = 5.8 Hz, 1H), 7.41 (d, *J* = 7.4 Hz, 2H), 7.38 – 7.26 (m, 7H), 7.25 – 7.16 (m, 2H), 5.03 – 4.96 (m, 1H), 4.85 – 4.77 (m, 2H), 4.68 – 4.63 (m, 2H), 4.60-4.54 (m, 2H), 4.52-4.48 (m, 2H), 4.45 (d, *J* = 16.5 Hz, 1H), 4.26 (d, *J* = 15.1 Hz, 1H), 4.08; 3.88 (d, *J* = 17.3/17.7 Hz, 1H), 3.82; 3.64 (d, *J* = 16.3 Hz, 1H), 3.78 – 3.69 (m, 3H), 3.57 – 3.45 (m, 4H), 3.11 – 3.02 (m, 4H), 3.01 – 2.91 (m, 4H), 2.63 (d, *J* = 10.0 Hz, 1H), 2.19 (s, 6H), 2.14-2.07 (m, 2H), 2.03 (m, 1H), 2.00 – 1.84 (m, 5H), 1.80 – 1.67 (m, 2H), 1.35 (d, *J* = 3.2 Hz, 4H), 0.92 (t, *J* = 7.1 Hz, 6H), 0.87 (t, *J* = 5.8 Hz, 6H), 0.82 (dd, *J* = 6.7, 2.7 Hz, 3H), 0.70 (d, *J* = 6.4 Hz, 6H). ^{13}C NMR (150 MHz, DMSO) δ 172.8, 172.4, 172.1, 170.05, 169.6, 167.8, 167.7, 167.4, 163.3, 156.1, 137.3, 137.2, 137.05, 128.4, 128.3, 127.7, 127.6, 127.55, 127.4, 127.0, 72.7, 65.1, 58.3, 58.2, 57.6, 57.5, 56.6, 56.2, 53.9, 51.4, 50.0, 49.5, 48.6, 47.2, 47.1, 46.8, 41.3, 40.0, 39.9, 38.3, 38.2, 30.2, 29.8, 28.5, 28.3, 27.7, 27.6, 26.8, 26.7, 26.6, 26.3, 24.8, 24.4, 24.1, 19.6, 19.2, 19.0, 18.75, 18.7, 18.2, 18.1.ESI-MS m/z: 903.5711 $[M+H]^+$, calcd. for $C_{49}H_{75}N_8O_8^+$: 903.5630.

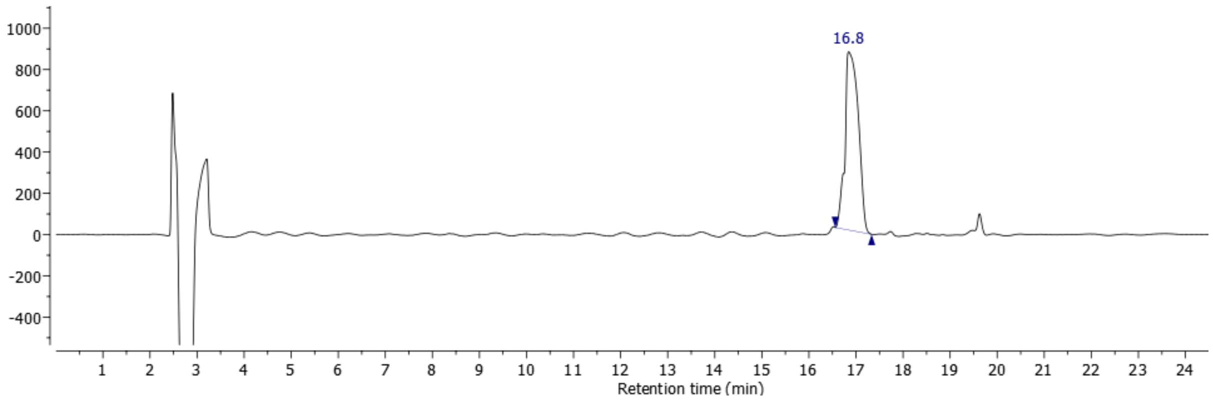

Figure S22. RP-HPLC chromatogram of pure dolastatin analog **5e.**

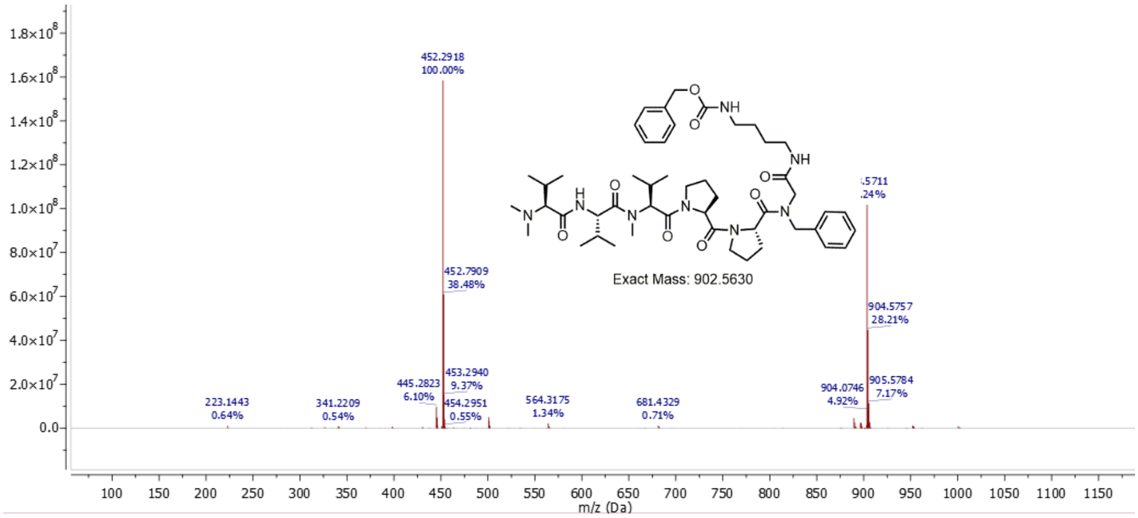

Figure S23. ESI-HRMS of dolastatin analog **5e.**

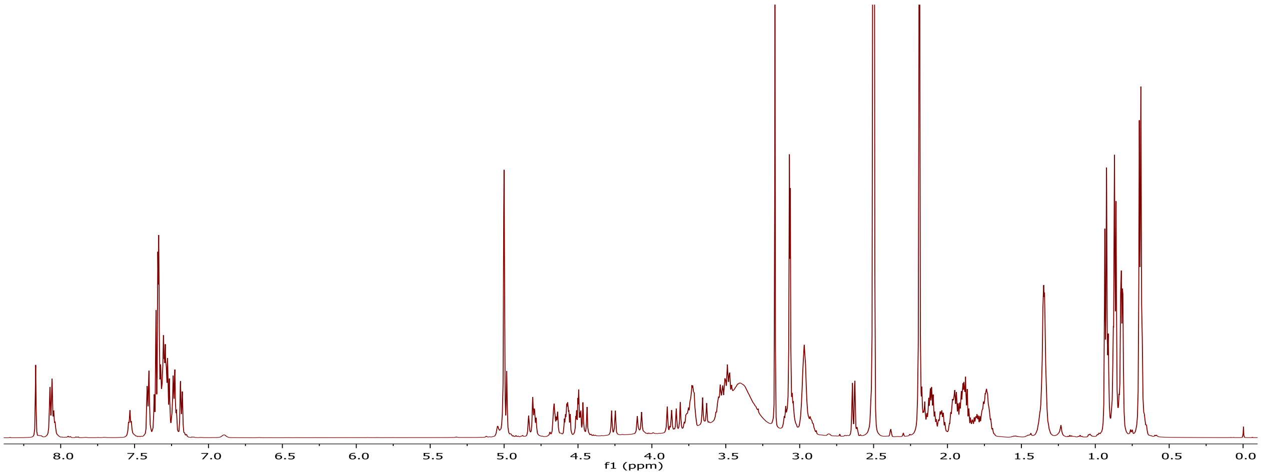

Figure S24. ^{1}H NMR spectrum in DMSO-d_6 of dolastatin analog **5e.**

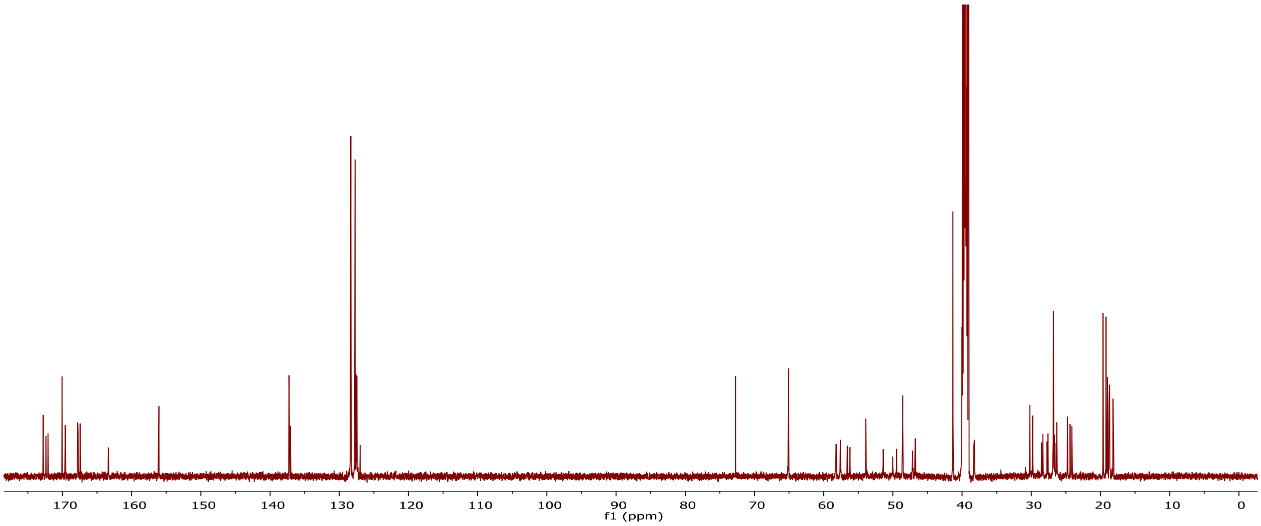

Figure S25. ^{13}C NMR spectrum in DMSO-d_6 of dolastatin analog **5e.**

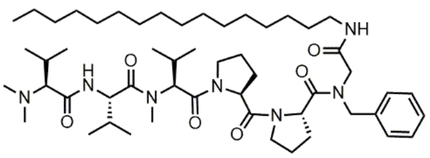

Benzylamine (23.5 mg, 0.22 mmol), paraformaldehyde (7 mg, 0.22 mmol), peptide **1** (73 mg, 0.11 mmol) and *n*-hexadecyl isocyanide (33 mg, 0.13 mmol) were reacted according to the general procedure described above. HPLC purification rendered the pure dolastatin analog **5f** as an amorphous white solid (63.9 mg, 60 % isolated yield, 95% purity). R_t = 19.7 min. ^{1}H NMR (600 MHz DMSO-d_6):): mixture of rotamers, δ 8.07 (d, *J* = 7.9 Hz, 1H), 8.02; 7.43 (t, *J* = 5.7 Hz, 1H), 7.40 (d, *J* = 7.3 Hz, 1H), 7.32 – 7.21 (m, 3H), 7.18 (d, *J* = 7.3 Hz, 1H), 4.99 (d, *J* = 11.2 Hz, 1H), 4.84 – 4.77 (m, 2H), 4.66 (dd, *J* = 8.2, 4.9 Hz, 1H), 4.58 (m, 2H), 4.52-4.47 (m, 2H), 4.30 (d, *J* = 15.1 Hz, 2H), 4.03 (d, *J* = 17.4 Hz, 1H), 3.93 (d, *J* = 17.4 Hz, 1H), 3.79 – 3.69 (m, 3H), 3.56 – 3.44 (m, 2H), 3.17; 3.06 (s, 3H), 2.99 – 2.90 (m, 1H), 2.89-2.83 (m, 1H), 2.63 (d, *J* = 10.1 Hz, 1H), 2.19 (s, 7H), 2.16-2.00 (m, 2H), 2.00-1.92 (m, 2H), 1.92-1.79 (m, 2H), 1.79-1.69 (m, 2H), 1.37 – 1.29 (m, 6H), 1.23 (s, 21H), 0.92 (t, *J* = 6.5 Hz, 6H), 0.89 – 0.80 (m, 9H), 0.69 (d, *J* = 6.1 Hz, 6H). ^{13}C NMR (150 MHz, DMSO) δ 172.7, 172.3, 172.2, 170.05, 169.7, 167.75, 167.6, 167.3, 137.2 137.05, 129.1, 128.4, 128.3, 128.0, 127.6, 127.5, 127.4, 127.0, 125.75, 114.3, 72.7, 58.25, 57.6, 57.5, 56.6, 56.2, 53.9, 51.6, 50.1, 49.5, 49.0, 48.6, 47.1, 46.8, 41.4, 41.3, 38.6, 31.3, 30.2, 29.8, 29.0, 28.8, 28.7, 28.5, 28.3, 28.2, 27.7, 27.5, 26.8, 26.6, 26.4, 26.3, 24.85, 24.35, 24.15, 22.1, 19.6, 19.2, 19.05, 19.0, 18.8, 18.7, 18.15, 18.1, 13.9. ESI-MS m/z: 922.7112 [M+H]$^+$, calcd for $C_{53}H_{92}N_7O_6^+$: 922.7031.

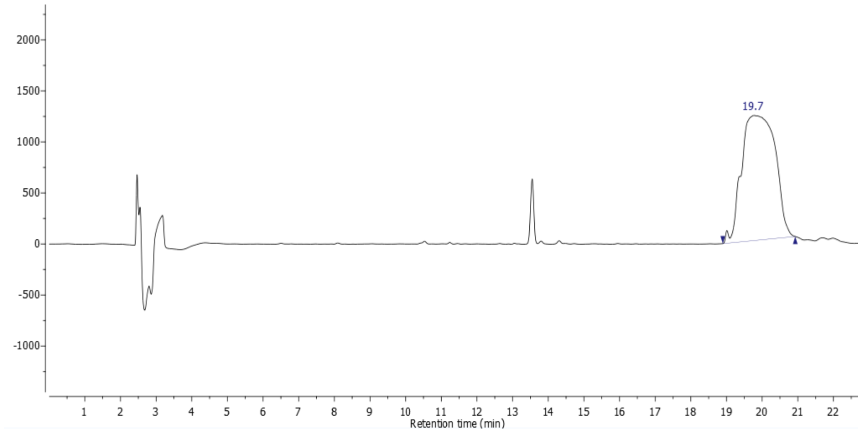

Figure S26. RP-HPLC chromatogram of pure dolastatin analog **5f**.

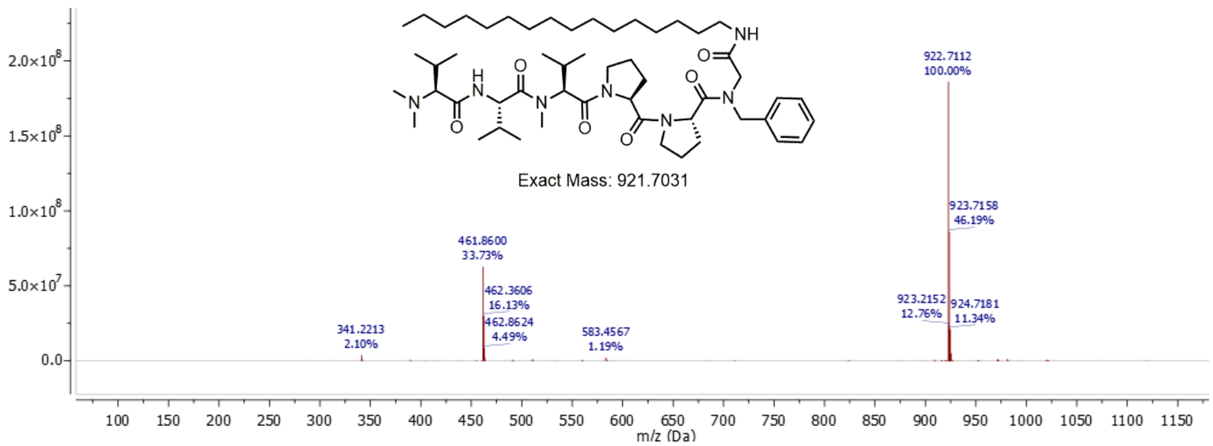

Figure S27. ESI-HRMS of dolastatin analog **5f**.

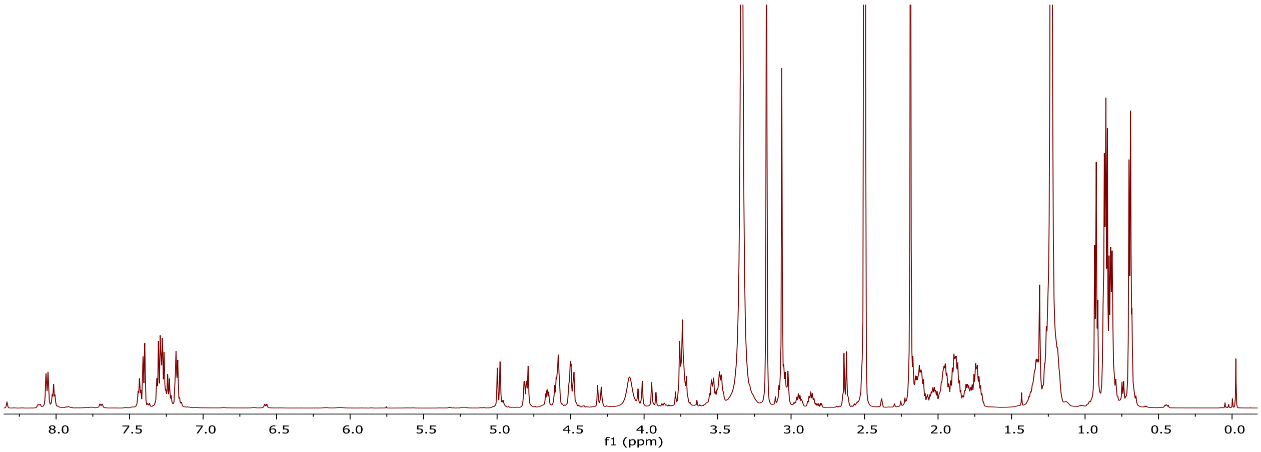

Figure S28. ^{1}H NMR spectrum in DMSO-d_6 of dolastatin analog **5f**.

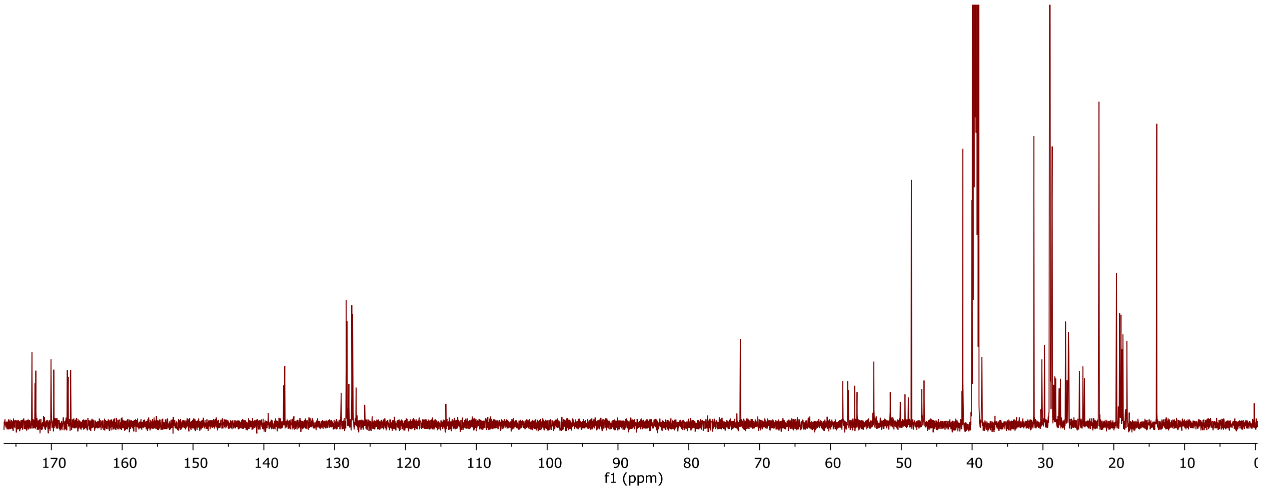

Figure S29. ^{13}C NMR spectrum in DMSO-d_6 of dolastatin analog **5f**.

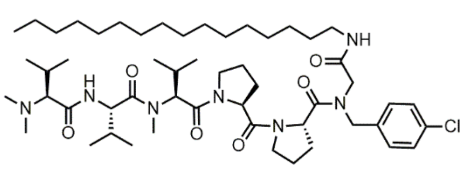

4-chloro benzylamine (31 mg, 0.22 mmol), paraformaldehyde (7 mg, 0.22 mmol), peptide **1** (73 mg, 0.11 mmol) and *n*-hexadecyl isocyanide (33 mg, 0.13 mmol) were reacted according to the general procedure described above. HPLC purification rendered the pure dolastatin analog **5g** as an amorphous white solid (54.6 mg, 52% isolated yield, > 97% purity). R_t = 21.3 min. ^{1}H NMR (600 MHz, CD_3OD) : mixture of rotamers, δ 7.44 (d, *J* = 8.2 Hz, 1H), 7.37 – 7.33 (m, 3H), 7.32 – 7.29 (m, 1H), 7.26 (d, *J* = 8.2 Hz, 1H), 5.13 (dd, *J* = 11.0, 6.8 Hz, 1H), 4.90- 4.81(m, 2H), 4.82; 4.36 (d, *J* = 15.1 Hz, 1H), 4.74 (dd, *J* = 8.1, 5.7 Hz, 1H), 4.69-4.63 (m, 3H), 4.22; 4.15 (d, *J* = 17.8/16.7 Hz, 1H), 4.07 (d, *J* = 17.8 Hz, 1H), 3.95 – 3.86 (m, 2H), 3.84 (d, *J* = 16.8 Hz, 1H), 3.70 – 3.61 (m, 2H), 3.23 – 3.19 (m, 4H), 3.19 – 3.15 (m, 1H), 3.03 (m, 1H), 2.68 (d, *J* = 9.0 Hz, 1H), 2.32 (s, 6H), 2.30 – 1.85 (m, 11H), 1.53 – 1.46 (m, 2H), 1.47 – 1.37 (m, 2H), 1.30 (brs, 28H), 1.04 (t, *J* = 6.0 Hz, 6H), 0.98 (d, *J* = 6.8 Hz, 3H), 0.95 (dd, *J* = 6.8, 3.5 Hz, 3H), 0.91 (t, *J* = 7.0 Hz, 6H), 0.87 – 0.80 (m, 3H). ^{13}C NMR (150 MHz, CD_3OD) δ 175.2, 175.1, 175.0, 174.9, 173.6, 173.3, 172.1, 172.0, 170.55, 170.5, 170.3, 170.2, 139.3, 137.4, 136.7, 136.5, 134.8, 134.5, 134.4, 134.1, 131.1, 130.8, 130.5, 130.4, 129.9, 129.8, 129.7, 129.6, 116.8, 75.7, 60.8, 59.7, 58.6, 58.5, 57.8, 57.6, 57.5, 57.3, 57.2, 56.0, 53.4, 53.2, 51.9, 51.2, 51.1, 49.6, 43.5, 42.4, 40.6, 40.2, 33.1, 31.7, 31.4, 31.3, 30.8, 30.7, 30.5, 30.45, 30.4, 30.3, 30.2, 29.95, 29.4, 29.2, 28.8, 28.5, 28.4, 28.05, 28.0, 26.35, 26.0, 25.8, 23.75, 20.2, 19.9, 19.8, 19.6, 19.5, 19.3, 19.25, 19.1, 14.45. ESI-MS m/z: 479.3468 $[M+2H]^{2+}$, calcd for $C_{53}H_{92}ClN_7O_6^{2+}$: 479.347.

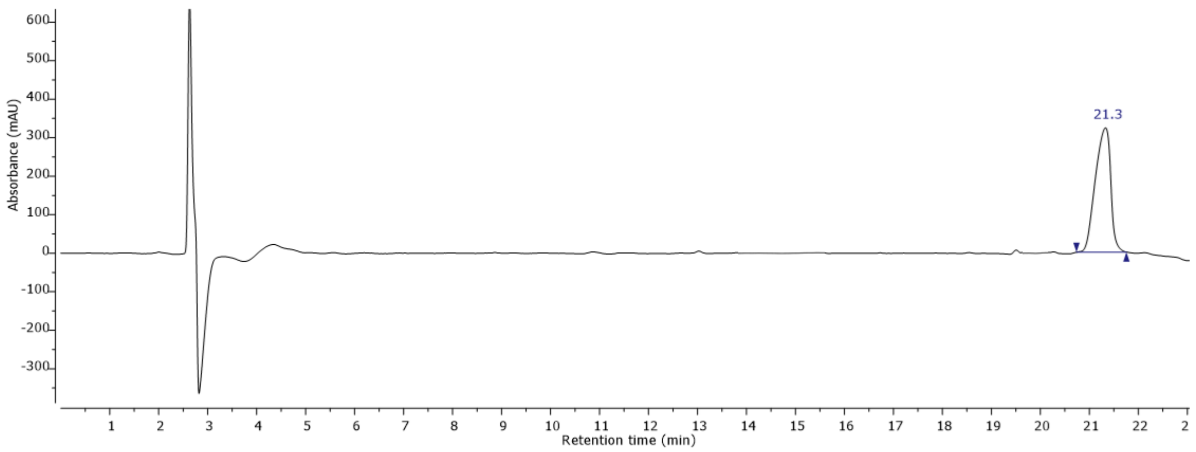

Figure S30. RP-HPLC chromatogram of pure dolastatin analog **5g**.

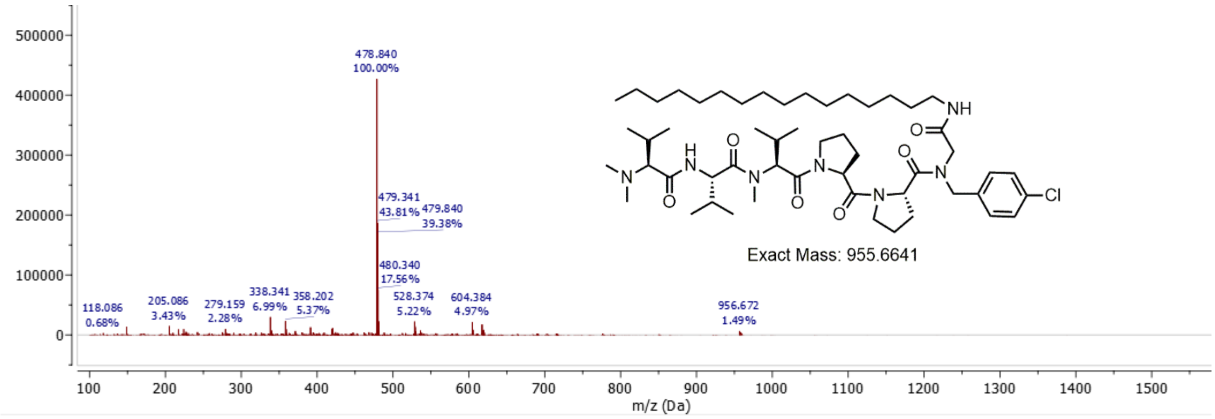

Figure S31. ESI-HRMS dolastatin analog **5g**.

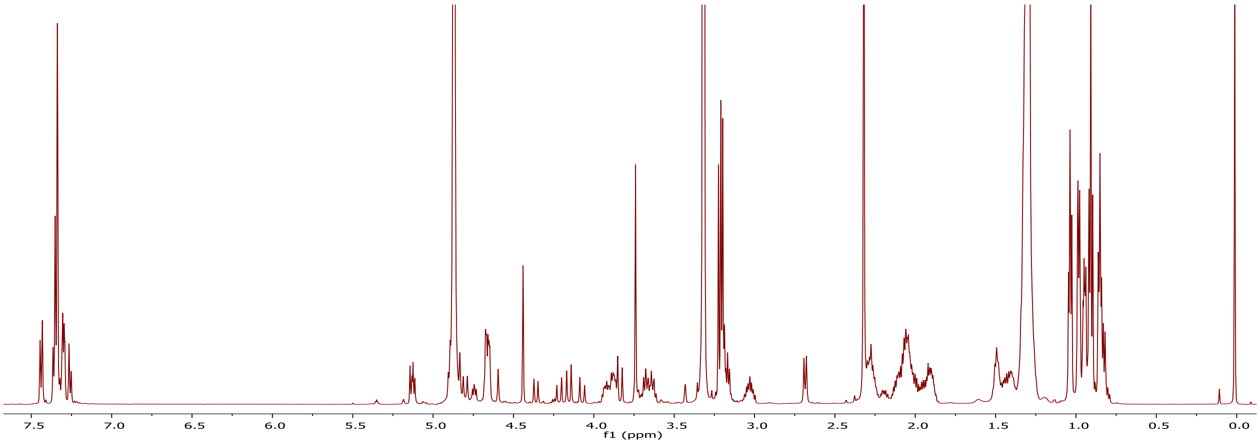

Figure S32. ^{1}H NMR spectrum in CD_3OD of dolastatin analog **5g**.

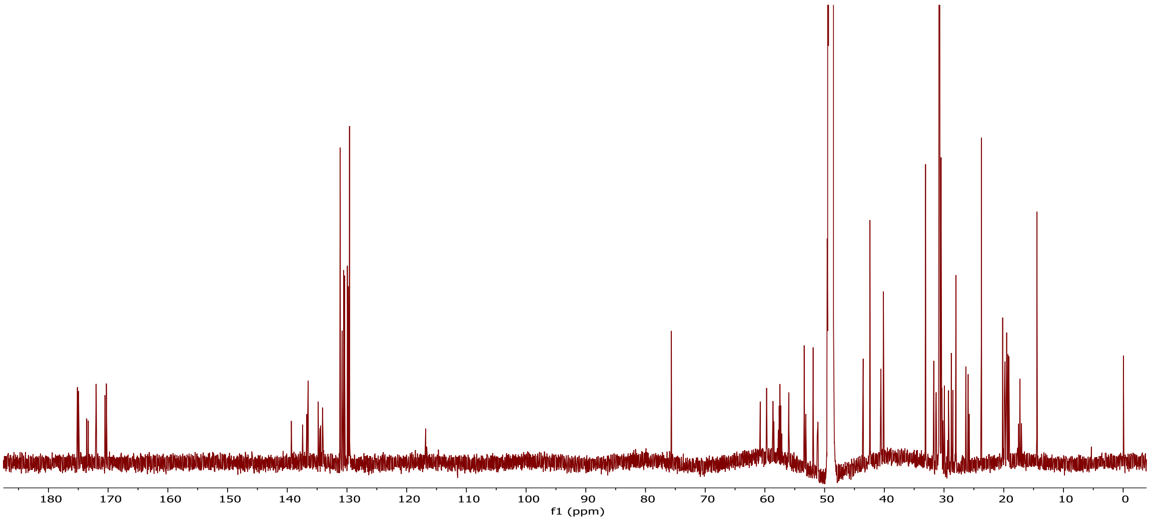

Figure S33. ^{13}C NMR spectrum in CD_3OD of dolastatin analog **5g**.

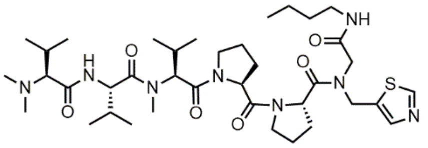

Thiazol-5-ylmethanamine-HCl (41 mg, 0.22 mmol), paraformaldehyde (7 mg, 0.22 mmol), triethylamine (30 µL, 0.22 mmol), peptide **1** (73 mg, 0.11 mmol) and *n*-butyl isocyanide (13.8 µL, 10.9 mg, 0.13 mmol) were reacted according to the general procedure described above. HPLC purification rendered the pure dolastatin analog **5h** as an amorphous yellow solid (55.2 mg, 66% isolated yield, 95% purity). R_t = 11.4 min. ^{1}H NMR (600 MHz, CD_3OD): mixture of rotamers, δ 9.00 (s, 1H), 8.93 (s, 1H), 8.42 (s, 3H), 7.96 (s, 1H), 7.83 (s, 1H), 5.15 – 5.07 (m, 2H), 5.02 – 4.95 (m, 1H), 4.72 – 4.62 (m, 6H), 4.29 – 4.12 (m, 3H), 4.25 (d, *J* = 17.6 Hz, 1H), 4.18 (dd, *J* = 17.5, 16.6 Hz, 2H), 3.73 – 3.61 (m, 4H), 3.20 (d, *J* = 17.7 Hz, 6H), 3.12 – 3.06 (m, 2H), 3.01 (d, *J* = 7.9 Hz, 2H), 2.51 (s, 10H), 2.34 – 2.14 (m, 5H), 2.14 – 1.99 (m, 5H), 1.98 – 1.82 (m, 4H), 1.48-1.39 (m, 3H), 1.38 – 1.26 (m, 4H), 1.02 (m, 13H), 0.97 – 0.91 (m, 15H), 0.88 (dd, *J* = 6.7, 3.5 Hz, 6H), 0.82 (dd, *J* = 15.6, 6.7 Hz, 6H). ^{13}C NMR (150 MHz, CD_3OD) δ 175.0, 174.8, 174.7, 174.6, 172.0, 171.2, 170.5, 170.4, 170.1, 156.8, 156.3, 143.5, 143.45, 135.85, 135.5, 75.1, 60.8, 59.7, 59.6, 58.5, 58.3, 57.6, 57.5, 57.3, 57.2, 56.2, 51.8, 50.7, 46.0, 44.6, 42.4, 40.35, 40.3, 32.5, 32.4, 31.7, 31.35, 30.05, 30.0, 29.4, 29.2, 28.7, 28.5, 28.4, 26.3, 26.25, 25.9, 25.8, 21.1, 20.0, 19.8, 19.5, 19.4, 19.2, 19.15, 19.1, 18.5, 14.1. ESI-MS m/z: 761.4785 $[M+H]^+$, calcd for $C_{38}H_{65}N_8O_6S^+$: 761.4670.

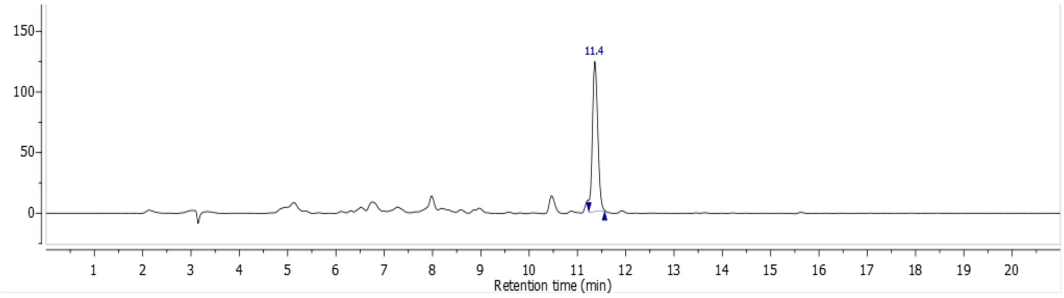

Figure S34. RP-HPLC chromatogram of pure ~~peptide~~ dolastatin analog **5h**.

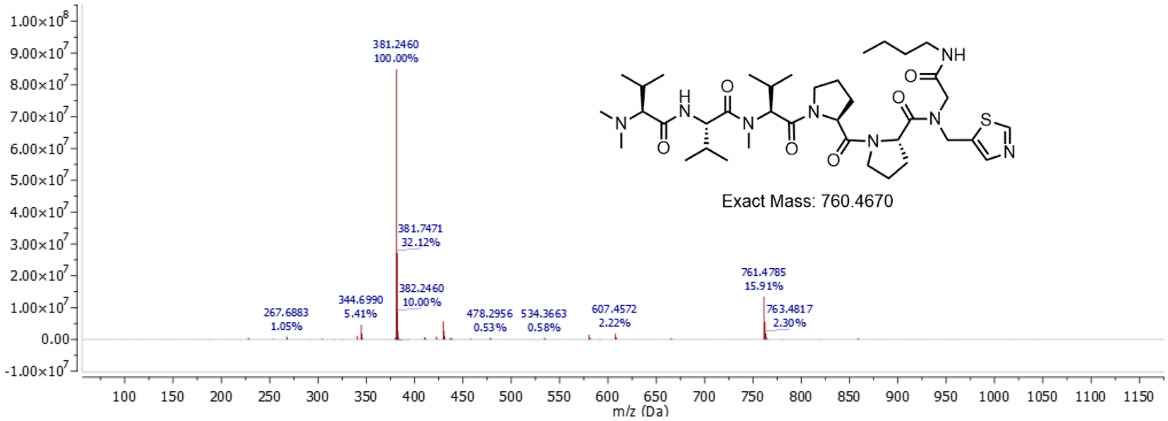

Figure S35. ESI-HRMS dolastatin analog **5h.**

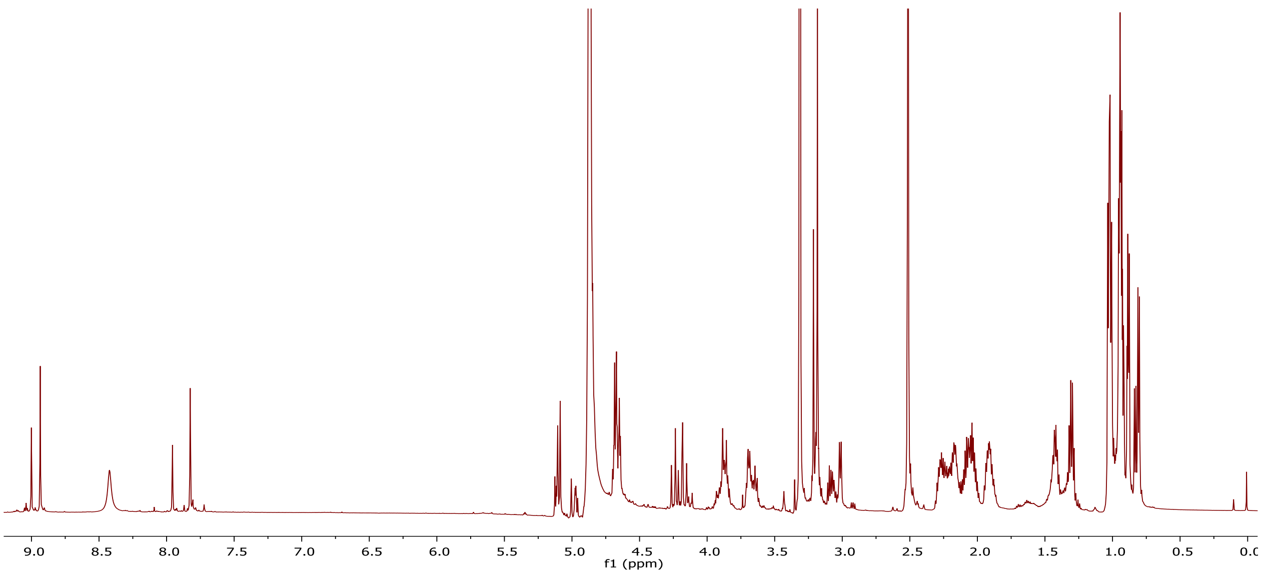

Figure S36. ^{1}H NMR spectrum in CD_3OD of dolastatin analog **5h**.

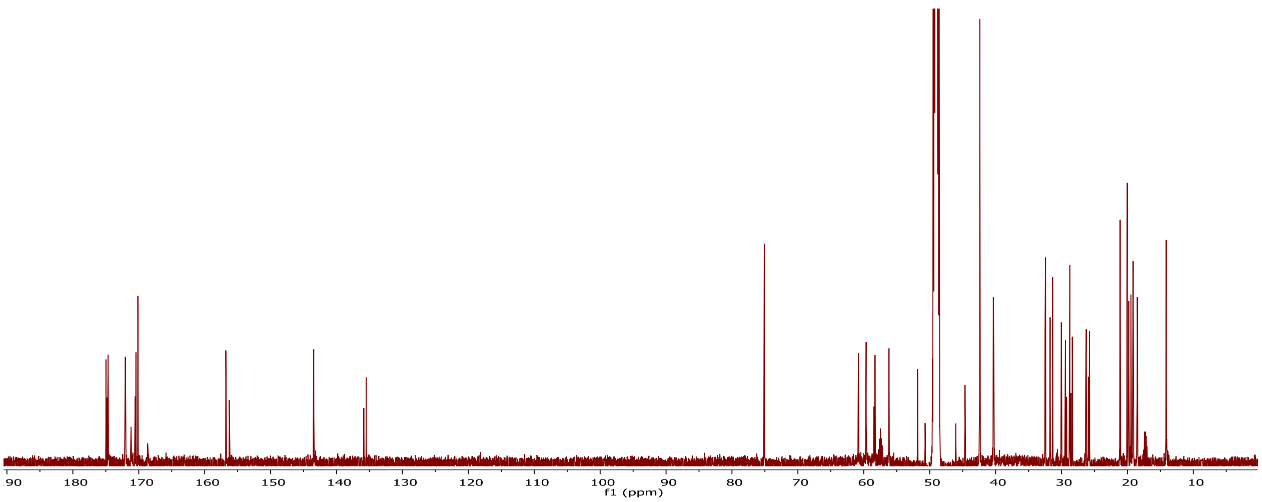

Figure S37. ^{13}C NMR spectrum in CD_3OD of dolastatin analog **5h**.

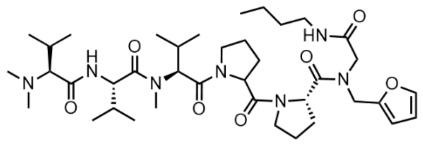

Furan-2-ylmethanamine (18 mg, 0.18 mmol), paraformaldehyde (5.4 mg, 0.18 mmol), peptide **1** (50 mg, 0.09 mmol) and *n*-butyl isocyanide (11.7 µL, 9.22 mg, 0.11 mmol) were reacted according to the general procedure described above. HPLC purification rendered the pure dolastatin analog **5i** as an amorphous white solid (35 mg, 52% isolated yield, 95% purity). R_t = 9.9 min. ^{1}H NMR (600 MHz, DMSO-d_6): δ 8.93 (br.s, 1H), 7.97; 7.39 (t, J = 5.6 Hz, 1H), 7.60; 7.56 (d, J = 1.0 Hz, 1H), 6.50; 6.25 (d, J = 3.2 Hz, 1H), 6.40; 6.38 (dd, J = 3.3, 1.9 Hz, 1H), 4.98 (dd, J = 11.0, 3.5 Hz, 1H), 4.90 (dd, J = 8.1, 5.7 Hz, 1H), 4.70; 4.51 (d, J = 16.6 Hz, 1H), 4.64 – 4.53; 4.38 (m, 3H), 3.94 (d, J = 16.5 Hz, 1H), 3.78 – 3.70 (m, 3H), 3.65 (d, J = 16.5 Hz, 1H), 3.56 – 3.45 (m, 2H), 3.14 – 3.02 (m, 4H), 3.02 – 2.86 (m, 1H), 2.80 – 2.71 (m, 6H), 2.33 – 2.22 (m, 1H), 2.13 (m, 3H), 2.05–1.94 (m, 2H), 1.93–1.83 (m, 2H), 1.82–1.70 (m, 3H), 1.35 (h, J = 6.7 Hz, 2H), 1.31 – 1.18 (m, 2H), 0.99 – 0.92 (m, 6H), 0.92 – 0.80 (m, 12H), 0.71 (dd, J = 6.7, 3.2 Hz, 3H). ^{13}C NMR (126 MHz, DMSO) δ 172.0, 171.7, 171.5, 171.3, 169.6, 169.5, 167.2, 150.3, 150.2, 142.9, 142.6, 110.5, 110.4, 108.8, 108.4, 72.1, 58.4, 57.6, 56.4, 54.5, 48.9, 47.1, 46.8, 44.8, 42.6, 38.3, 31.1, 31.0, 30.3, 29.9, 28.3, 27.6, 26.6, 24.8, 24.6, 24.3, 24.2, 19.5, 18.8, 18.5, 16.4, 13.7. ESI-MS m/z: 744.5041 [M+H]$^+$, calcd for $C_{39}H_{66}N_7O_7$: 744.4945.

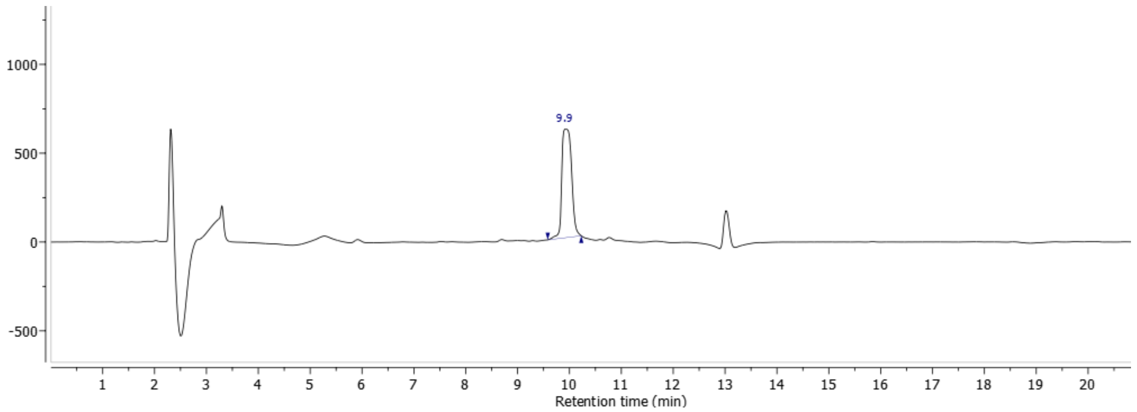

Figure S38. RP-HPLC chromatogram of pure dolastatin analog **5i**.

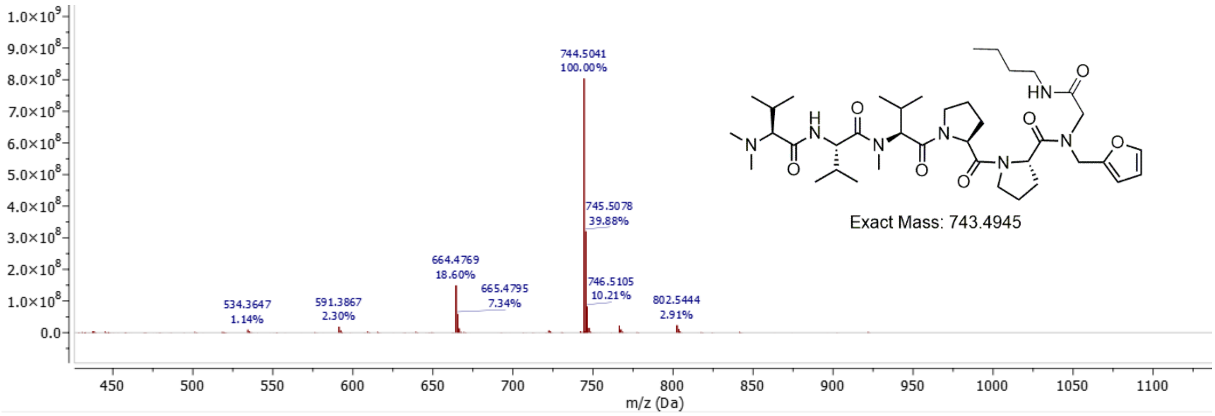

Figure S39. ESI-HRMS dolastatin analog **5i.**

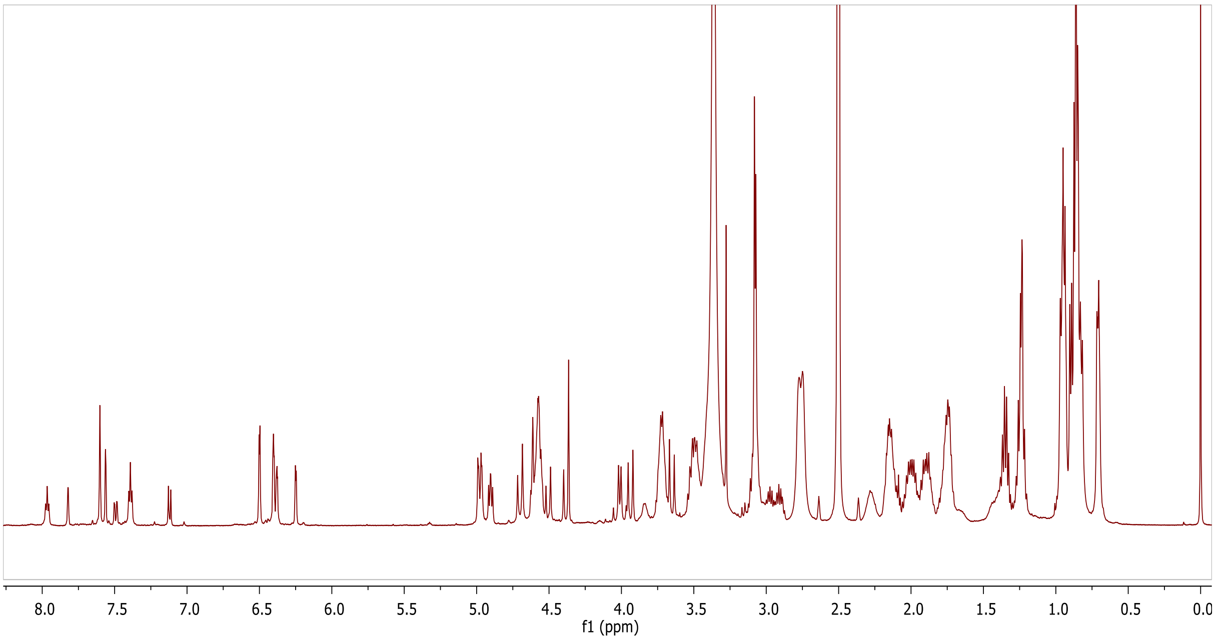

Figure S40. ^{1}H NMR spectrum in DMSO-d_6 of dolastatin analog **5i**.

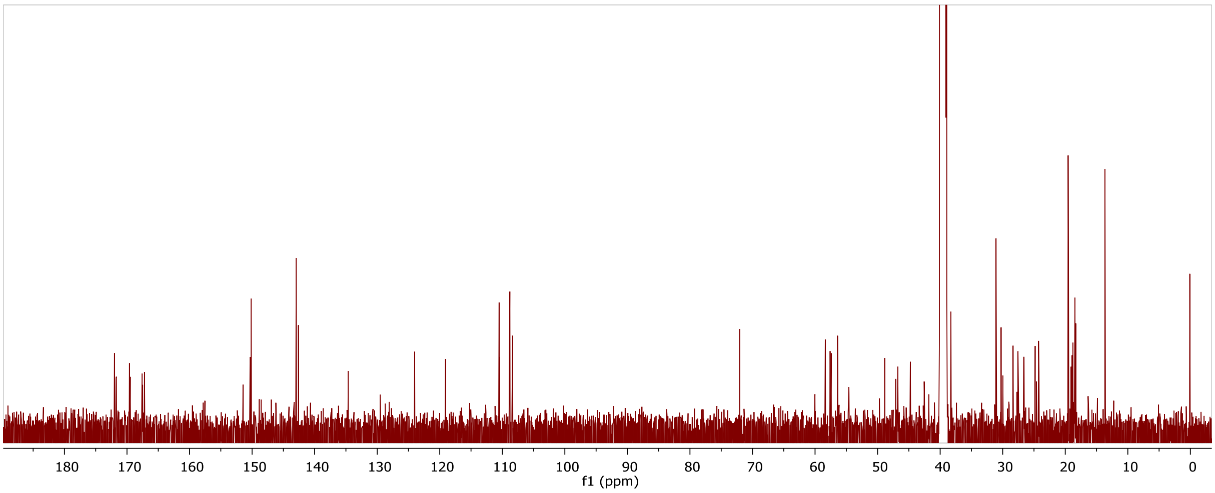

Figure S41. ^{13}C NMR spectrum in DMSO-d_6 of dolastatin analog **5i.**

Synthesis of *N*-methylcemadotin

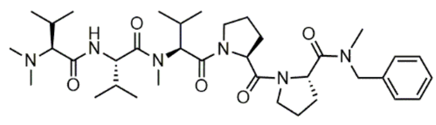

N-methylcemadotin (**5j**) was prepared by coupling *N*-methylbenzylamine (6.4 µL, 0.05 mmol) to the crude peptide **1** (13.4 mg, 0.024 mmol) using HATU (19 mg, 0.05 mmol) and NMM (11.1 µL, 0.05 mmol) in DMF overnight. *N*-methylcemadotin (**5j**) (10.1 mg, 62% isolated yield, 95% purity) was obtained as the formate salt by RP-HPLC purification. R_t = 16.1 min. ^{1}H NMR (600 MHz, DMSO-d_6) δ 8.14 (s, 1H), 7.43 (d, *J* = 7.0 Hz, 1H), 7.31 (q, *J* = 6.6, 5.5 Hz, 2H), 7.25 (q, *J* = 7.5 Hz, 1H), 7.19 (d, *J* = 6.9 Hz, 1H), 4.98 (dd, *J* = 11.0, 1.8 Hz, 1H), 4.81 (ddd, *J* = 20.0, 8.5, 4.3 Hz, 2H), 4.64 – 4.47 (m, 3H), 4.37 (dd, *J* = 26.3, 15.9 Hz, 1H), 3.78 – 3.68 (m, 2H), 3.52 (dt, *J* = 13.8, 6.7 Hz, 2H), 3.07 (d, *J* = 3.3 Hz, 3H), 2.95 (s, 2H), 2.70 (s, 1H), 2.32 – 2.05 (m, 8H), 2.00 – 1.91 (m, 4H), 1.86 (ddt, *J* = 25.0, 12.3, 6.1 Hz, 2H), 1.72 (dtd, *J* = 17.9, 10.5, 9.0, 5.0 Hz, 2H), 0.96 – 0.90 (m, 4H), 0.89 (t, *J* = 7.1 Hz, 6H), 0.83 (dd, *J* = 6.8, 2.1 Hz, 4H), 0.71 (dd, *J* = 15.5, 6.6 Hz, 6H). ^{13}C NMR (126 MHz, DMSO) δ 171.9, 169.6, 169.5, 168.2, 163.5, 138.1, 137.7, 128.9, 128.9, 127.8, 127.7, 127.4, 58.8, 58.1, 58.0, 57.1, 56.8, 52.4, 50.7, 47.6, 46.9, 41.8, 34.9, 33.5, 30.7, 30.3, 29.1, 28.5, 28.2, 28.1, 27.3, 27.1, 24.9, 24.7, 20.0, 19.5, 19.4, 19.3, 19.1, 18.7.

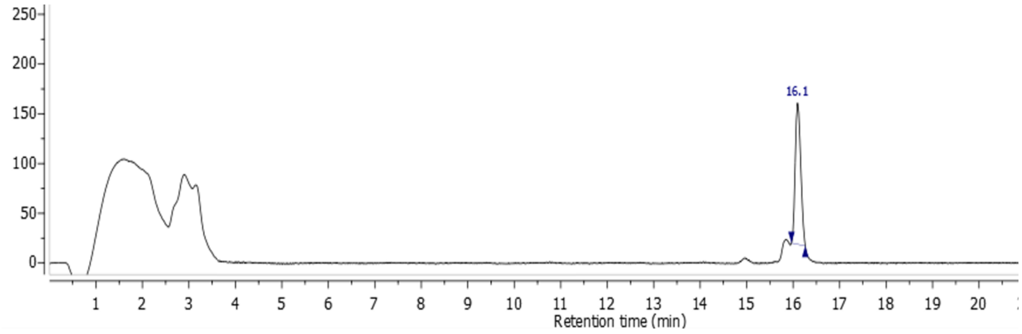

Figure S42. RP-HPLC traces the pure *N*-methylcemadotin (**5j**).

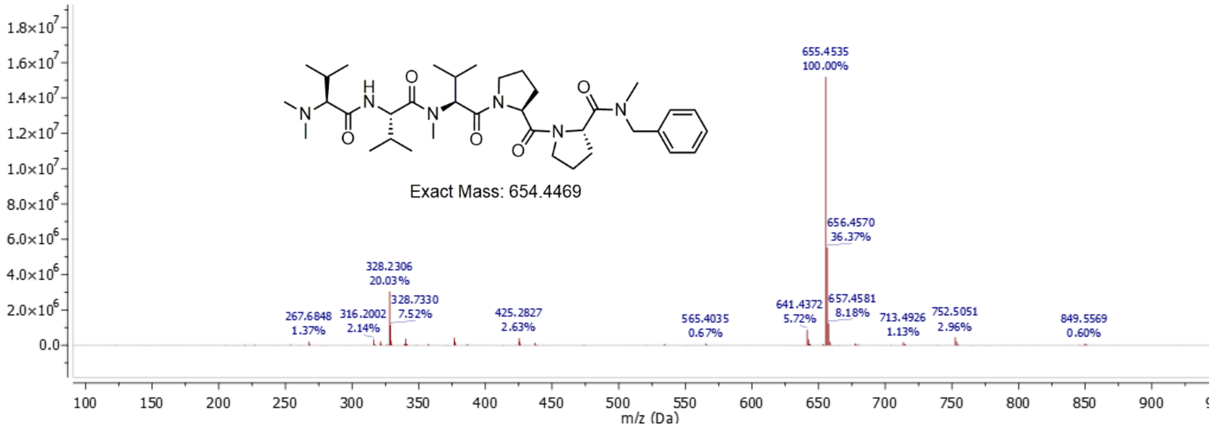

Figure S43. ESI-HRMS of *N*-methylcemadotin (**5j**).

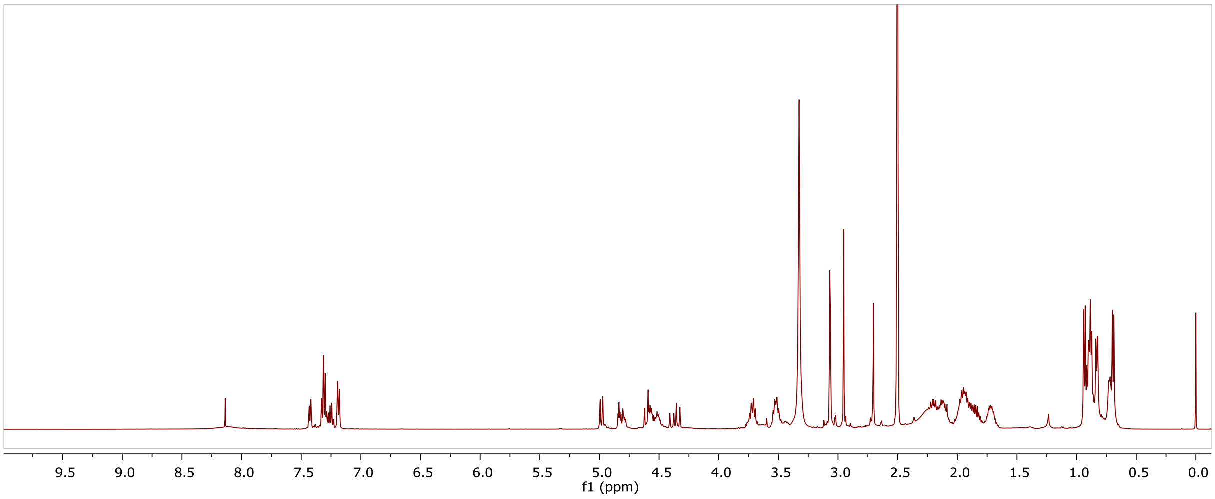

Figure S44. ^{1}H NMR spectrum of N-methylcemadotin (**5j**) in DMSO-*d*6.

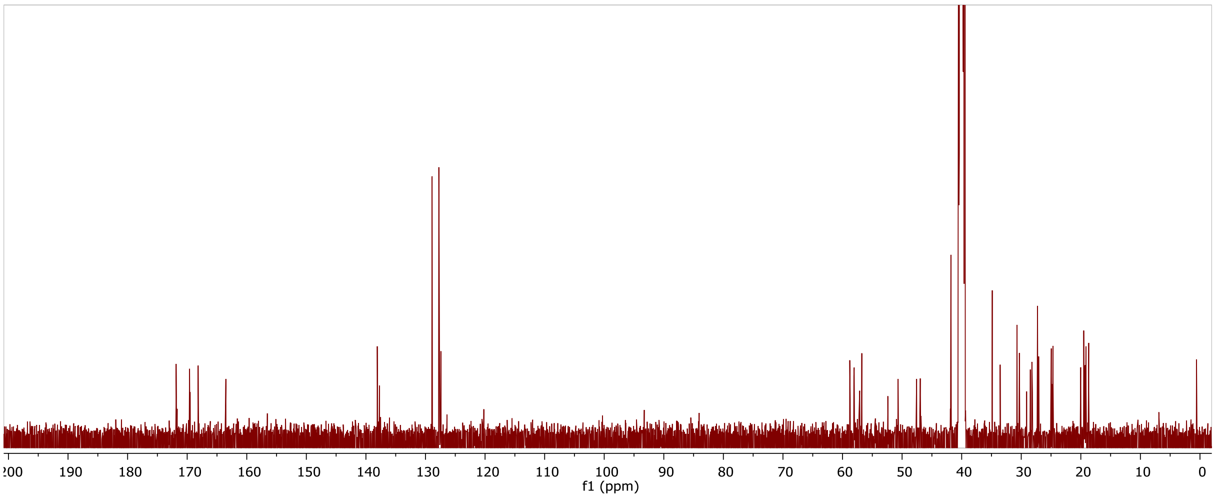

Figure S45. ^{13}C NMR spectrum of *N*-methylcemadotin (**5j**) in DMSO-*d*6.

Synthesis of isocyanide modified resin (6)

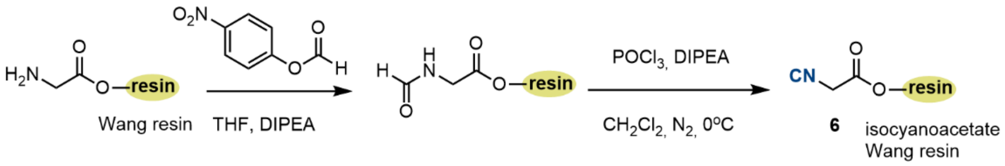

The free amino glycine-bound Wang resin (0.69 mmol) was placed in a 10 mL solid-phase reaction vessel and pre-swelled with dry DCM (2×8 mL). DIPEA (0.59 mL, 3.5 mmol, 10 equiv) and 4-nitrophenylformate (288 mg, 1.7 mmol, 5 equiv) were mixed in dry THF and reacted for 4 min. This mixture was transferred into the resin vessel and the mixture was stirred for 2 h. The reaction completion was checked by the Kaiser test, and the procedure was repeated once if it was not completed. The resin was washed with THF (3×8 mL), DMF (3×8 mL), and

DCM (2×8 mL). Dry DCM (8 mL) and DIPEA (0.9 mL, 5 mmol, 15 equiv) were added to the resin and the vessel was cold to 0º C in an external ice bath and connected to a gentle stream of N_2 (g). $POCl_3$ (0.16 mL, 1.7 mmol, 5 equiv) dissolved in 2 mL of dry DCM was added dropwise to the reaction mixture while keeping the stream of N_2 (g) and the reaction mixture was stirred for 4 hours at 0 ºC. The resin was then sequentially washed with dry DCM (3×8 mL) and DMF (3×8 mL), dried in a desiccator for 1 day and stored at -20 ºC. The isocyanide formation was checked by infrared spectroscopy.

+

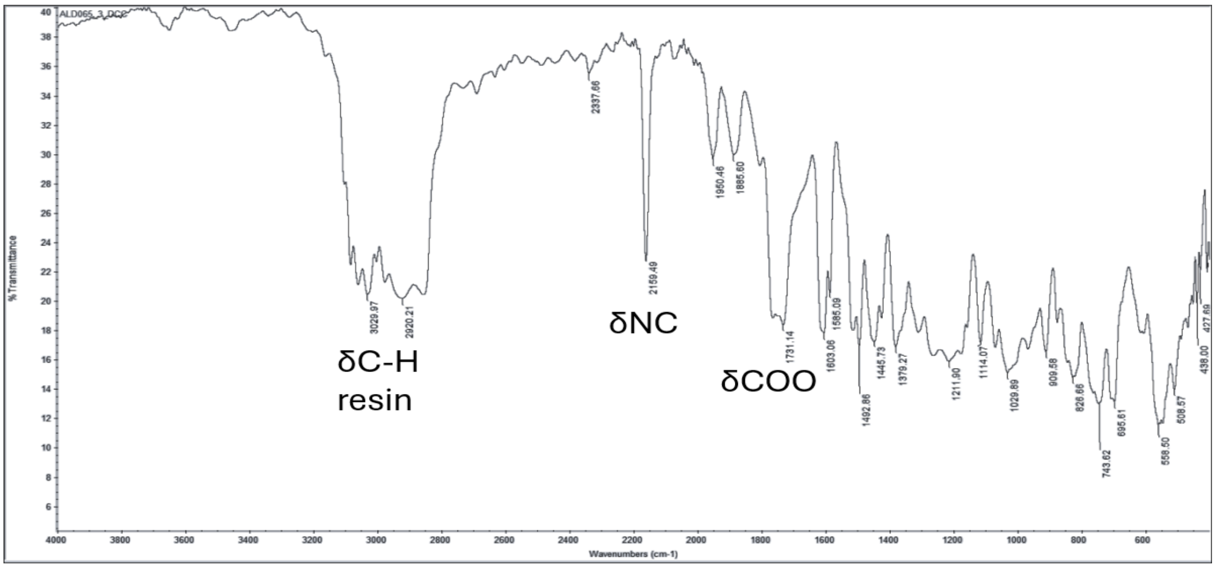

Figure S46. Infrared spectrum of the on-resin isocyanide.

All-on-resin synthesis of internal backbone amide *N*-substituted dolastatin 15 analogs

General protocol for the on-resin synthesis of the peptides 7a-g

In a 6 mL vial, the amine component (4 equiv) is mixed with 6-nitroverataldehyde (4 equiv) in a mixture of MeOH/DCM 1:1 (*v/v*) (1 mL). The reaction mixture is stirred for 2 h until formation of the imine, confirmed by TLC and ESI-MS. Fmoc-AA-OH (4 equiv) is added to the reaction mixture in 0.5 mL of DCM and the resulting mixture is transferred to a vessel containing the isocyano-resin (0.1 mmol), which is left shaking at room temperature for 48 h. The excess of reagents is removed by washing the beads sequentially with DMF (3×1 min), DCM (3×1 min) and MeOH (2×1 min). The formation of the Ugi product is checked by ESI-MS and RP-HPLC monitoring after mini-cleavage. Finally, the resin loading is determined

spectrophotometrically by UV absorption of the Fmoc group at 301 nm as described in the following.

Loading measurement: The loaded resin (5-10 mg) in an Eppendorf tube is mixed with 20% piperidine/DMF (1 mL) and stirred for 20 min. The resulting suspension is centrifuged and 100 µL of the above solution are transferred to a vessel containing DMF (10 mL). Finally, part of the above solution is transferred into a 1 mL cuvette and the absorbance is measured at 301 nm. The same procedure is repeated for the reference (same composition solution but without the resin). The loading of the resin is calculated by L(mg mmol^{-1}) = [101× (Absorbance)]/[7.8 × (weight in mg)].

General protocol for the on-resin synthesis of the peptides 8a-i

On-resin Fmoc deprotection is accomplished using the standard protocol and the resin is then washed with DMF (3×1 min), MeOH (3×1 min) and DCM (3×1 min). The free amino-containing peptide-bound resin (≈ 0.05 mmol) is subjected to imine formation by treating the resin beads with a suspension of paraformaldehyde (7.5 mg, 0.25 mmol, 5 equiv) and pyrrolidine (21 µL, 0.25 mmol, 5 equiv) in THF/MeOH (1:1) for 30 min. Excess of reagents is removed by washing the beads with THF (4×1min). The resin is transferred to a 5 mL MW tube and a solution of the Fmoc-Pro-OH (67 mg, 0.2 mmol, 4 equiv) in 2 mL of THF/MeOH (1:1) and the isocyanide (0.2 mmol, 4 equiv) are added and the mixture is stirred for 45 min in MW at 75 ºC. Completion is indicated either by negative Kaiser test, ESI-MS or by RP-HPLC monitoring after mini-cleavages. Then, the resin is transferred back to a 5 mL fritted syringe and washed with DCM (3×1 min) and DMF (2×1 min).

General protocol for the synthesis of the peptides 11a-i

Starting from the resin-linked peptide fragments **8a-i**, Fmoc-NMe-Val-OH, Fmoc-Val-OH and Fmoc-Val-OH are sequentially coupled, and the *N*-terminus is dimethylated using a reductive amination according to the procedure described above to yield resin-bound peptides **9a-i**. Finally, standard TFA cleavage conditions are used to release the peptides (**10a-i**) from the solid support.

Photo-cleavage reaction in solution: The C-terminal photocaged peptide (**10a-i**) were dissolved in 750 uL of PBS buffer (1 ×, pH 7.4) and filtered. The solution was irradiated using UV light in a closed 5 mL glass vial at 1.13 W/slot for 10 min. The final dolastatin analogs (**11a-i)** are purified using RP-HPLC.

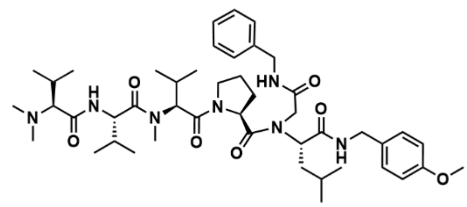

Compound **11a** was obtained as an amorphous yellow solid (10.4 mg, 25% isolated yield, 95% purity) according to the general procedure described above. The compound was purified by preparative RP-HPLC. *R*t: 20.8 min. ^{1}H NMR (600 MHz, CD_3OD) mixture of rotamers, δ 7.33 – 7.22 (m, 5H), 7.18 (d, *J* = 8.6 Hz, 2H), 6.85 – 6.81 (m, 2H), 5.04 – 4.98; 4.96 – 4.91 (m, 1H), 4.65 (t, *J* = 7.5 Hz, 1H), 4.59 – 4.51 (m, 1H), 4.43 – 4.19 (m, 12H), 3.95 – 3.86 (m, 1H), 3.76 (s, 7H), 3.69 – 3.61 (m, 2H), 3.46 – 3.42 (m, 1H), 2.99 (s, 3H), 2.89 (s, 3H), 2.86 (s, 3H), 2.43 – 2.32 (m, 1H), 2.15 (s, 2H), 2.03 (s, 2H), 1.95 – 1.80 (m, 2H), 1.79 – 1.70 (m, 1H), 1.68 – 1.48 (m, 2H), 1.36 – 1.24 (m, 1H), 0.99 – 0.80 (m, 28H). ^{13}C NMR (151 MHz, CD_3OD) δ 174.5, 173.2, 171.4, 170.3, 168.8, 163.5, 163.5, 160.5, 159.0, 158.9, 131.4, 139.6, 136.4, 131.5, 130.2, 129.5, 128.9, 128.8, 128.7, 128.6, 128.4, 115.1, 115.0, 114.9, 114.8, 74.9, 60.65, 58.8, 57.6, 57.2, 56.2, 55.7, 53.2, 49.7, 49.3, 44.4, 43.8, 42.4, 42.2, 39.2, 38.3, 31.7, 31.3, 30.7, 28.7, 28.6, 28.5, 26.4, 26.25, 25.7, 23.7, 23.4, 22.3, 22.0, 20.0, 19.4, 19.15, 19.10, 19.0, 17.5, 17.4, 17.3, 17.15. HR-MS m/z: 834.5500 $[M+H]^+$, calcd. for $C_{46}H_{72}N_7O_7^+$: 834.5415.

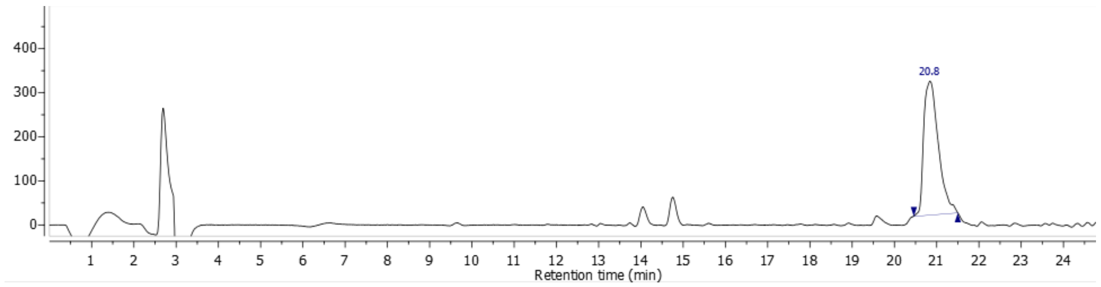

Figure S47. RP-HPLC traces of pure dolastatin analog **11a**.

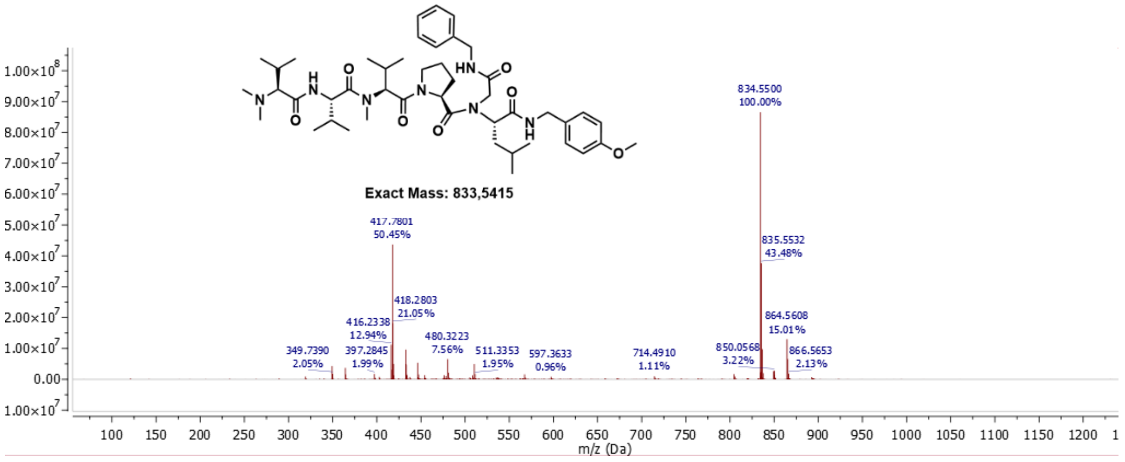

Figure S48. ESI-HRMS of dolastatin analog **11a**.

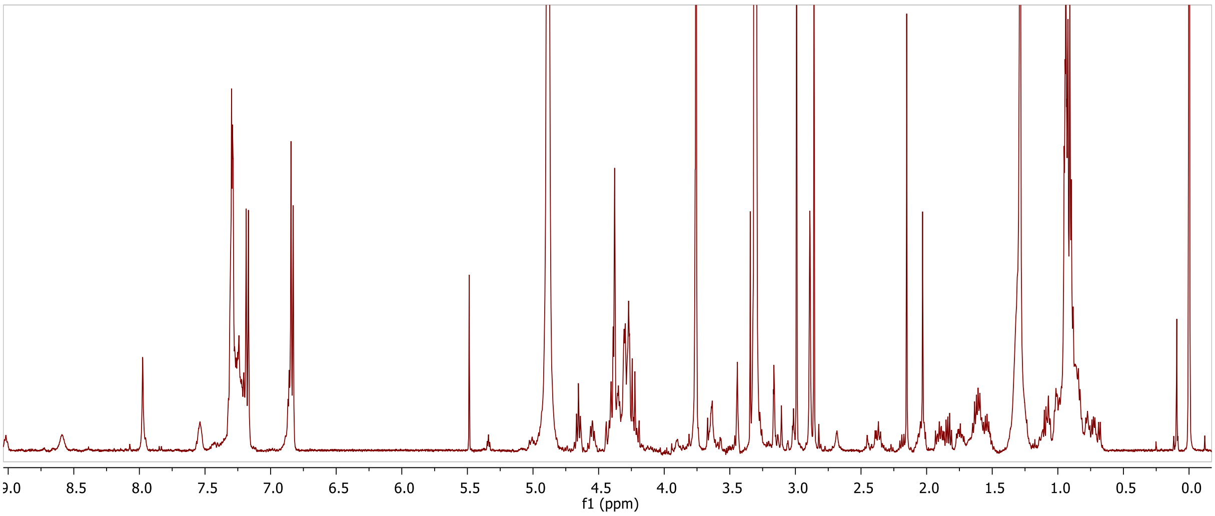

Figure S49. ^{1}H NMR spectrum in CD_3OD of dolastatin analog **11a**.

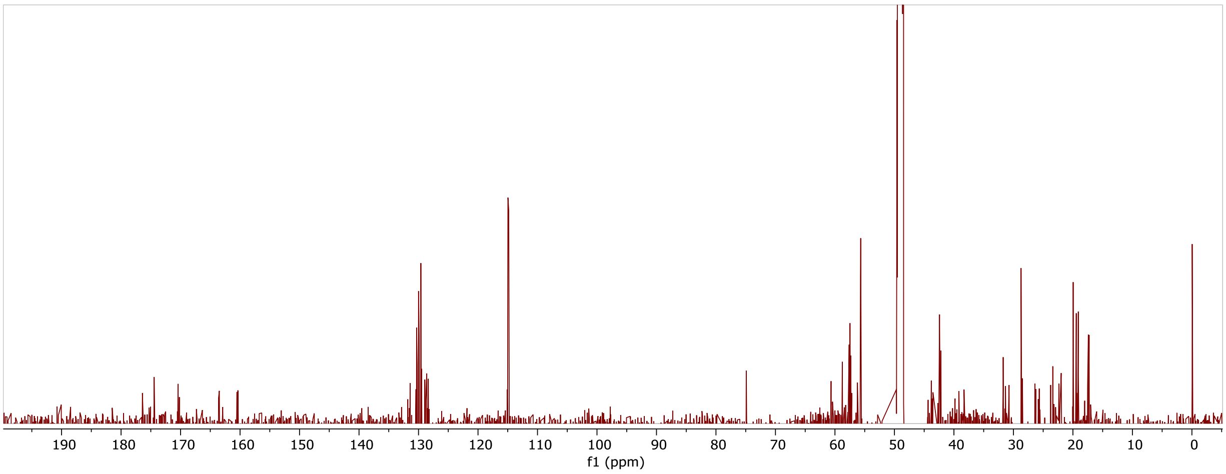

Figure S50. ^{13}C NMR spectrum in CD_3OD of dolastatin analog **11a**.

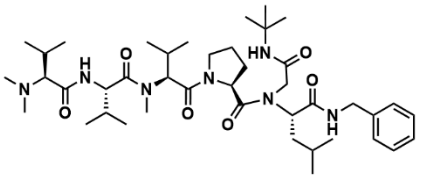

Compound **11b** was obtained as an amorphous white solid (11.5 mg, 30% isolated yield, >95% purity) according to the general procedure described above. The compound was purified by preparative RP-HPLC. R_t: 20.9 min. ^{1}H NMR (600 MHz, CD_3OD) mixture of rotamers, δ 8.52 (s, 1H), 7.38 – 7.19 (m, 5H), 5.03; 4.94 (dd, *J* = 11.0, 4.9 Hz, 2H), 4.81 (d, *J* = 15.2 Hz, 1H), 4.64 (d, *J* = 8.3 Hz, 2H), 4.61 – 4.57 (m, 1H), 4.46 (d, *J* = 18.0 Hz, 1H), 4.42 (d, *J* = 16.0 Hz, 1H), 4.37 (d, *J* = 14.9 Hz, 1H), 4.31 (d, *J* = 15.0 Hz, 1H), 4.13 (d, *J* = 15.5 Hz, 1H), 4.03 (d, *J* = 18.0 Hz, 1H), 3.94 – 3.85 (m, 2H), 3.74 – 3.64 (m, 2H), 3.61 – 3.53 (m, 1H), 3.17; 3.03 (s, 3H), 2.68 (d, *J* = 9.0 Hz, 1H), 2.31 (s, 6H), 2.24–2.17 (m, 1H), 2.16 – 2.10 (m, 2H), 2.07 – 1.99 (m, 2H), 1.92 – 1.84 (m, 2H), 1.83 – 1.75 (m, 2H), 1.74 – 1.64 (m, 2H), 1.63 – 1.53 (m, 1H), 1.39 – 1.33 (m, 1H), 1.33; 1.31 (s, 9H), 1.03 – 0.94 (m, 9H), 0.93 – 0.86 (m, 9H), 0.83 (d, *J* = 6.6 Hz, 3H), 0.82 – 0.74(m); 0.71 (d, *J* = 6.5 Hz); 0.67 (d, *J* = 6.7 Hz), 3H)). ^{13}C NMR (151 MHz, CD_3OD) δ 178.0, 176.8, 175.1, 174.9, 174.8, 173.6, 173.2, 171.9, 170.7, 170.2, 169.5, 139.8, 137.6, 129.8, 129.55, 129.45, 129.0, 128.9, 128.8, 128.7, 128.5, 128.1, 75.6, 72.5, 60.7, 58.65, 58.05, 56.0, 52.5, 49.6, 44.9, 44.7, 44.1, 43.2, 42.4, 42.0, 39.3, 31.7, 31.7, 31.4, 31.0, 28.9, 28.9, 28.9, 28.8, 28.55, 26.2, 26.1, 25.6, 25.3, 24.1, 23.7, 23.6, 23.3, 22.7, 22.2, 21.9, 21.8, 20.1, 19.6, 19.5, 19.4, 19.2, 19.1. HR-MS m/z: 770.5580 $[M+H]^+$, calcd. for $C_{42}H_{72}N_7O_6^+$: 770.5466.

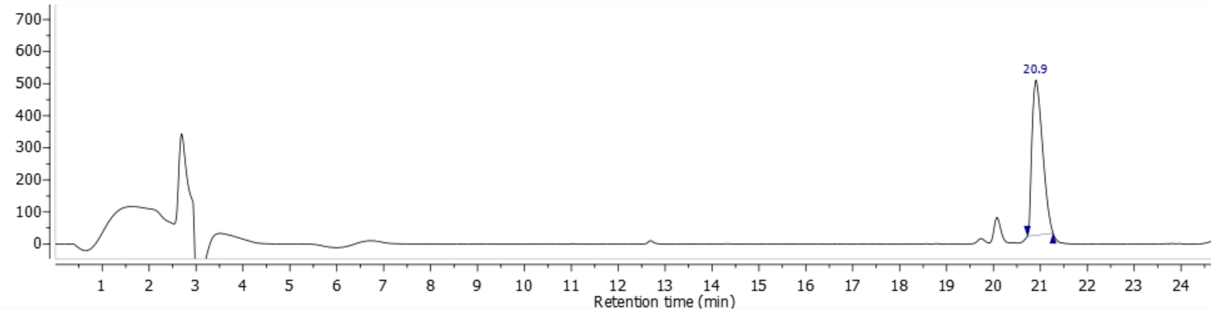

Figure S51. RP-HPLC traces of pure dolastatin analog **11b**.

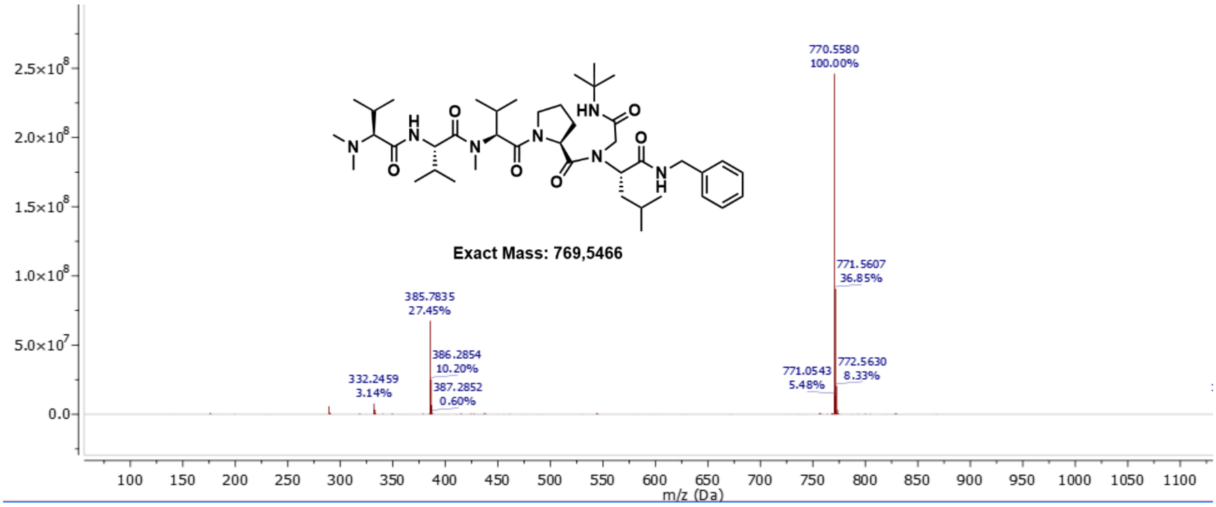

Figure S52. ESI-HRMS of dolastatin analog **11b**.

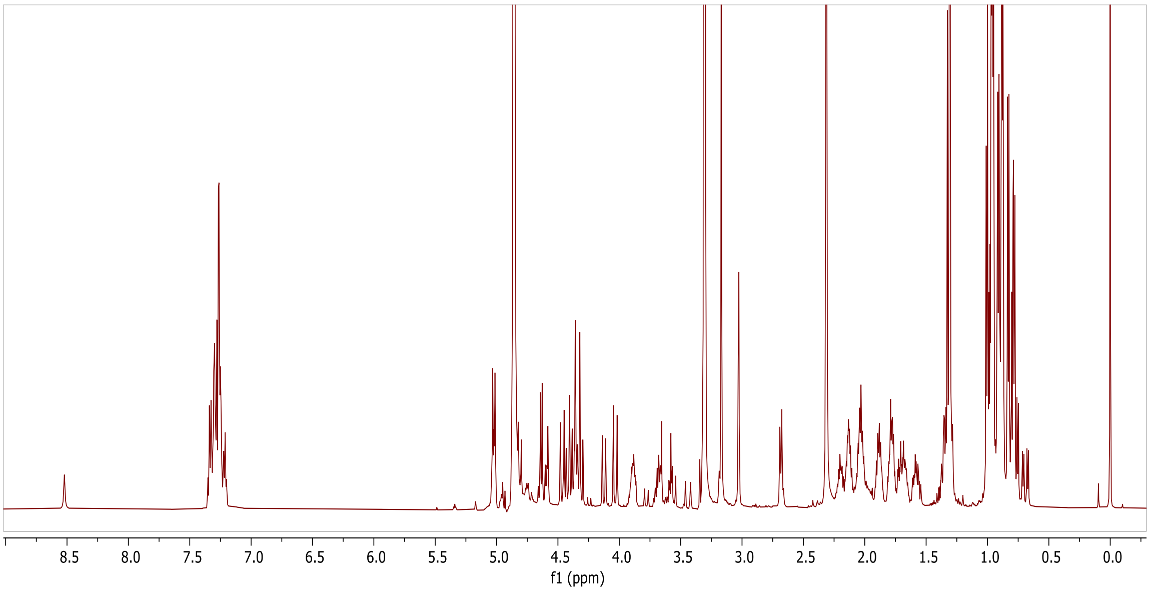

Figure S53. ^{1}H NMR spectrum in CD_3OD of dolastatin analog **11b**.

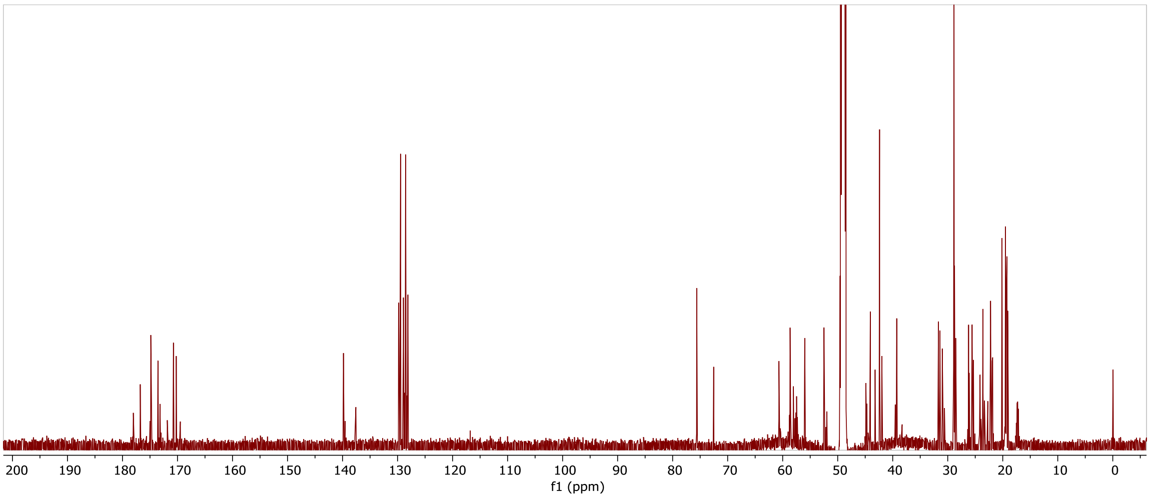

Figure S54. ^{13}C NMR spectrum in CD_3OD of dolastatin analog **11b**.

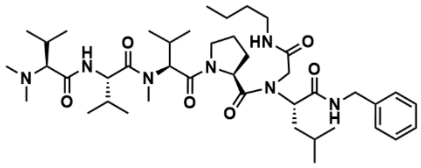

Compound **11c** was obtained as an amorphous white solid (8.8 mg, 23% isolated yield, >95% purity) according to the general procedure described above. The compound was purified by preparative RP-HPLC. R_t: 20.5 min. ^{1}H NMR (600 MHz, CD_3OD) mixture of rotamers, δ 8.24 (s, 1H), 7.41 –7.19 (m, 5H), 5.04 (dd, J = 11.1, 2.9 Hz, 1H), 5.03 – 4.99 (m, 1H), 5.01 – 4.95 (m, 1H), 4.75 (t, J = 7.1 Hz, 1H), 4.68; 4.64 (d, J = 8.5, 1H), 4.50 (d, J = 18.3 Hz, 1H), 4.44 – 4.39 (m, 2H), 4.38 (d, J = 15.0 Hz, 1H), 4.30 (d, J = 15.1 Hz, 1H), 4.15 (d, J = 18.1 Hz, 1H), 3.95 – 3.87 (m, 1H), 3.85 – 3.79 (m, 1H), 3.79 – 3.64 (m, 2H), 3.21 – 3.18 (m, 2H), 3.17(s) ; 3.05(d, J = 8.4 Hz, 3H), 2.67 (s, 12H), 2.30 – 2.18 (m, 3H), 2.17 – 2.00 (m, 3H), 1.97 – 1.83 (m, 2H), 1.82-1.73 (m, 1H), 1.72 – 1.61 (m, 2H), 1.56 – 1.44 (m, 5H), 1.41 – 1.27 (m, 8H), 1.08 – 0.98 (m, 6H), 0.97 – 0.88 (m, 18H), 0.78; 0.70 (dd, J = 12.7, 6.6 Hz, 3H). ^{13}C NMR (151 MHz, CD_3OD) δ 176.4, 174.4, 173.4, 171.6, 170.3, 139.9, 129.6, 129.5, 129.0, 128.5, 128.4, 128.1, 74.7, 60.7, 58.8, 57.6, 57.5, 57.3, 56.3, 49.6, 44.0, 42.45, 40.4, 39.2, 32.5, 32.4, 31.7, 31.4, 30.8, 28.7, 28.6, 26.2, 25.7, 23.4, 23.2, 22.9, 22.4, 21.1, 19.9, 19.5, 19.4, 19.2, 19.05, 17.5, 17.3, 14.1. HR-MS m/z: 770.5547 $[M+H]^+$, calcd. for $C_{42}H_{72}N_7O_6^+$: 770.5466.

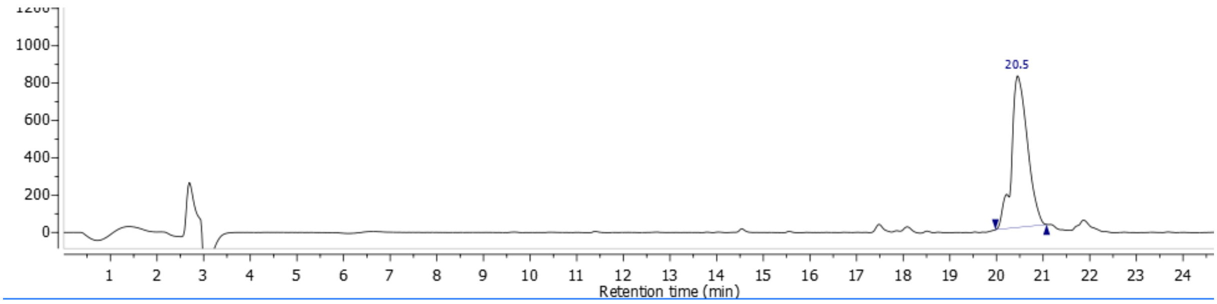

Figure S55. RP-HPLC traces of pure dolastatin analog **11c**.

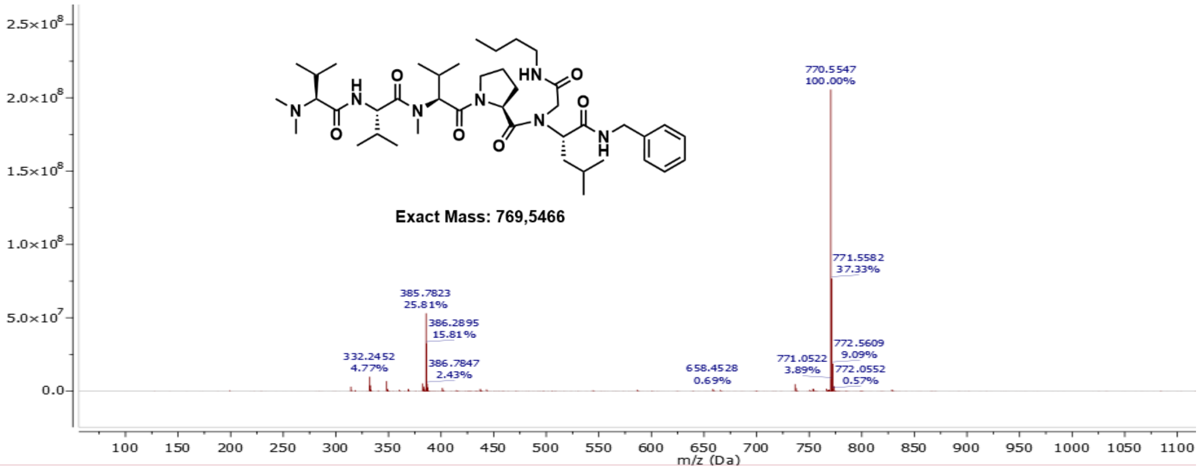

Figure S56. ESI-HRMS of dolastatin analog **11c**.

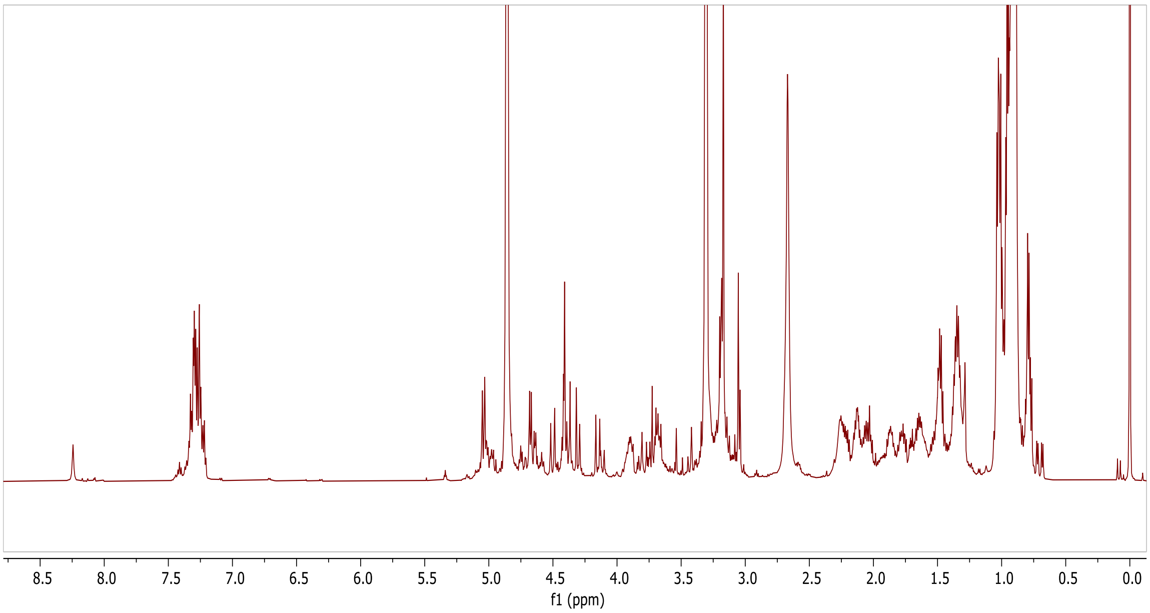

Figure S57. ^{1}H NMR spectrum in CD3OD of dolastatin analog **11c**.

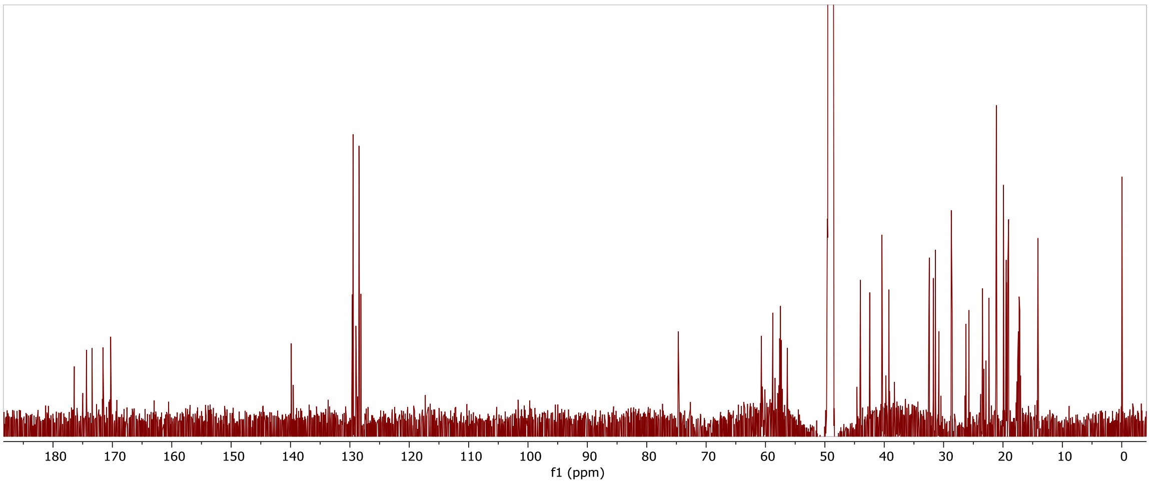

Figure S58. ^{13}C NMR spectrum in CD3OD of dolastatin analog **11c**.

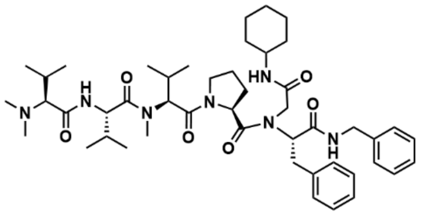

Compound **11d** was obtained as an amorphous white solid (11.6 mg, 28% isolated yield, 95% purity) according to the general procedure described above. The compound was purified by preparative RP-HPLC. R_t: 21.2 min. ^{1}H NMR (600 MHz, CD_3OD) mixture of rotamers, δ 7.34 – 7.17 (m, 10H), 5.05; 4.97 (d, *J* = 11.0 Hz, 1H), 5.02 (dd, *J* = 9.5, 4.4 Hz, 1H), 4.68 (t, *J* = 7.1 Hz, 1H), 4.63 (d, *J* = 9.0 Hz, 1H), 4.58 (t, *J* = 7.5 Hz, 2H), 4.50 (d, *J* = 4.8 Hz, 1H), 4.44 (d, *J* = 15.6 Hz, 1H), 4.41 (d, *J* = 7.1 Hz, 1H), 4.32 (d, *J* = 14.8 Hz, 1H), 4.20 (d, *J* = 15.1 Hz, 1H), 4.09 (d, *J* = 15.8 Hz, 1H), 4.01 (d, *J* = 18.2 Hz, 1H), 3.96-3.89 (m, 2H), 3.86 – 3.78 (m, 2H), 3.72-3.67 (m, 1H), 3.64 – 3.50 (m, 3H), 3.30 (brs, 1H), 3.28 – 3.18 (m, 2H), 3.17, 3.16 , 3.02 (s, 3H), 3.12 – 3.05 (m, 1H), 2.43 (s, 6H), 2.24 – 1.95 (m, 5H), 1.94 – 1.86 (m, 1H), 1.85 – 1.67 (m, 4H), 1.66-1.55 (m, 2H), 1.38 – 1.24 (m, 5H), 1.23 – 1.10 (m, 5H), 0.99 (dd, *J* = 10.0, 6.7 Hz, 6H), 0.95 (d, *J* = 6.4 Hz, 3H), 0.90 (t, *J* = 6.1 Hz, 3H), 0.87 – 0.83 (m, 6H), 0.79; 0.74 (d, *J* = 6.6 Hz, 3H). ^{13}C NMR (151 MHz, CD_3OD) δ 175.4, 175.0, 174.65, 172.25, 172.0, 170.9, 170.4, 170.2, 169.9, 169.6, 139.8, 139.3, 138.8, 130.9, 130.7, 130.3, 129.85, 129.7, 129.6, 129.4, 129.04, 128.8, 128.45, 128.4, 128.1, 128.0, 127.9, 60.6, 60.5, 59.1, 58.55, 57.6, 57.5, 57.3, 56.1, 50.2, 50.0, 49.6, 44.5, 44.2, 42.4, 36.5, 35.8, 35.3, 33.6, 33.6, 33.5, 31.7, 31.6, 31.5, 31.4, 31.4, 30.1, 29.9, 28.75, 28.7, 28.6, 28.6, 28.5, 26.7, 26.6, 26.6, 26.2, 26.1, 26.0, 20.1, 19.6, 19.55, 19.5, 19.2, 19.1, 17.5, 17.4, 17.3, 17.2. HR-MS m/z: 830.5572 $[M+H]^+$, calcd. for $C_{47}H_{72}N_7O_6^+$: 830.5566.

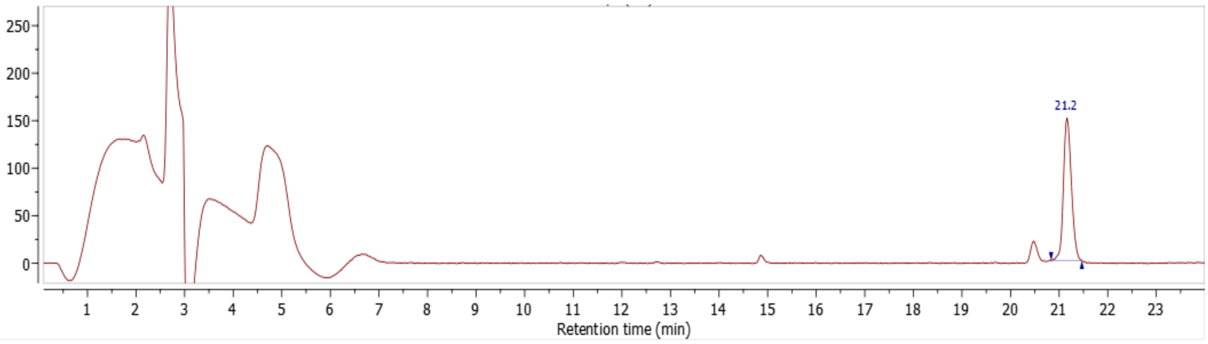

Figure S59. RP-HPLC traces of pure dolastatin analog **11d**.

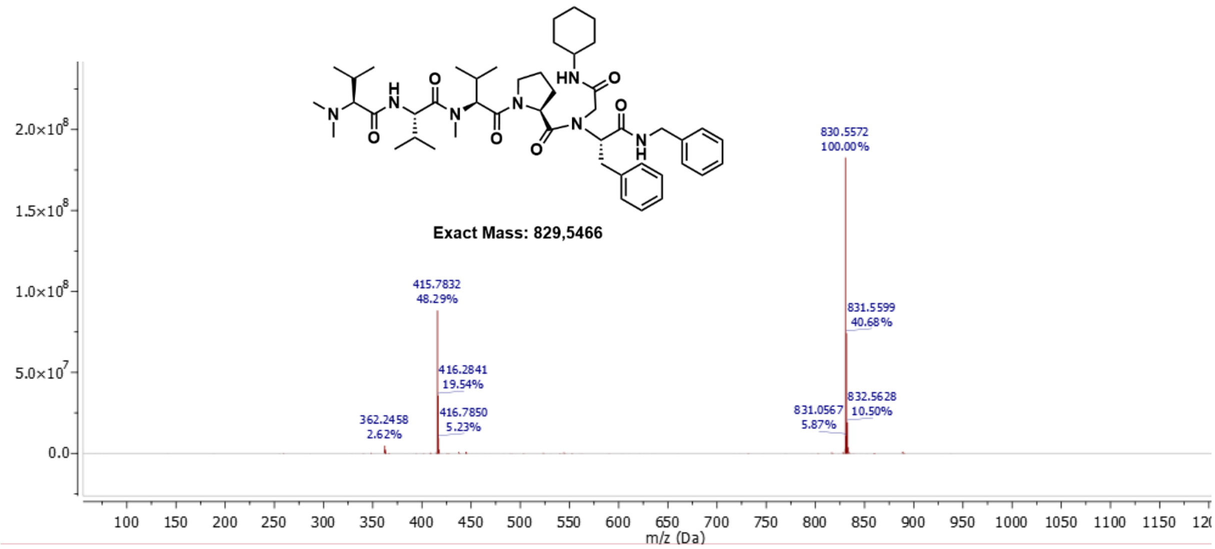

Figure S60. ESI-HRMS of dolastatin analog **11d**.

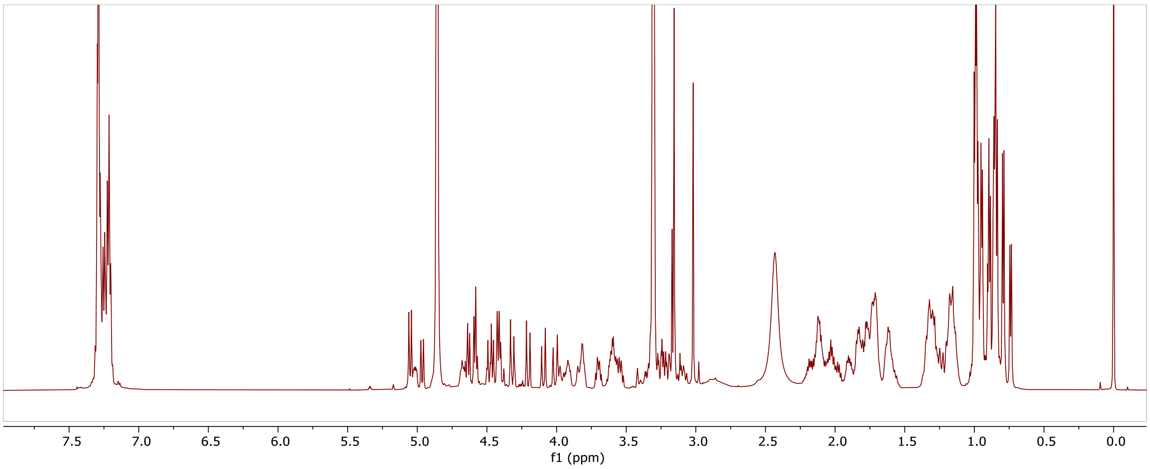

Figure S61. ^{1}H NMR spectrum in CD_3OD of dolastatin analog **11d**.

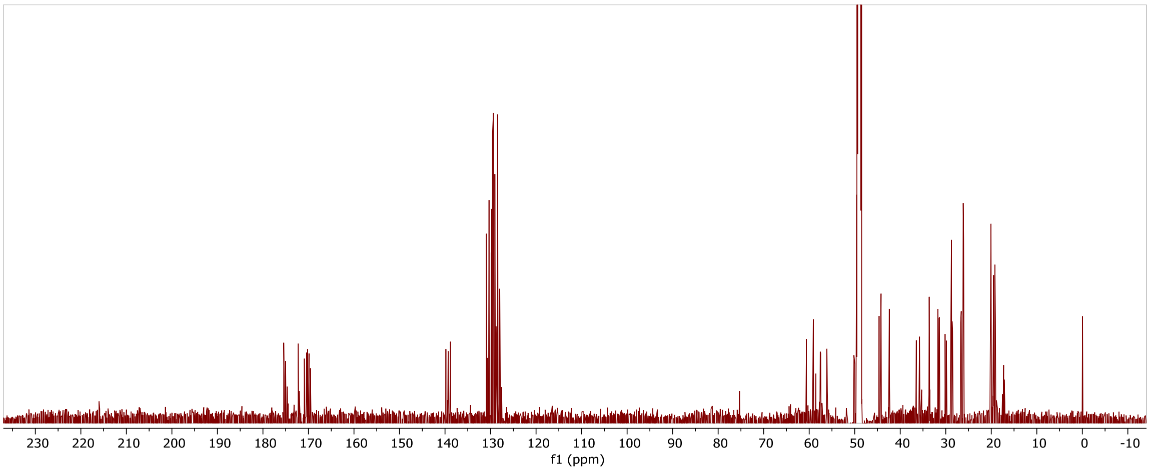

Figure S62. ^{13}C NMR spectrum in CD_3OD of dolastatin analog **11d**.

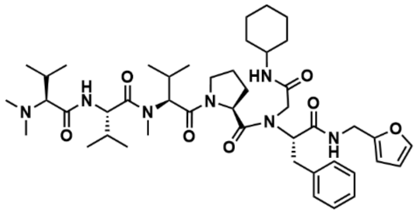

Compound **11e** was obtained as an amorphous white solid (7.8 mg, 19% isolated yield, >95% purity) according to the general procedure described above. The compound was purified by preparative RP-HPLC. R_t: 20.9 min. ^{1}H NMR (600 Hz, CD_3OD) mixture of rotamers, δ 7.42; 7.37 (s, 1H), 7.31 – 7.25 (m, 3H), 7.25 – 7.18 (m, 2H), (6.34 (dd, J = 3.2, 1.9 Hz); 6.32 – 6.27 (m); 6.20 (d, J = 3.2 Hz) 2H)), 5.06 (d, J = 11.0, 1H), 4.99 (dd, J = 11.0, 5.2 Hz, 2H), 4.69 – 4.57 (m, 2H), 4.55 (t, J = 7.4 Hz, 1H), 4.47 (d, J = 15.5 Hz, 1H), 4.42 (d, J = 15.4 Hz, 1H), 4.39 (d, J = 7.3 Hz, 1H), 4.33 (d, J = 15.5 Hz, 1H), 4.18 (d, J = 15.7 Hz, 1H), 4.08 (d, J = 15.9 Hz, 1H), 3.98 (d, J = 18.2 Hz, 1H), 3.94 – 3.89 (m, 1H), 3.86 –3.81 (m, 1H), 3.76 (d, J = 18.1 Hz, 1H), 3.73 – 3.67 (m, 1H), 3.64 – 3.52 (m, 2H), 3.28 (d, J = 6.8 Hz, 1H), 3.25 – 3.19 (m, 2H), 3.18; 3.12 (s, 3H), 3.10 – 3.06 (m, 1H), 2.52 (brs, 6H), 2.29 – 2.20 (m, 1H), 2.19 – 2.08 (m, 3H), 2.08 – 1.98 (m, 1H), 1.92 – 1.67 (m, 6H), 1.67 – 1.56 (m, 2H), 1.40 – 1.27 (m, 3H), 1.21-1.11 (m, 3H), 1.03 – 0.97 (m, 6H), 0.96 (d, J = 6.6 Hz, 3H), 0.93 – 0.84 (m, 6H), 0.81; 0.77 (d, J = 6.5 Hz, 3H). ^{13}C NMR (151 MHz, CD_3OD) δ 175.4, 175.0, 172.3, 170.9, 170.3, 170.2, 169.9, 169.6, 153.1, 152.6, 143.5, 143.15, 143.0, 138.8, 130.9, 130.7, 130.4, 130.3, 129.8, 129.7, 129.6, 128.1, 127.8, 127.55, 111.4, 111.3, 108.6, 108.2, 107.95, 60.6, 60.5, 59.1, 57.6, 57.5, 57.3, 57.2, 56.2, 50.2, 50.0, 49.6, 42.4, 37.5, 37.45, 37.3, 36.4, 35.7, 33.6, 33.55, 31.7, 31.7, 31.5, 31.45, 30.1, 29.8, 28.7, 28.6, 26.7, 26.6, 26.2, 26.1, 20.0, 19.6, 19.5, 19.4, 19.2, 19.1, 17.5, 17.4, 17.3, 17.2. HR-MS m/z: 820.5345 $[M+H]^+$, calcd. for $C_{45}H_{70}N_7O_7^+$: 820.5331.

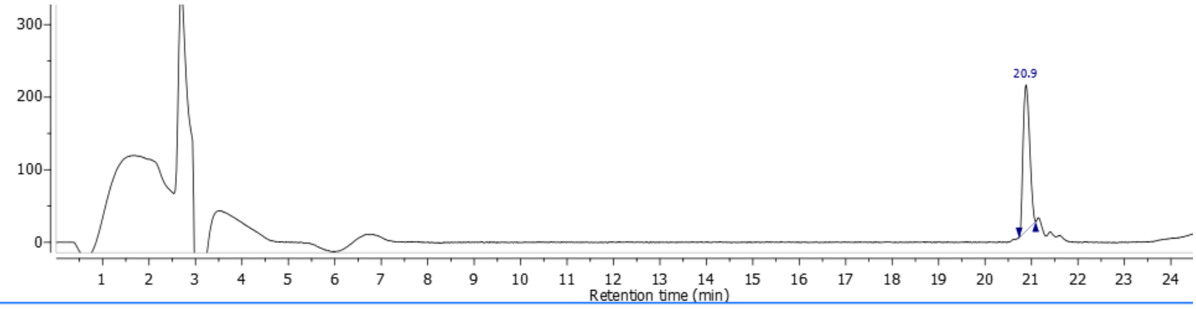

Figure S63. RP-HPLC traces of pure dolastatin analog **11e**.

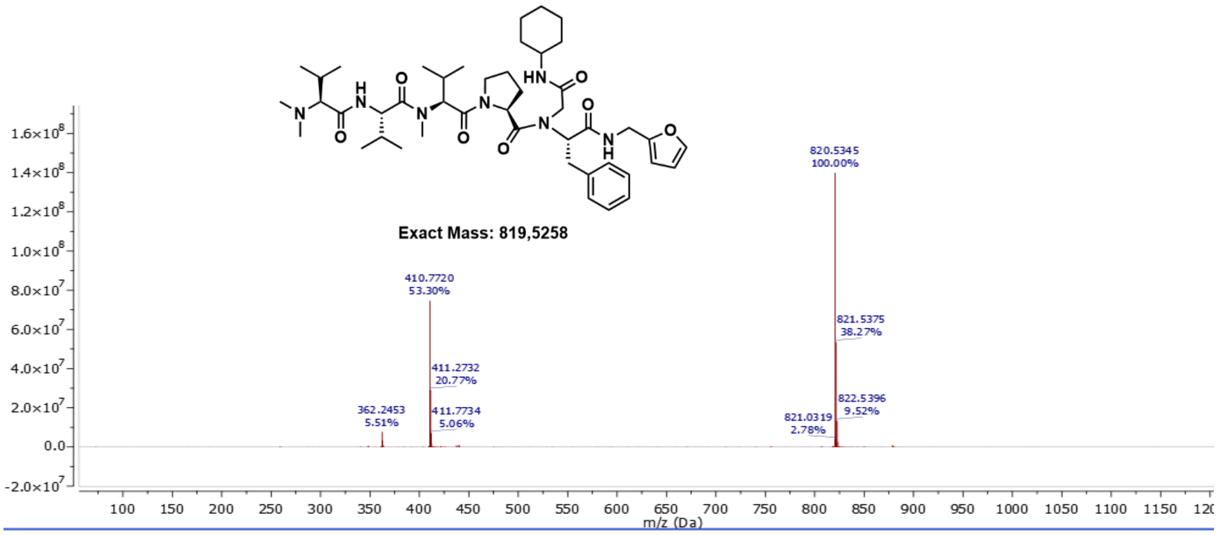

Figure S64. ESI-HRMS of dolastatin analog **11e**.

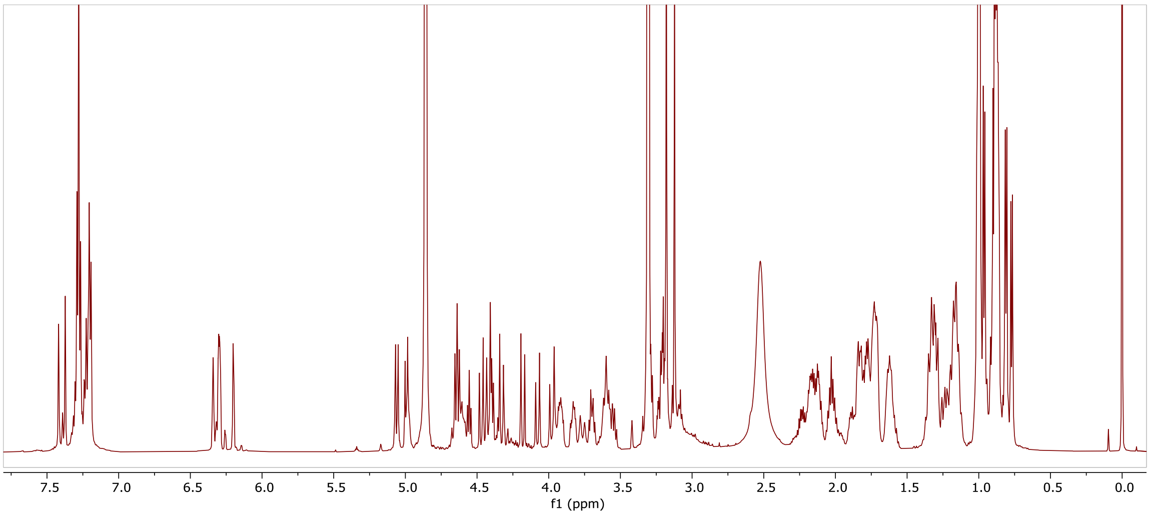

Figure S65. ^{1}H NMR spectrum in CD_3OD of dolastatin analog **11e**.

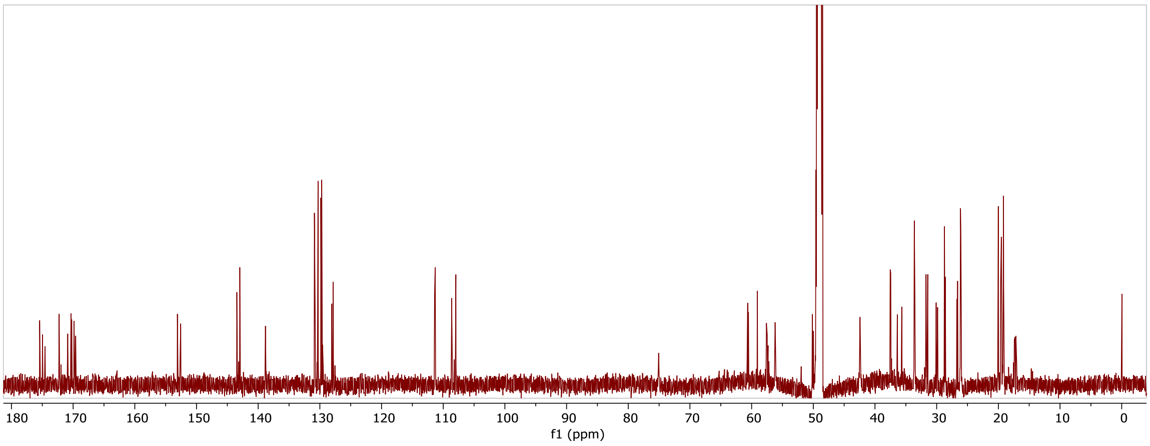

Figure S66. ^{13}C NMR spectrum in CD_3OD of dolastatin analog **11e**.

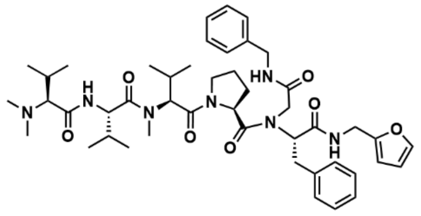

Compound **11f** was obtained as an amorphous white solid (8.6 mg, 21% isolated yield, 99% purity) according to the general procedure described above. The compound was purified by preparative RP-HPLC. R_t: 20.4 min. ^{1}H NMR (600 MHz, CD_3OD) mixture of rotamers, δ 7.42; 7.37 (d, *J* = 1.9 Hz, 1H), 7.31 – 7.17 (m, 10H), 6.34; 6.30 (dd, *J* = 3.2, 1.9 Hz, 1H), 6.24; 6.17 (d, *J* = 3.2 Hz, 1H), 5.03 (dd, *J* = 11.1, 3.7 Hz, 1H), 4.92 (d, *J* = 11.0 Hz, 1H), 4.72 – 4.68 (m, 1H), 4.62 – 4.52 (m, 2H), 4.44 – 4.36 (m, 3H), 4.32 – 4.24 (m, 1H), 4.18 (s, 2H), 4.16 (d, *J* = 15.7 Hz, 1H), 4.13 (d, *J* = 15.6 Hz, 1H), 3.96 (d, *J* = 18.3 Hz, 2H), 3.88 – 3.78 (m, 1H), 3.70 – 3.64 (m, 1H), 3.54 – 3.48 (m, 1H), 3.28 (d, *J* = 10.4 Hz, 1H), 3.20 – 3.13 (m, 1H), 3.13; 3.03 (d, *J* = 4.5 Hz, 2H), 2.35 (d, *J* = 10.0 Hz, 6H), 2.23– 2.15 (m, 1H), 2.12 – 1.93 (m, 4H), 1.93 – 1.83 (m, 1H), 1.82 – 1.71 (m, 1H), 1.64 – 1.51 (m, 1H), 1.36 – 1.27 (m, 1H), 1.13 – 1.06 (m, 1H), 1.01– 0.94 (m, 6H), 0.93 (d, *J* = 6.5 Hz, 3H), 0.90 – 0.83 (m, 6H), 0.80; 0.70 (dd, *J* = 20.9, 6.7 Hz, 3H). ^{13}C NMR (151 MHz, CD_3OD) δ 175.6, 175.1, 174.8, 174.7, 172.2, 170.9, 170.7, 170.6, 170.3, 153.0, 152.4, 143.55, 143.0, 139.8, 139.75, 138.7, 138.6, 130.9, 130.7, 130.2, 129.9, 129.7, 129.6, 129.5, 128.9, 128.8, 128.55, 128.4, 128.3, 128.2, 128.1, 127.8, 111.45, 111.3, 108.7, 108.0, 75.5, 60.6, 60.4, 59.0, 57.8, 57.6, 57.5, 57.3, 56.0, 55.9, 49.6, 44.3, 44.2, 42.4, 37.4, 36.4, 35.75, 31.7, 31.6, 31.5, 31.3, 30.1, 29.6, 28.8, 28.7, 28.4, 26.25, 26.1, 20.1, 19.6, 19.5, 19.4, 19.2, 19.1, 19.05, 17.5, 17.4, 17.3, 17.2. HR-MS m/z: 828.5066 [M+H]$^+$, calcd. for $C_{46}H_{66}N_7O_7^+$: 828.5018.

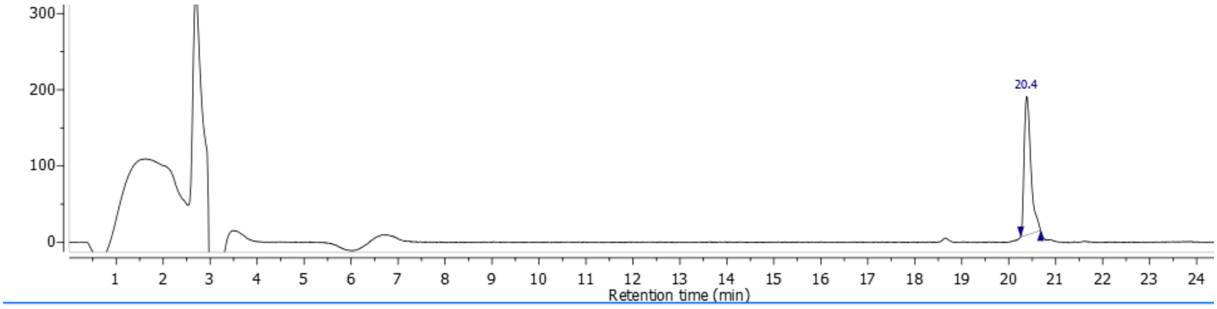

Figure S67. RP-HPLC traces of pure dolastatin analog **11f**.

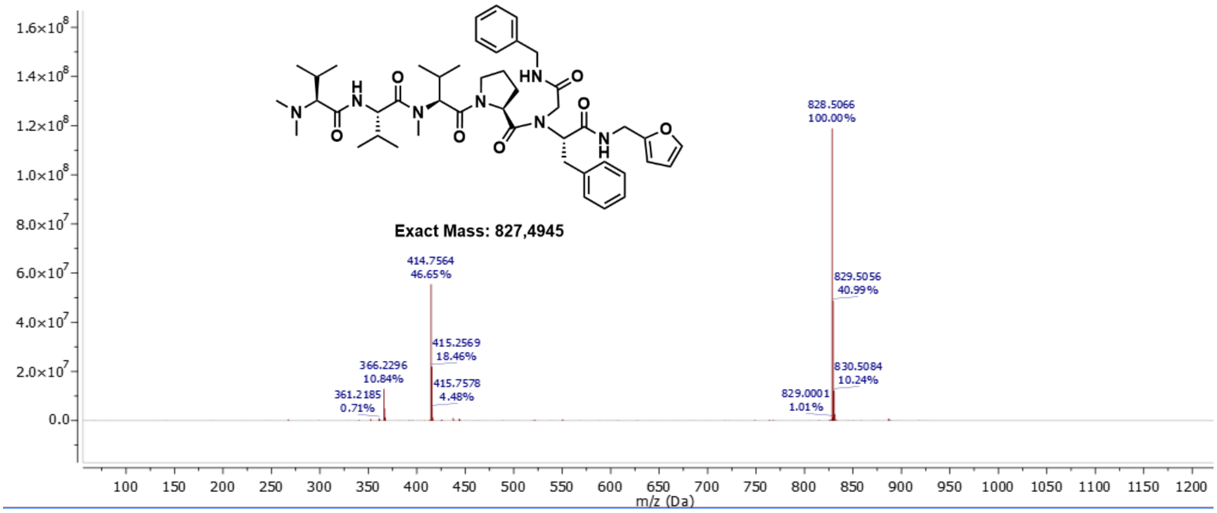

Figure S68. ESI-HRMS of dolastatin analog **11f**.

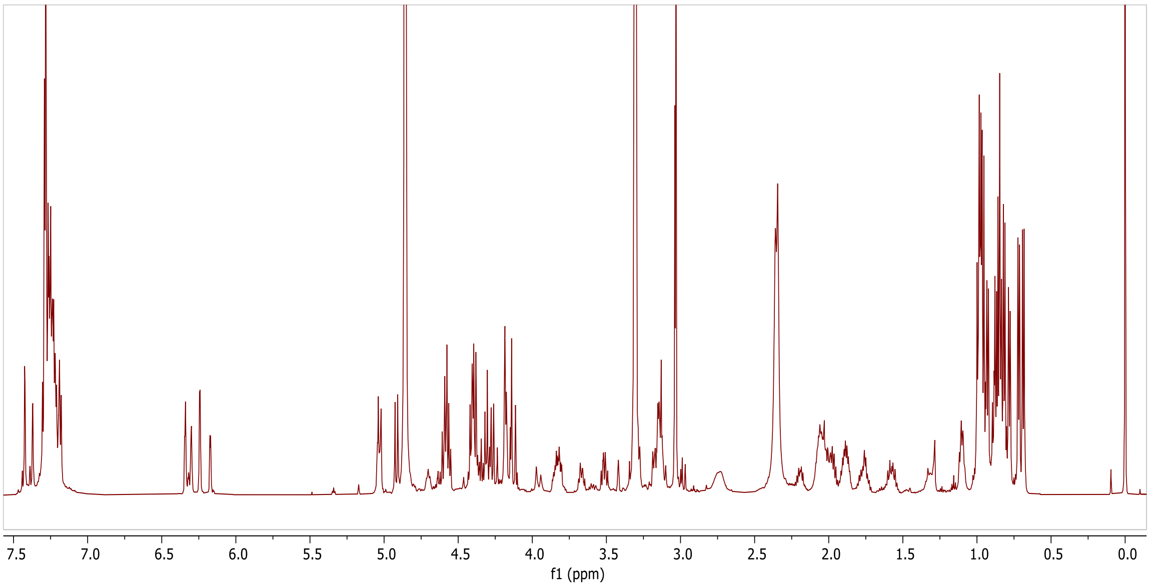

Figure S69. ^{1}H NMR spectrum in CD_3OD of dolastatin analog **11f**.

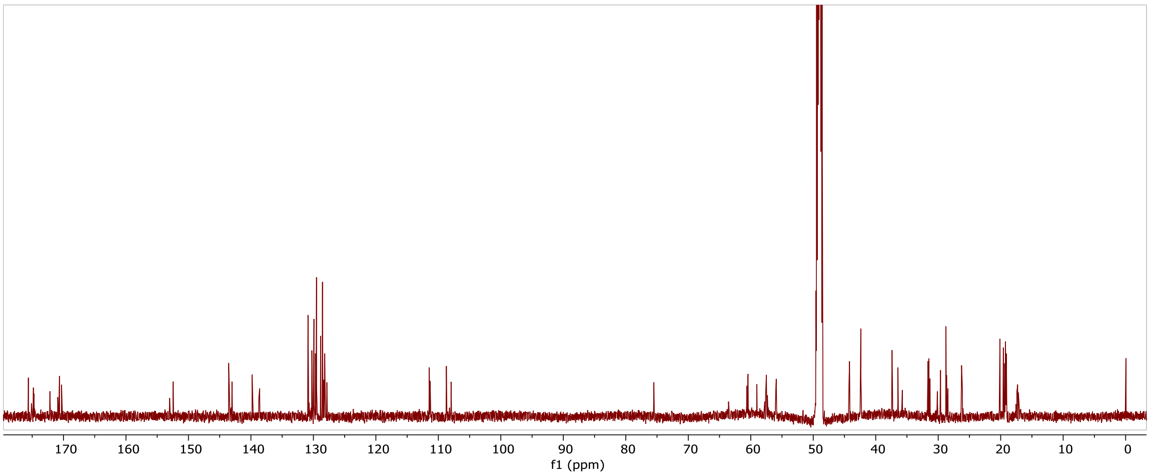

Figure S70. ^{13}C NMR spectrum in CD_3OD of dolastatin analog **11f**.

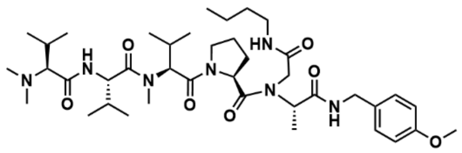

Compound **11g** was obtained as an amorphous white solid (8.9 mg, 22% isolated yield, 99% purity). R_t = 15.23 min. ^{1}H NMR (400 MHz, CD_3OD): mixture of rotamers, δ 8.38 (d, J = 9.1 Hz, 1H), 7.21 (d, J = 8.1 Hz, 1H), 6.87 (d, J = 8.6 Hz, 1H), 5.06 (m, 1H), 4.79 (m, 1H), 4.45 (m, 1H); 4.26-4.21 (m, 1H), 4.11, 4.09 (d, J = 16.6 Hz, 1H); 3.95-3.91 (m, 1H), 3.87 (d, J = 16.8 Hz, 1H); 3.77 (s, 3H), 3.74-3.69 (m, 1H), 3.66 (m, 1H), 3.58-3.51 (m, 1H), 3.28-3.20 (m, 1H), 3.18; 3.14 (s, 3H), 2.70-2.64 (m, 1H), 2.58 (s, 6H), 2.50-2.45 (m, 1H), 2.29-2.48 (m, 4H), 2.12-2.03 (m, 4H), 1.92-1.80 (m, 3H), 1.56-1.48 (m, 2H), 1.39-1.34 (m, 2H), 1.22 (d, J = 7.1 Hz, 3H), 1.03-1.01 (2×d, 6H), 0.95-0.92 (2×d, 6H), 0.91-0.88 (m, 6H), 0.79 (d, J = 6.9 Hz, 3H). ^{13}C NMR (100.6 MHz, CD_3OD): δ 175.0, 174.5, 173.4, 172.5, 170.6, 170.5, 160.5, 131.8, 130.1, 115.0, 114.9, 74.9, 60.6, 58.5, 58.1, 56.3, 55.7, 53.2, 51.4, 47.2, 46.5, 43.9, 42.4, 40.3, 36.4, 35.2, 32.4, 31.7, 31.4, 30.7, 30.5, 30.4, 28.7, 28.6, 28.5, 26.3, 26.2, 21.1, 20.0, 19.5, 19.4, 19.1, 18.1, 14.1. ESI-MS *m/z*: 758.5160 [M+H]$^+$, calcd. for $C_{40}H_{68}O_7N_7$: 758.5180.

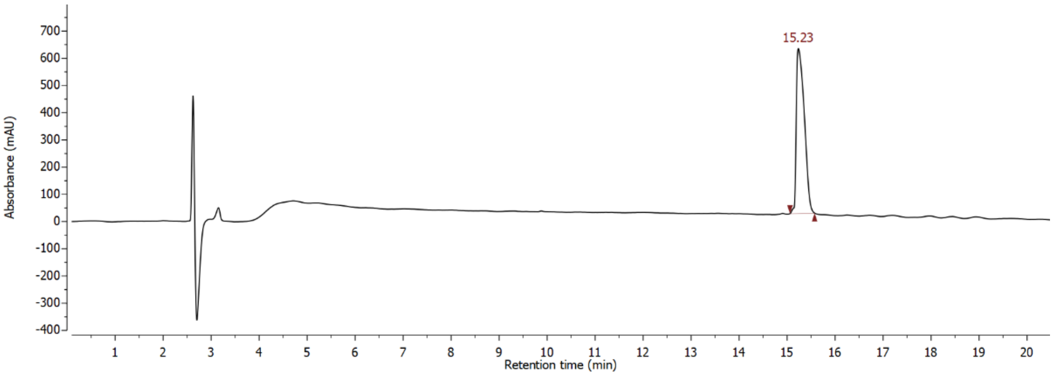

Figure S71. RP-HPLC chromatogram of pure dolastatin analog **11g**.

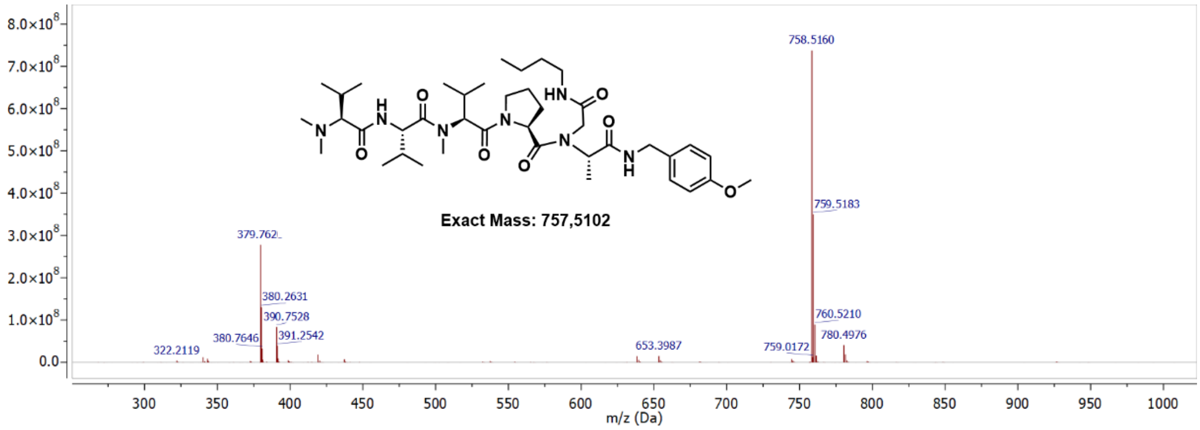

Figure S72. ESI-HRMS spectrum of dolastatin analog **11g**.

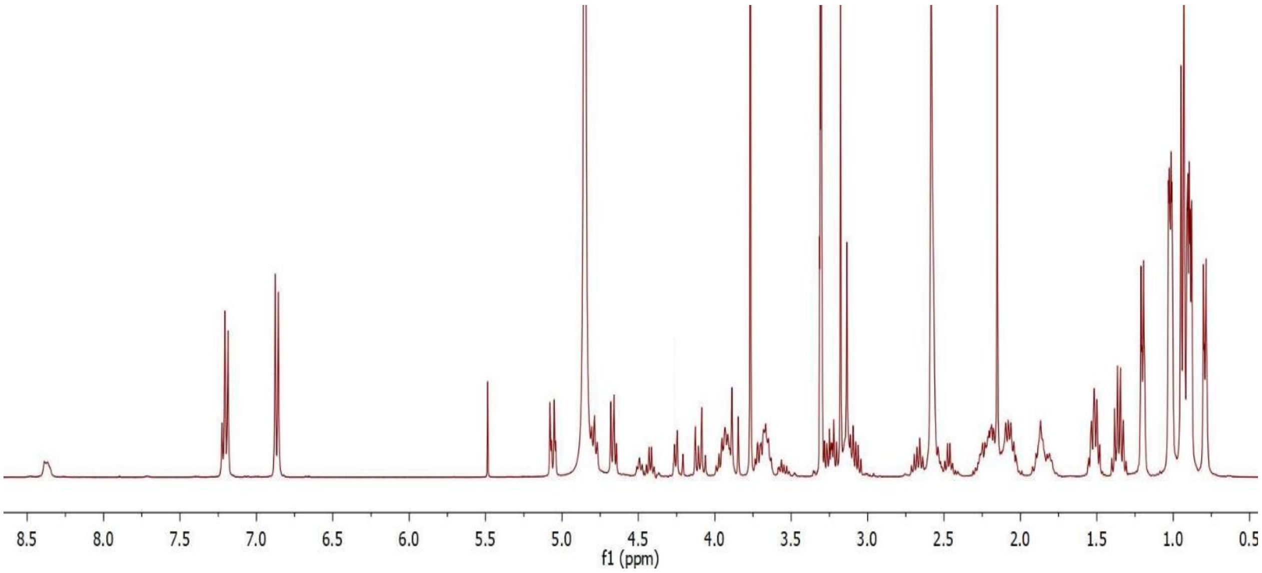

Figure S73. ^{1}H NMR spectrum in CD_3OD of dolastatin analog **11g**.

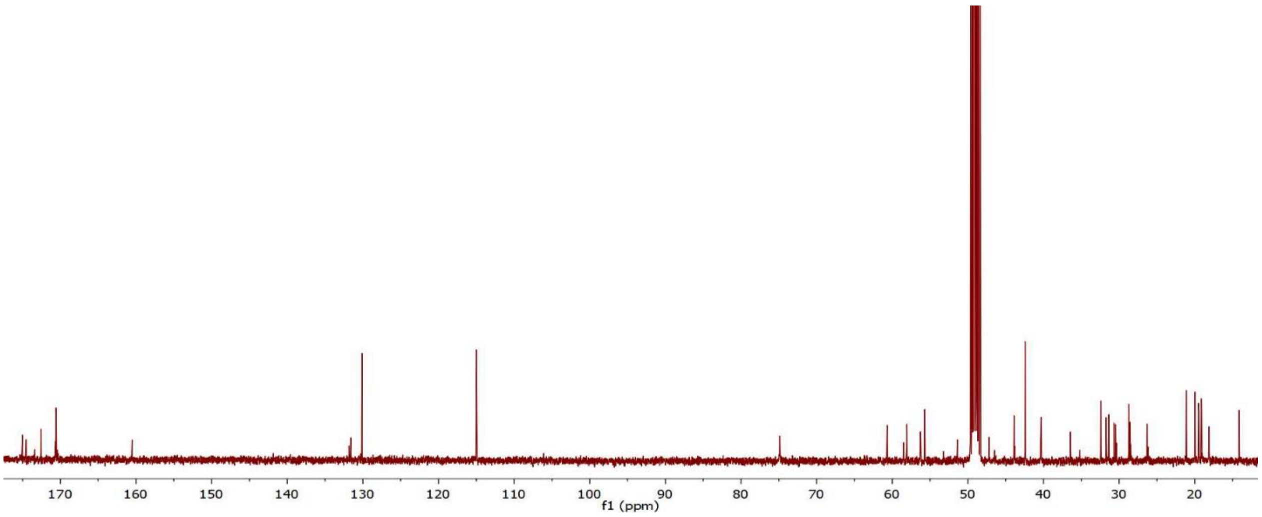

Figure S74. ^{13}C NMR spectrum in CD_3OD of dolastatin analog **11g**.

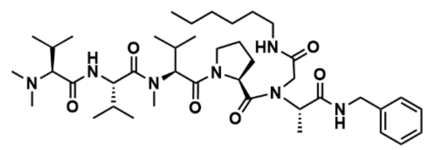

Compound **11h** was obtained as an amorphous white solid (9.3 mg, 24% isolated yield, 98% purity). R_t = 17.02 min. ^{1}H NMR (400 MHz, CD_3OD): mixture of rotamers, δ 7.28 (br. m, 5H), 5.07 (d, J = 11.0 Hz, 1H), 4.79 (m, 1H), 4.45 (m, 1H); 4.28 (m, 1H), 4.15 (d, J = 16.8 Hz, 1H); 3.95-3.91 (m, 2H), 3.85 (d, J = 16.7 Hz, 1H); 3.74-3.63 (m, 2H), 3.59-3-53 (m, 1H), 3.28-3.22 (m, 1H), 3.18, 3.14 (s, 3H), 2.70-2.65 (m, 1H), 2.62-2.57 (m, 1H), 2.47 (s, 6H), 2.23-2.05 (m, 6H), 1.90-1.82 (m, 2H), 1.54-1.50 (m, 2H), 1.35-1.31 (m, 2H), 1.23 (d, J = 7.0 Hz, 3H), 1.02, 1.00 (2×d, J = 7.2 Hz, 6H), 0.93-0.91 (m, 6H), 0.89, 0.87 (2×d, J = 7.1 Hz, 6H), 0.80 (d, J = 6.7 Hz, 3H). ^{13}C NMR (100 MHz, CD_3OD): δ 175.1, 174.7, 174.5, 172.7, 170.6, 170.4, 139.9, 129.6, 128.7, 128.3, 75.2, 60.7, 58.5, 58.1, 56.1, 53.2, 51.4, 47.2, 46.5, 44.4, 42.4, 40.7, 36.4, 35.2, 32.7, 31.7, 31.4, 30.7, 30.5, 30.4, 30.2, 28.7, 28.6, 27.7, 26.3, 23.7, 23.6, 20.0, 19.5, 19.2, 19.1, 19.0, 18.7, 14.4. ESI-MS m/z: 756.5380 [M+H]$^+$, calcd. for $C_{41}H_{70}O_6N_7$: 756.5388.

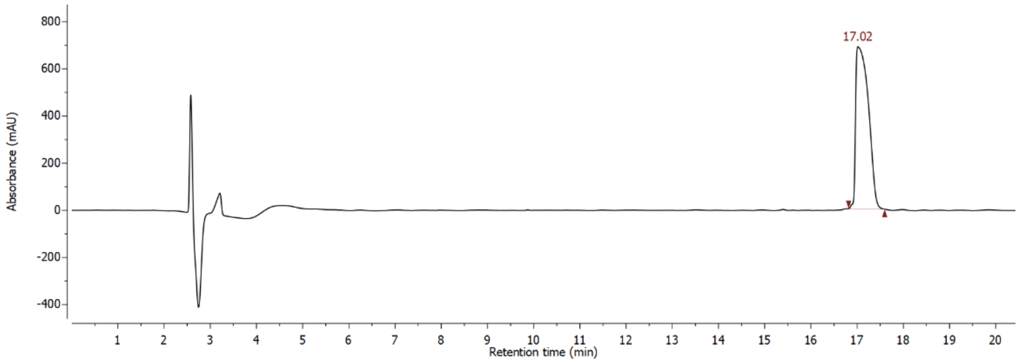

Figure S75. RP-HPLC chromatogram of pure dolastatin analog **11h**.

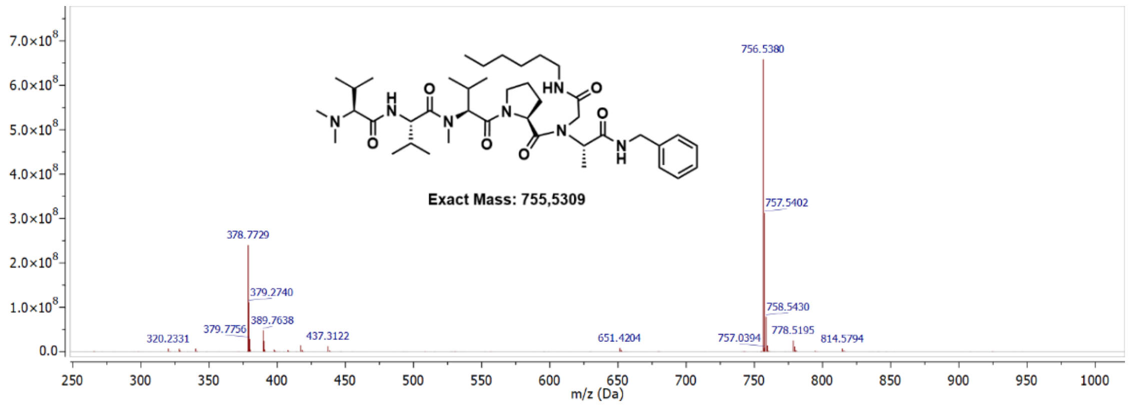

Figure S76. ESI-HRMS spectrum of dolastatin analog **11h**.

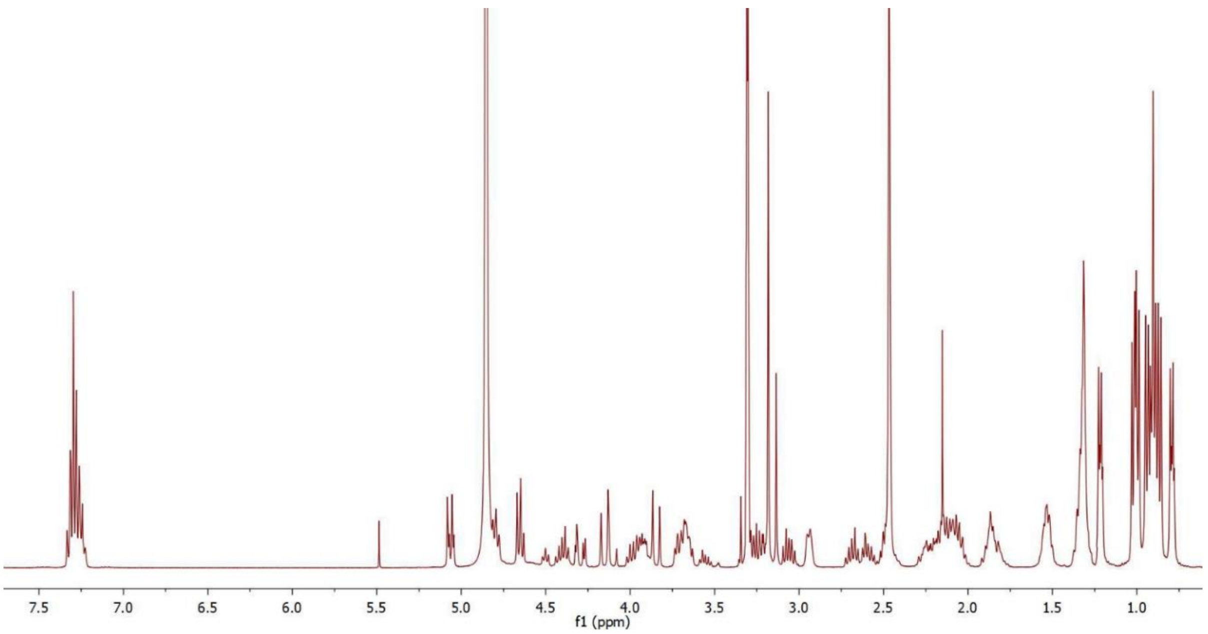

Figure S77. ^{1}H NMR spectrum in CD_3OD of dolastatin analog **11h**.

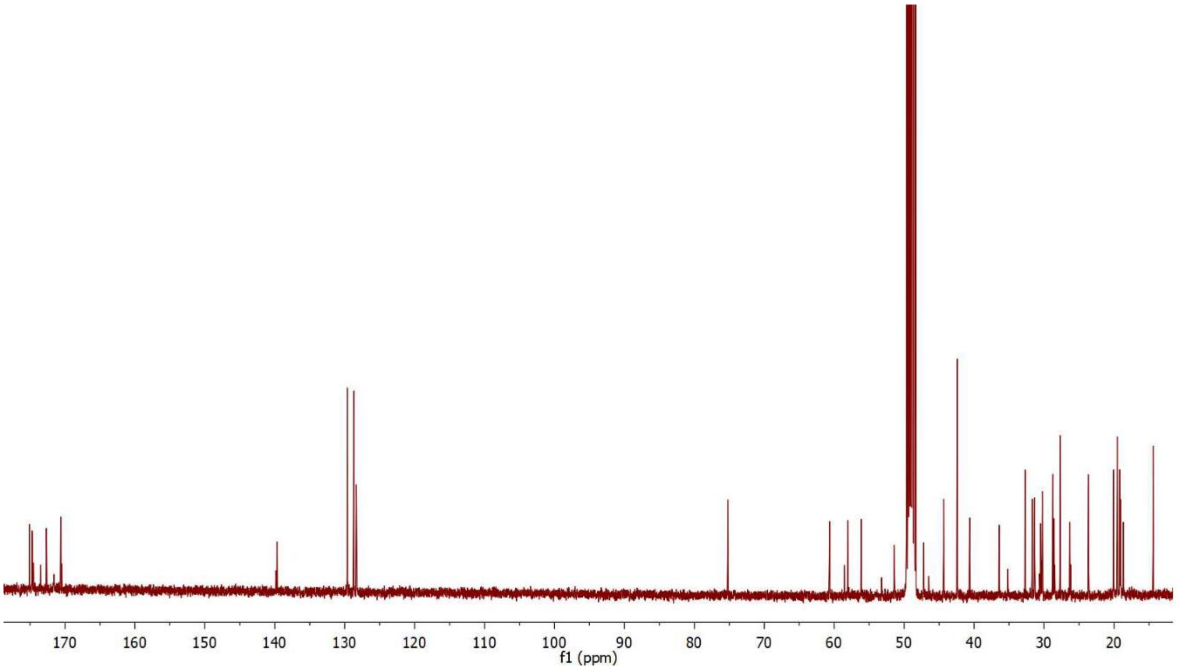

Figure S78. ^{13}C NMR spectrum in CD_3OD of dolastatin analog **11h**.

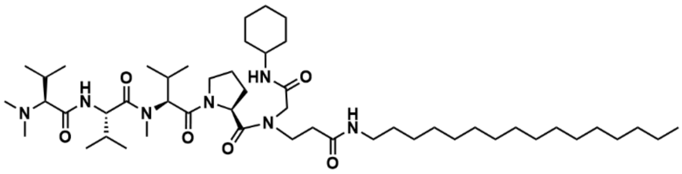

Compound **11i** was obtained as an amorphous white solid (10.2 mg, 18%, >95% purity). R_t = 29.48 min. ^{1}H NMR (400 MHz, CD_3OD): mixture of rotamers, δ 5.08 (dd, J = 12.3, 10.9 Hz, 1H), 4.77 (t, J = 7.2 Hz, 1H), 4.64 (dd, J = 9.8, 8.5 Hz, 1H), 4.51 (t, J = 7.2 Hz, 1H), 4.27, 4.13 (d, J = 17.8 Hz, 1H), 4.13; 3.86 (d, J = 17.7 Hz, 1H), 3.98-3.92 (m, 1H), 3.87 (m, 1H), 3.71-3.63 (m, 3H), 3.22, 3.20 (s, 3H), 3.19-3.10 (m, 2H), 2.66 (m, 1H), 2.63-2.59 (m, 1H), 2.54-2.49 (m, 1H), 2.46-2.39 (m, 1H), 2.30 (s, 6H), 2.27-2.20 (m, 1H), 2.12-2.09 (m, 1H), 2.07-2.01 (m, 2H), 1.93-1.82 (m, 3H), 1.78-1.73 (m, 2H), 1.66 (m, 1H), 1.50 (m, 2H), 1.40-1.14 (m, 26H), 1.02, 1.01 (d, J = 6.7 Hz, 3H), 0.98-0.88 (m, 12H), 0.84, 0.83 (d, J = 6.6 Hz, 3H), 0.81 (d, J = 6.7 Hz, 3H). ^{13}C NMR (CD_3OD): δ 75.7, 60.7, 58.6, 58.2, 56.1, 53.3, 53.2, 51.45, 51.4, 50.2, 49.4, 49.0, 47.3, 46.5, 42.5, 40.7, 36.5, 36.4, 35.3, 33.8, 33.7, 33.6, 31.8, 31.5, 30.85, 33.8, 30.6, 30.40, 28.9, 26.8, 28.6, 28.1, 26.8, 26.4, 26.35, 26.3, 26.2, 23.8, 20.2, 19.7, 19.5, 19.4, 19.35, 19.2, 14.5. ESI-MS *m/z*: 888.7254 [M+H]$^+$, calcd. for $C_{50}H_{95}O_6N_7$: 888.7266.

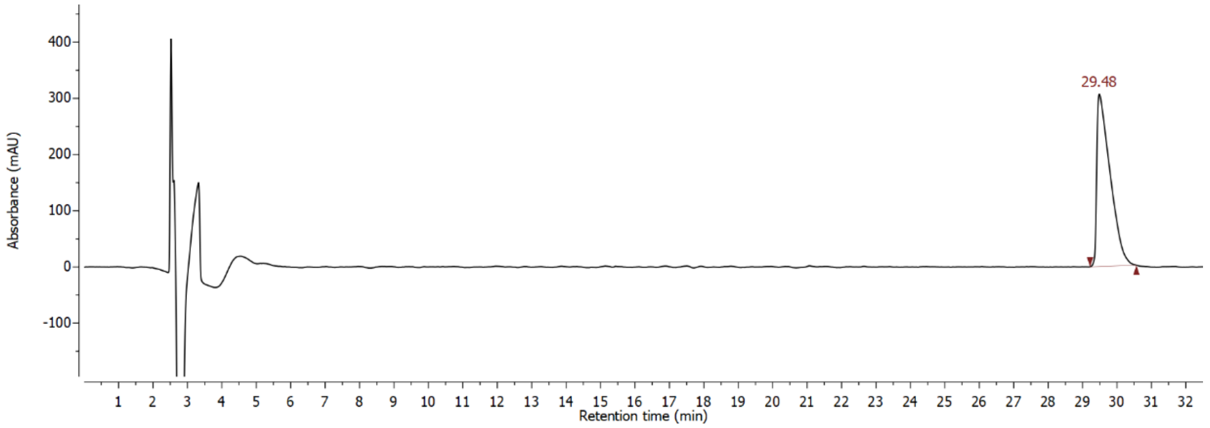

Figure S79. RP-HPLC chromatogram of pure dolastatin analog **11i**.

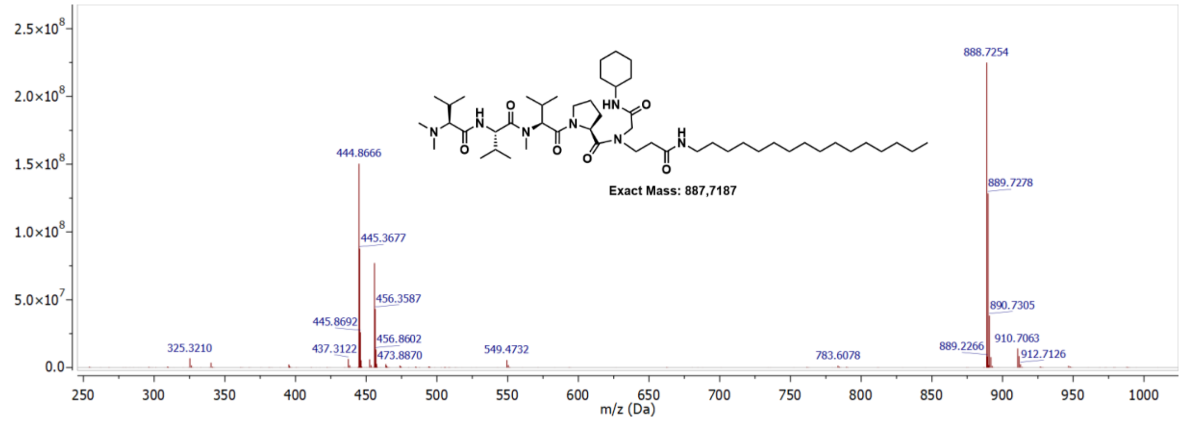

Figure S80. ESI-HRMS spectrum of dolastatin analog **11i**.

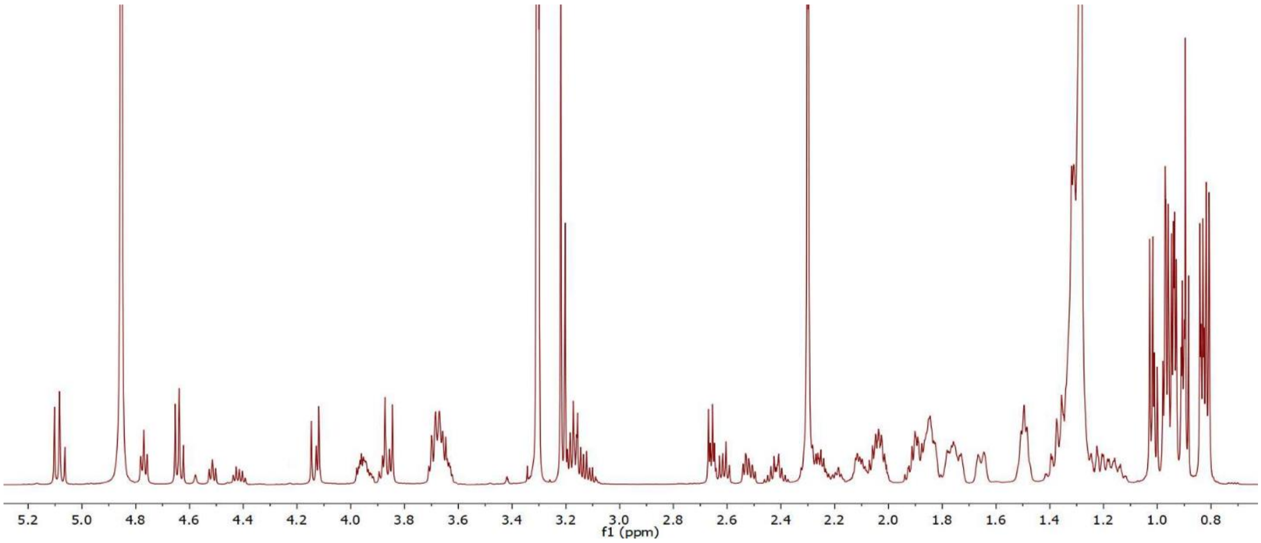

Figure S81. ^{1}H NMR spectrum in CD_3OD of dolastatin analog **11i**.

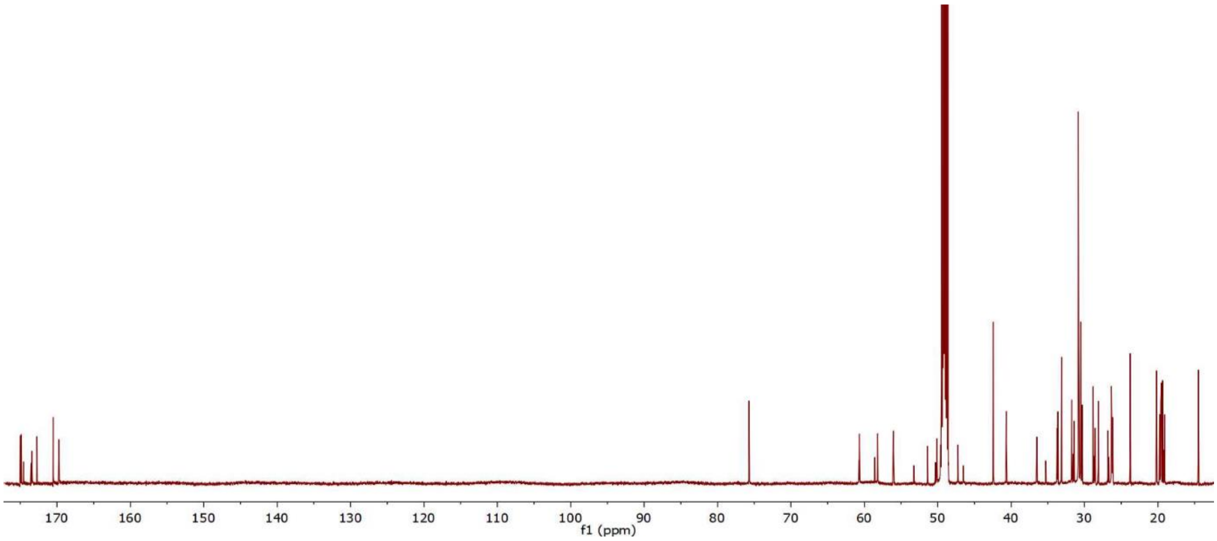

Figure S82. ^{13}C NMR spectrum in CD_3OD of dolastatin analog **11i**.

Material and methods for cell-based assays

Human cell culture materials and cultivation methodology

The *in vitro* cell viability and cytotoxicity assays were performed with the human prostate cancer cell line PC-3, the human colorectal cancer cell line HCT-116 and the human triple-negative breast cancer cell line MDA-MB-468; the fluorescence microscopic inspections just with PC-3 cells. The cell lines were obtained from DSMZ (Braunschweig, Germany) and ATCC (Manassas, VA, USA) and cultivated in their specific growth medium. PC-3 cells were cultivated in RPMI 1640 supplemented with 1% (v/v) glutamine, and 10% (v/v) heat-inactivated fetal calf serum (FCS); HCT-116 was cultured in McCoy's 5a Modified Medium supplemented with 10% (v/v) heat-inactivated FCS; and for MDA-MB-468, DMEM high glucose (4.5 g/L) supplemented with 10% (v/v) heat-inactivated FCS was used.

All the basal culture media and supplements, like glutamine and FCS, PBS and 0.05% trypsin-EDTA for cell detachment were purchased from Capricorn Scientific GmbH (Ebsdorfergrund, Germany). Cell culture plastics were used from TPP (Trasadingen, Switzerland), Greiner Bio-One (Frickenhausen, Germany) and Sarstedt (Nürnbrecht, Germany). Digitonin was purchased from Riedel De Haën (Seelze, Germany) and DMSO from Duchefa Biochemie (Haarlem, The Netherlands). Resazurin was used from Sigma-Aldrich Chemie GmbH (Taufkirchen, Germany). The tubulin-specific dye reagent SiR-tubulin kit (SC002) and verapamil were purchased from Spirochrome AG (Stein am Rhein, Switzerland), the nuclei stain H33342 was from Thermo Fisher Scientific (Waltham, MA, USA).

Cultivation was routinely done in T-75 flasks in a humidified atmosphere with 5% CO_2 at 37 °C until subconfluency (~ 70-80%) was reached, and the cells were ready for subsequent sub-culturing or assay usage. For that, the adherent cells were rinsed with PBS and detached with trypsin/EDTA (0.05% in PBS) prior to cell passaging and seeding.

Cell viability assay - methodology

The impacts of the newly synthesized dolastatin ~~derivatives~~ analogs on the viability and proliferation of the three human cancer cell lines were investigated by performing fluorometric resazurin-based cell viability assays, following a protocol well established in our lab.[3,4] In

[3] R. Kufka, R. Rennert, G. N. Kaluđerović, L. Weber, W. Richter, L. A. Wessjohann, *Beilstein J. Org. Chem.* **2019**, *15*, 96-105
[4] Y. T. H. Lam, J. Hoppe, Q. N. Dang; A. Porzel, A. Soboleva, W. Brandt, R. Rennert, H. Hussain, M. D. Davari, L. Wessjohann, N. Arnold, *J. Nat. Prod.* **2023**, *86*, 1373-1384.

brief, the cells were seeded in low densities in 96-well plates (PC-3: 3.000 cells/well; HCT-116: 5.000 cells/well; MDA-MB-468: 10.000 cells/well) yielding a seeding confluency of ~ 10 – 20%, and were allowed to adhere overnight. Subsequently, the cells were treated for 48 h with dilutions series (25.6 pM – 10 µM in 5-fold dilutions) of the test compounds (1 mM DMSO stocks). For control measures, cells were treated in parallel with 1% DMSO (negative control, representing the final DMSO content of the highest concentrated sample point, for data normalization set to 100% cell viability) and 100 µM digitonin (positive control, for data normalization set to 0% cell viability). After finalization of the 48 h incubation, the incubation media were discarded, and cells were rinsed once with PBS. Resazurin solution in basal medium was prepared freshly prior to use and added to the cells in a final resazurin concentration of 50 µM. Subsequently, the cells were incubated under standard growth conditions for further 2 h. Finally, the conversion of resazurin to resorufin by the remaining viable cells was fluorometrically measured (λexc. 540 nm / λem. 590 nm) by using a SpectraMax iD5 multiwell plate reader (Molecular Devices, San Jose, USA). Data were determined in at least triplicates. IC_{50} curves and values were calculated by using a four parametric function and GraphPad Prism v10.1 software (San Diego, CA, USA).

Fluorescence microscopy

PC-3 prostate cancer cells were seeded in 10-well chamber slides at a density of 5 × 10^4 cells per well in 100 µL. The cells were then cultivated for 24 hours in a humidified atmosphere at 37°C with 5% CO_2 (standard growth conditions) to allow cell attachment. Subsequently, the cells were treated for 48 h under standard growth conditions with the dolastatin analogs' IC_{50}, i.e. 1.5 µM of **5a** and 0.03 µM of **5h** (as indicated in Table 1), in 100 µL of PC-3 culture medium. Negative and positive controls were included, where cells were treated with either just with PC-3 culture medium (negative control) or the tubulysin derivative tubugi-4 (positive control, treatment with IC_{50} = 0.5 nM).[5] After the 48 hours treatment, the impact of the test items on the cellular tubulin network was inspected by using fluorescence microscopy. For that purpose, tubulin was stained with SiR-tubulin (SC002) following the manufacturer's instructions. In brief, the cells were washed twice with PBS, then stained for 1 hour under standard growth conditions with a solution containing 1 µM SC002 and 10 µM verapamil, a broad-spectrum efflux pump inhibitor, in PC-3 medium.[6] Subsequently, the cells were washed

[5] D. Llanes, R. Rennert, P. Jänicke, I. Morgan, L. Reguera, D. G. Rivera, M. G. Ricardo, L. A. Wessjohann, *Front. Pharmacol.* **2024**, *15*, 1408091.
[6] S. Gupta, K. A. Cohen, K. Winglee, M. Maiga, B. Diarra, W. R. Bishai, *Antimicrobial Agents and Chemotherapy* **2014**, *58* (1), 574–576.

with PBS and stained with 1 µM Hoechst 33342 in PC-3 medium for 10 minutes. After staining, the cells were washed twice using PBS and finally inspected under 100 µL of PC-3 medium by using an EVOS FL Auto fluorescence microscope (Thermo Fisher Scientific, Waltham, MA, USA) and the Cy5 filter set for SiR-tubulin and the DAPI filter set for H33342. Images were processed by using ImageJ software.

Results of cell viability assays

The cytotoxic/cytostatic activity of the synthesized compounds was *in vitro* tested against the human PC-3 (prostate cancer), HCT-116 (colorectal cancer) and MDA-MB-468 (triple-negative breast cancer, TNBC) cell lines by performing fluorometric resazurin-based cell viability assays. The IC_{50} values calculated based on dose-response curves are summarized in Table 1 and Table 2 of the main paper.

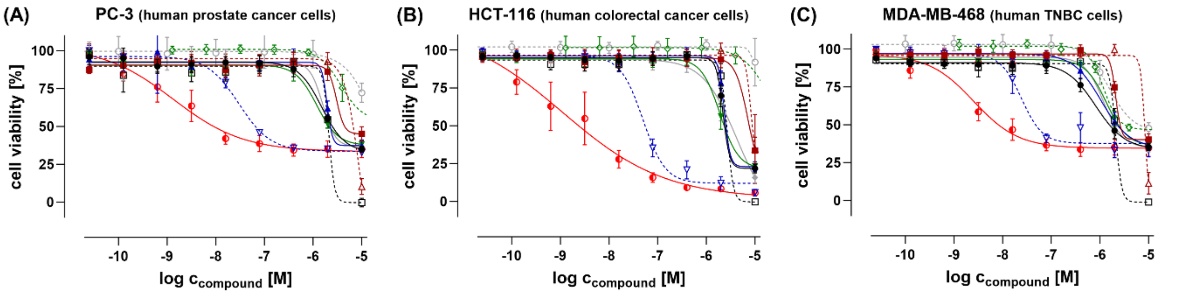

	IC_{50} value (95% CI) PC-3	IC_{50} value (95% CI) HCT-116	IC_{50} value (95% CI) MDA-MB-468
**5a**	1.5 µM (1.2 - 1.9 µM)	2.2 µM (nc - 3.6 µM)	0.82 µM (0.66 - 1.0 µM)
**5b**	~ 5 µM (nc)	7.2 µM (nc)	2.1 µM (nc - 2.7 µM)
**5c**	1.9 µM (nc - 3.9 µM)	2.3 µM (nc)	1.1 µM (0.85 - 1.3 µM)
**5d**	1.2 µM (1.0 - 1.4 µM)	1.9 µM (1.7 - 2.3 µM)	1.4 µM (1.2 - 1.5 µM)
**5e**	1.6 µM (1.1 - nc µM)	4.3 µM (nc)	1.2 µM (1.0 - 1.5 µM)
**5f**	2.1 µM (nc - 3.1 µM)	2.7 µM (nc)	2.1 µM (nc - 2.7 µM)
**5g**	8.9 µM (nc)	8.3 µM (nc)	~ 5 µM (nc)
**5h**	0.03 µM (0.026 - 0.034 µM)	0.05 µM (0.043 - 0.056 µM)	0.024 µM (nc - 0.032 µM)
**5i**	3.7 µM (2.9 - 4.9 µM)	> 10 µM (nc)	~ 5 µM (nc)
**5j**	> 10 µM (nc)	> 10 µM (nc)	~ 10 µM (nc)
**Cemadotin**	0.0013 µM (0.0003 - 0.003 µM)	0.0011 µM (nc - 0.004 µM)	0.0025 µM (0.0015 - 0.0037 µM)

Figure S83. Dose-response curves and IC_{50} values of the **5**-series compounds as determined based on resazurin-based cell viability assays testing human PC-3, HCT-116 and MDA-MB-468 cancer cells and as calculated using GraphPad Prism v10.1 software. Cells were treated for 48 hours. Error bars indicate the 95% confidence interval (CI); 95% CI values are given in brackets, as far the software was able to calculate them based on the data (nc – not calculable). See also Table 1 in the manuscript.

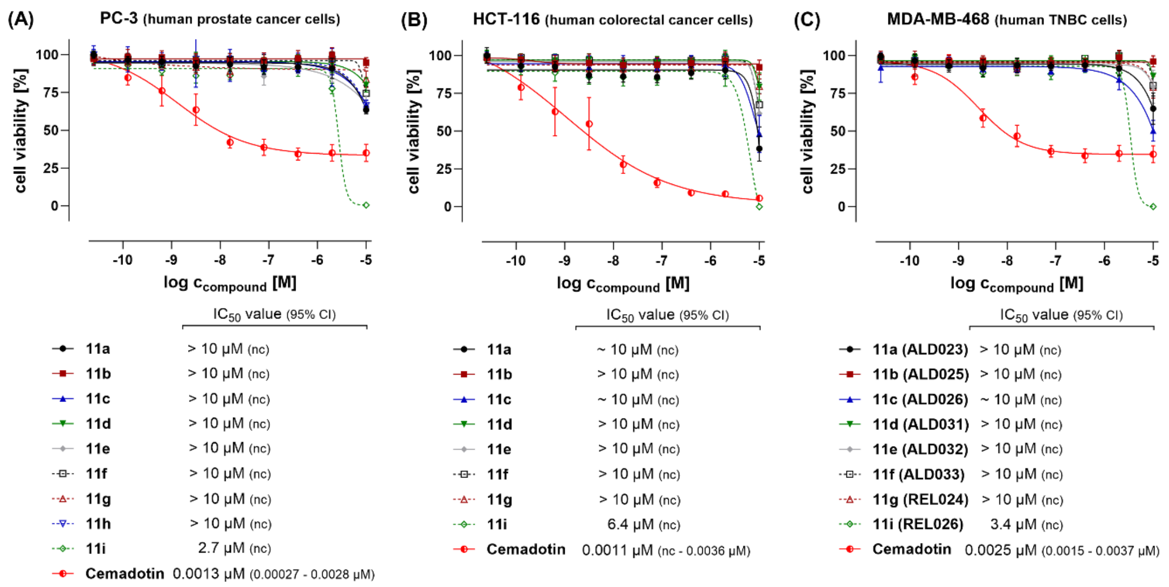

Figure S84. Dose-response curves and IC_{50} values of the **11**-series compounds as determined based on resazurin-based cell viability assays testing human PC-3, HCT-116 and MDA-MB-468 cancer cells and as calculated using GraphPad Prism v10.1 software. Cells were treated for 48 hours. Error bars indicate the 95% confidence interval (CI); 95% CI values are given in brackets, as far the software was able to calculate them based on the data (nc – not calculable). Compound **11h** was exclusively tested in PC-3 but not in HCT-116 and MDA-MB-468 cells. See also Table 2 in the manuscript.

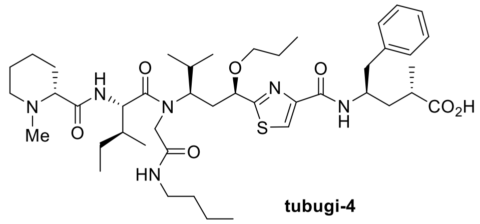

tubugi-4

Figure S85. Structure of the tubulysin analogue tubugi-4 used in the Fluorescence microscopy study in comparison with the dolastatin analogs.
